# Pan-Cancer Analysis, Reveals COVID-19-Related BSG as a Novel Marker for Treatment and Identification of Multiple Human Cancers

**DOI:** 10.3389/fcell.2022.876180

**Published:** 2022-05-13

**Authors:** Tao Huang, Wei-Ying He

**Affiliations:** ^1^ Department of Cardiothoracic Vascular Surgery, The Affiliated Hospital of Youjiang Medical University for Nationalities, Baise, China; ^2^ The First Clinical Medical College, The First Affiliated Hospital of Guangxi Medical University, Nanning, China

**Keywords:** cancer, prognosis, prediction, immunology, target therapy

## Abstract

**Background:** Coronavirus disease 2019 (COVID-19) has been a public threat and healthcare concern caused by severe acute respiratory syndrome coronavirus 2 (SARS-CoV-2) infection. During the period of the pandemic of COVID-19, cancer patients should be paid more attention as more severe events are found in cancer patients infected with SARS-CoV-2. Basigin (*BSG*) is an essential factor for the infection and progression of COVID-19 and tumorigenesis of multiple tumors, which may serve as a novel target for the effective treatment against COVID-19 and multiple human cancers.

**Methods:** A total of 19,020 samples from multiple centers were included in our research for the comprehensive investigation of the differences in BSG expression among human organs, cancer cells, cancer tissues, and normal tissues. Cox regression analysis and Kaplan–Meier curves were utilized to explore the prognosis factor of *BSG* in cancers. Correlation analyses were used to determine associations of *BSG* expression with tumor mutational burden, the immune microenvironment, etc. Gene set enrichment analysis was applied to explore the underlying mechanisms of *BSG* in cancers.

**Results:** Compared with normal tissues, *BSG* expression was high in 13 types of cancers (cholangiocarcinoma, etc.) and low in colon adenocarcinoma and rectum adenocarcinoma. *BSG* expression was related to the prognosis of eight cancers (e.g., invasive breast carcinoma) (*p* < 0.05). The gene also demonstrated a pronounced effect in identifying 12 cancers (cholangiocarcinoma, etc.) from their control samples (AUC >0.7). The *BSG* expression was associated with DNA methyltransferases, mismatch repair genes, immune infiltration levels, tumor mutational burden, microsatellite instability, neoantigen, and immune checkpoints, suggesting the potential of *BSG* as an exciting target for cancer treatment. *BSG* may play its role in several cancers by affecting several signaling pathways such as drug cytochrome metabolism P450 and JAK-STAT.

**Conclusion:** *BSG* may be a novel biomarker for treating and identifying multiple human cancers.

## Introduction

Coronavirus disease 2019 (COVID-19) has been a public threat and healthcare concern caused by severe acute respiratory syndrome coronavirus 2 (SARS-CoV-2) infection and spread by respiratory droplets through coughing or sneezing among the contacts (Li Q et al., 2020). During the period of the pandemic of COVID-19, cancer patients should be paid increasing attention as more severe events are found in cancer patients infected with SARS-CoV-2 (Madariaga et al., 2020; Moujaess, Kourie, and Ghosn 2020). Exploring strategies to control the epidemic of SARS-CoV-2 in cancer patients will benefit the continue safe treatment for these patients during the pandemic of COVID-19 (Madariaga et al., 2020).

Basigin (*BSG*) is a transmembrane glycoprotein of the immunoglobulin superfamily, which is also known as cluster of differentiation 147 (CD147), extracellular matrix metalloproteinase inducer (EMMPRIN), and so on. According to recent reports, *BSG* serves as a target that correlated with SARS-CoV-2 spike protein that can allow SARS-CoV-2 to enter host cells through endocytosis and even initiate the MAPK pathway through the spike protein/CD147/CyPA” signaling axis and induce cytokine storm (Wang K et al., 2020; Fenizia et al., 2021; Geng et al., 2021; Behl et al., 2022). It is also reported that the existence of humanized anti-CD147 antibodies can inhibit SARS-CoV-2 and its variants entering host cells (Geng et al., 2021). Therefore, *BSG* is a crucial element for the infection and progression of COVID-19 and may serve as a novel target for effective treatment against COVID-19 (Wang K et al., 2020; Behl et al., 2022).

In addition to COVID-19, *BSG* is also reported to be involved in the tumorigenesis of multiple tumors, such as melanoma (Kim et al., 2021; Lu et al., 2021), liver hepatocellular carcinoma (LIHC) (Wang SJ et al., 2020), and hypopharyngeal squamous cell carcinoma (Suzuki et al., 2021). *BSG* has been found to induce the degradation of the extracellular matrix through the matrix metalloproteinase proteins and stimulates the vascular endothelial growth factor, thus participating in the invasion and metastasis of tumors (Weidle et al., 2010; Kim et al., 2021). It is also reported that T cells and NK cells carrying a chimeric antigen receptor that recognizes *BSG* is detrimental to the tumor cell lines *in vitro* (Tseng et al., 2020). The inhibition of *BSG* can reduce the growth, migration, and invasion of tumor cells (Hatanaka et al., 2016; Lian et al., 2017; Dana et al., 2020). Therefore, *BSG* is a potential predictive biomarker for tumors (Hatanaka et al., 2016; Qiao et al., 2020; Tseng et al., 2020), which may serve as a biological modulator of tumor and COVID-19 and improve the management of cancer patients during the period of the COVID-19 pandemic (Varadarajan et al., 2020). However, *BSG* expression and its relation with clinical relevance and immune infiltration levels in pan-cancer were rarely reported, which requires further investigation.

Based on analyzing thousands of specimens from several high-throughput databases, including Genotype-Tissue Expression (GTEx), Cancer Cell Line Encyclopedia (CCLE), and The Cancer Genome Atlas (TCGA), this study comprehensively measures the *BSG* expression level and its potential clinical significance in pan-cancer. Moreover, the study also investigated the association of *BSG* with alternative genes and immune infiltration levels in cancers, contributing to the understanding of *BSG* as a novel and potential biomarker for the identification and treatment of multiple cancers.

## Materials and Methods

### Collection of *BSG* mRNA Expression, Clinical Characteristics, and Prognosis Data

GTEx Consortium (2013) includes plenty of normal tissue specimens of *Homo sapiens*, and CCLE (Ghandi et al., 2019) contains numerous samples of various cell lines of cancers. Transcriptome data (for assessing the expression of *BSG* in normal tissues and cancer lines) of GTEx and CCLE were collected from GTEx Portal (8,671 samples) and DepMap Portal (459 specimens), respectively (Supplementary Table S1). TCGA collects transcriptome and clinical data of 32 kinds of cancers, and 9,054 cancers and 727 controls of TCGA were downloaded from the Xena database; among the 32 cancers, twelve types (including 2,075 samples) had less than three corresponding controls and were not utilized for investigating the difference in *BSG* expression between normal tissues and cancer tissues. Three types of samples of TCGA were eventually included in this study, containing primary tumor tissue, normal solid tissue, and primary blood-derived cancer peripheral blood. All gene expression levels in data obtained from GTEx, CCLE, and TCGA were processed in R (v4.1.0) using log_2_ (*x* + 1).

Three kinds of clinical characteristics—American Joint Committee on Cancer (AJCC), age, and gender—were obtained from the Xena database. Four types of survival data—overall survival (OS), disease-specific survival (DSS), disease-free interval (DFI), and progression-free interval (PFI)—of cancer patients were downloaded from the Xena database. In order to avoid the impact of short-term follow-up time and small sample size, the inclusion criteria of prognostic information for survival analysis were set as follows: 1) the prognostic information of patients with follow-up time not less than 30 days and 2) cancers with at least three cancer samples. The included clinical characteristics and prognosis data can be viewed in Supplementary Table S1.

### Collection of BSG Protein Level Data

Immunohistochemical staining data for validating the BSG protein levels in cancer and normal tissues were obtained from The Human Protein Atlas (proteinatlas.org) (Uhlen et al., 2015) by using the “HPAanalyze” package (Tran et al., 2019) in R (v4.1.0). The BSG protein levels of cancers with no fewer than two samples in both normal tissues and cancer tissues were chosen in this study. For one cancer with more than one kind of antibody, data on antibodies used to detect more samples of this cancer were included. Eventually, 109 samples were selected for further analysis, and these samples’ identity documents, groups, and tissue types were stored in Supplementary Table S2. Normal tissues and tumor tissues were obtained from various individuals, and no person provided both normal and tumor samples. Additionally, three normal lung specimens were utilized twice (one for lung adenocarcinoma [LUAD] and the other for lung squamous cell carcinoma [LUSC]) for comparing the difference in the BSG protein levels between normal and cancer tissues (Supplementary Table S2). A bladder cancer (BLCA) tissue sample and a PRAD tissue sample were collected from the same patient with these two diseases (Supplementary Table S2). The BSG protein levels were evaluated with the criteria (listed in Supplementary Table S3) for staining intensity and quantity scores. The BSG protein levels were evaluated by the total immunohistochemical score, the product of the staining intensity, and quantity scores. All protein level scores for the 109 samples collected from The Human Protein Atlas are shown in Supplementary Table S2.

### Alternations, DNA Methyltransferases, and the Immune Microenvironment of *BSG* in Cancers

Alternations of *BSG* in multiple cancers were explored based on the data (processed by MuTect2 software) of the GDC Portal, and this step was performed in Sanger Box (v3.0). DNA methyltransferases (DNMTs) can influence gene expression and genetic performance without changing the DNA sequence. The expression relationships of *BSG* with three typical DNMTs—DNMT1, DNMT3A, and DNMT3B—were investigated in this study.

Based on the deconvolution method, the TIMER (Li et al., 2017; Li T et al., 2020) algorithm makes it feasible to calculate the immune abundance of six kinds of immune cells (B cells, CD4+ T cells, CD8+ T cells, neutrophil, macrophage, and dendritic cells) by whole-gene expression data. The other algorithm, ESTIMATE (Yoshihara et al., 2013), enables researchers to evaluate the immune abundance of immune cells and immune stromal based on gene expression data matrix. With the ESTIMATE algorithm, the abundance of immune stromal, immune cells, and tumor purity are reflected by the stromal (for stromal cells), immune (for immune cells), and ESTIMATE scores (for tumor purity). The TIMER and ESTIMATE data (Supplementary Table S1) for investigating the correlation of *BSG* expression with the immune microenvironment were collected from TIMER’s official website and SangerBox 3.0, respectively.

### The Expression of *BSG* With Mismatch Repair Genes, Tumor Mutational Burden, Microsatellite Instability, Neoantigen Count, and Immune Checkpoint Genes

Mismatch repair genes (MMRs) participate in repairing errors of DNA replications, and the lack of MMRs can lead to more somatic mutations. The expression levels of five MMRs (MLH1, MSH2, MSH6, PMS2, and EPCAM) and forty-six immune checkpoints (BTLA, etc.) were extracted from TCGA dataset. For cancer patients included in TCGA dataset, data on tumor mutational burden (TMB), microsatellite instability (MSI), and neoantigen count (including single-nucleotide variation and indel neo-antigens) have been published by the previous study (Liu et al., 2018) and were applied in this research.

### The Potential Mechanisms of BSG in Pan-Cancer and the Underlying Target Drugs for the Gene

Using the “clusterProfiler” package (Yu et al., 2012), the study investigated potential mechanisms of *BSG* in 32 cancers based on KEGG (Kyoto Encyclopedia of Genes and Genomes) signaling pathways by gene set enrichment analysis (GSEA). For essential signaling pathways showing statistical significance in multiple cancers, data of their leading-edge genes were extracted to explore these genes’ correlations with *BSG* expression, which verify the relation of *BSG* with the essential signaling pathways. Using CellMiner (Reinhold et al., 2015) and based on half-maximal inhibitory concentration (IC50) values, this research also detected the sensitivity of *BSG* to 57 drugs approved by the Food and Drug Administration of America or validated by clinical tests.

### Statistical Analysis

The Kruskal–Wallis test was selected to assess the difference in *BSG* expression in various normal tissues and cancer cell lines. The Wilcoxon rank-sum test was used to assess the differential expression of BSG (at both mRNA and protein levels) between cancer tissues and their controls. Multiple comparisons by the false discovery rate method were applied to verify the distinct expression levels of *BSG* mRNA between cancers and controls. The homogeneity between cancer and control groups was evaluated using the chi-square test or Fisher’s exact test.

The Wilcoxon rank-sum test was utilized to explore the relationship between *BSG* expression and the three clinical features (AJCC stage, age, and gender). Additionally, if the *BSG* expression was associated with not less than two clinical parameters (e.g., age and gender), the adjustment test would be performed and the correlation between the *BSG* expression and age would be analyzed hierarchically based on various genders.

Using “survival” and “forestplot” packages in R (v4.1.0), univariate Cox regression analysis and Kaplan–Meier plots were applied to measure the relevance between *BSG* expression and OS, DSS, DFI, and PFI. The optimal cut point for high- and low-*BSG* expression levels in each Kaplan–Meier curve was determined by utilizing the maximally selected rank statistics from the “maxstat” and “survminer” R packages. For detecting the accuracy of *BSG* expression in identifying cancers from controls, the specificity, sensitivity, and the area under the curve (AUC) of the receiver operating characteristics (ROC) were calculated using the “pROC” package (Robin et al., 2011) for R (v4.1.0), and the specificity, sensitivity, and the AUC of a summary ROC were determined in Stata (v15.0).

The Spearman rank correlation analyses were applied to evaluate the correlations of *BSG* expression with DNMTs, immune environment, TMB, MSI, neoantigen count, immune checkpoints, and leading-edge genes. The Wilcoxon rank-sum test was utilized to determine the difference in abundance of six kinds of immune cells and the difference in IC50 of certain drugs between high- and low-*BSG* expression groups.

**FIGURE 1:**
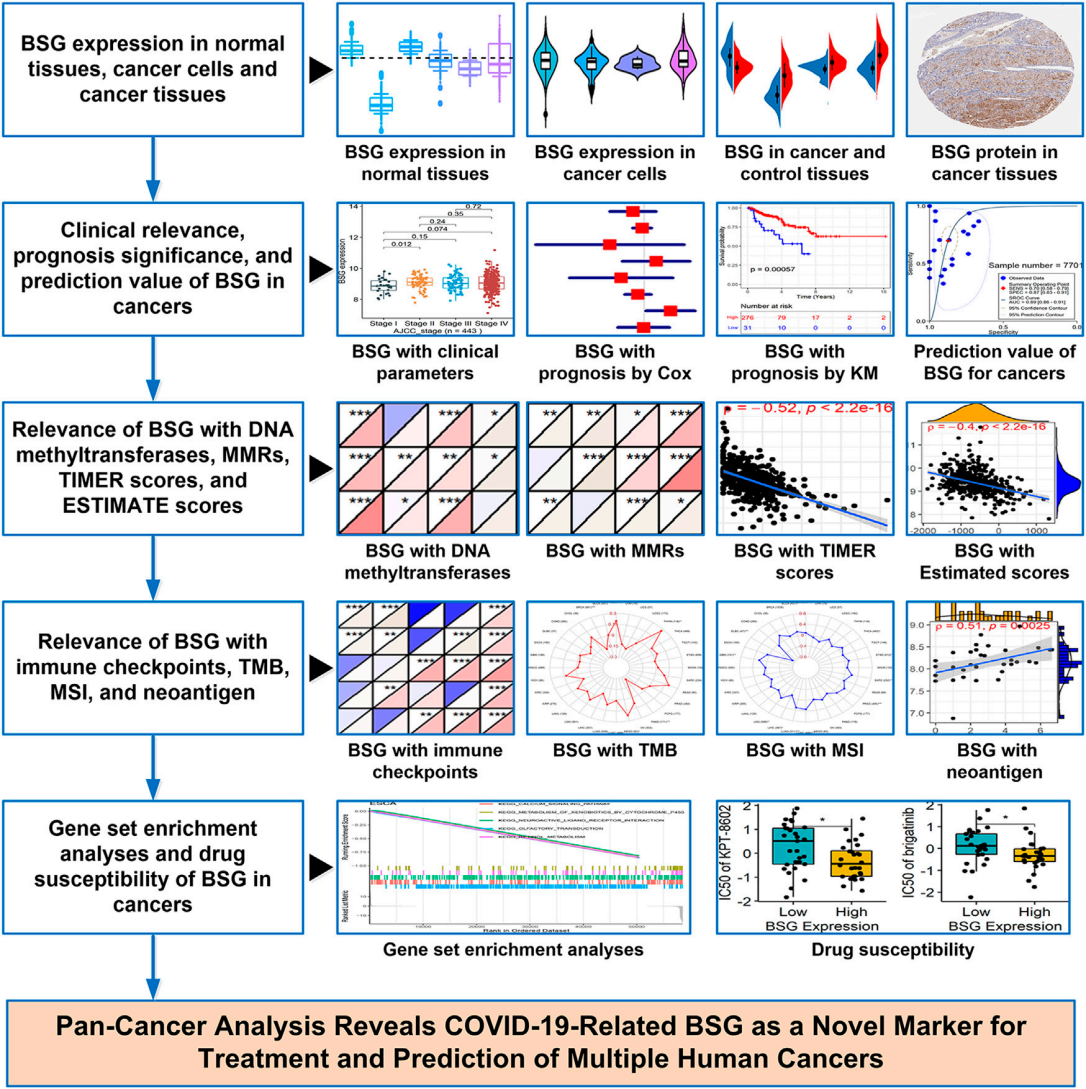
Overflow of this study. BSG, basigin; “Cox” represents Cox regression analysis; “KM” represents Kaplan–Meier curves; MMRs, mismatch repair genes; TMB, tumor mutational burden; MSI, microsatellite instability.

In this study, *p*-value < 0.05 demonstrated statistical significance, and except for drug-sensitive analysis, all *p*-values were two-sided. An overflow of the study can be viewed in Figure 1.

## Results

### Differently Expressed *BSG* in Human Cancers

**FIGURE 2:**
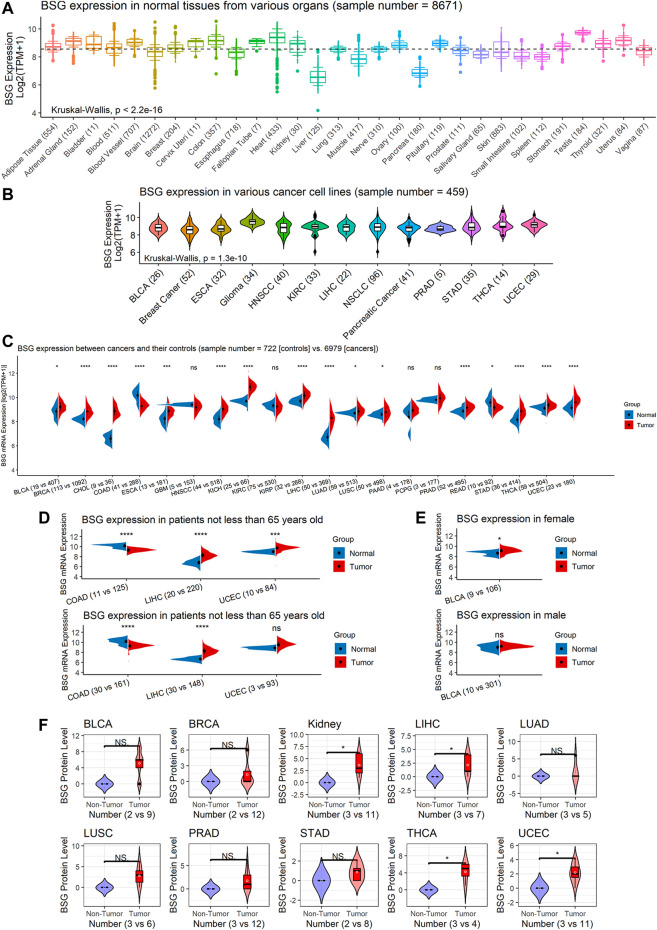
BSG expression in normal tissues and pan-cancer at mRNA and protein levels. **(A)** Distinct BSG expression in various normal tissues of humans; the dashed line represents the average value (equaling to 8.549) of BSG expression in all normal tissues shown in this panel. **(B)** Different BSG expressions in various cancer cell lines of humans. **(C)** Different BSG expressions between cancer tissues with their normal tissues. **(D–E)** Hierarchical analysis of BSG expression differences in some cancers and their control tissues. **(F)** Elevated BSG levels in several cancers were validated at the protein level. In **(A,B)**, the value in parentheses represents the number of samples. In **(C–E)**, the two values (e.g., “n = 19 vs. 407” for BLCA) in parentheses represent the numbers of controls and cancers, respectively. In **(C–F)**, each *p*-value in this figure is based on the Wilcoxon rank-sum test, and then *p*-values in **(C–E)** are adjusted by multiple comparisons with the false discovery rate method; ^*^ *p* < 0.05, ^**^ *p* < 0.01, ^***^ *p* < 0.001. BLCA, bladder cancer; ESCA, esophageal carcinoma; HNSCC, head and neck squamous cell carcinoma; KIRC, kidney renal clear cell carcinoma; LIHC, liver hepatocellular carcinoma; NSCLC, non-small-cell lung carcinoma; PRAD, prostate adenocarcinoma; STAD, stomach adenocarcinoma; THCA, thyroid carcinoma; UCEC, uterine corpus endometrial carcinoma; BRCA, invasive breast carcinoma; CHOL, cholangiocarcinoma; COAD, colon adenocarcinoma; GBM, glioblastoma multiforme; KICH, kidney chromophobe; KIRP, kidney renal papillary cell carcinoma; LUAD, lung adenocarcinoma; LUSC, lung squamous cell carcinoma; PCPG, pheochromocytoma and paraganglioma; READ, rectum adenocarcinoma.

Based on GTEx data, the *BSG* expression was various in different organs its high expression was observed in normal tissues of the adrenal gland, heart, uterus, and so on, while significantly low expression of *BSG* was found in the liver and pancreas (*p* < 0.05; Figure 2A). An analysis based on CCLE data demonstrated the different distribution of *BSG* in various cancer cell lines (*p* < 0.05; Figure 2B). In the observed 20 cancer types, the *BSG* expression levels of 16 cancers differed from those of the corresponding control samples. In detail, upregulated *BSG* expression was observed in 14 cancers: BLCA, invasive breast carcinoma (BRCA), cholangiocarcinoma (CHOL), esophageal carcinoma (ESCA), head and neck squamous cell carcinoma (HNSCC), kidney chromophobe (KICH), kidney renal papillary cell carcinoma (KIRP), LIHC, LUAD, LUSC, prostate adenocarcinoma (PRAD), stomach adenocarcinoma (STAD), thyroid carcinoma (THCA), and uterine corpus endometrial carcinoma (UCEC); downregulated *BSG* expression was detected in two cancers: colon adenocarcinoma (COAD) and rectum adenocarcinoma (READ) (*p* < 0.05; Figure 2C). Notably, in COAD, LIHC, and UCEC, the age distribution between cancer and control groups was statistically different. Similarly, in BLCA, there was a difference in gender distribution between the cancer group and the control group (Supplementary Table S4 **)**. In order to exclude the potential effects of gender and age covariates in the difference in *BSG* expression between cancer and control groups, a hierarchical evaluation was performed for these four cancers. Increasing levels of *BSG* were observed in female BLCA patients but not in male BLCA patients (Figure 2E). Thus, gender distribution may contribute to the difference in BSG expression in BLCA. Increasing levels of BSG were observed in female BLCA patients but not in male BLCA patients (Figure 2E).

**FIGURE 3:**
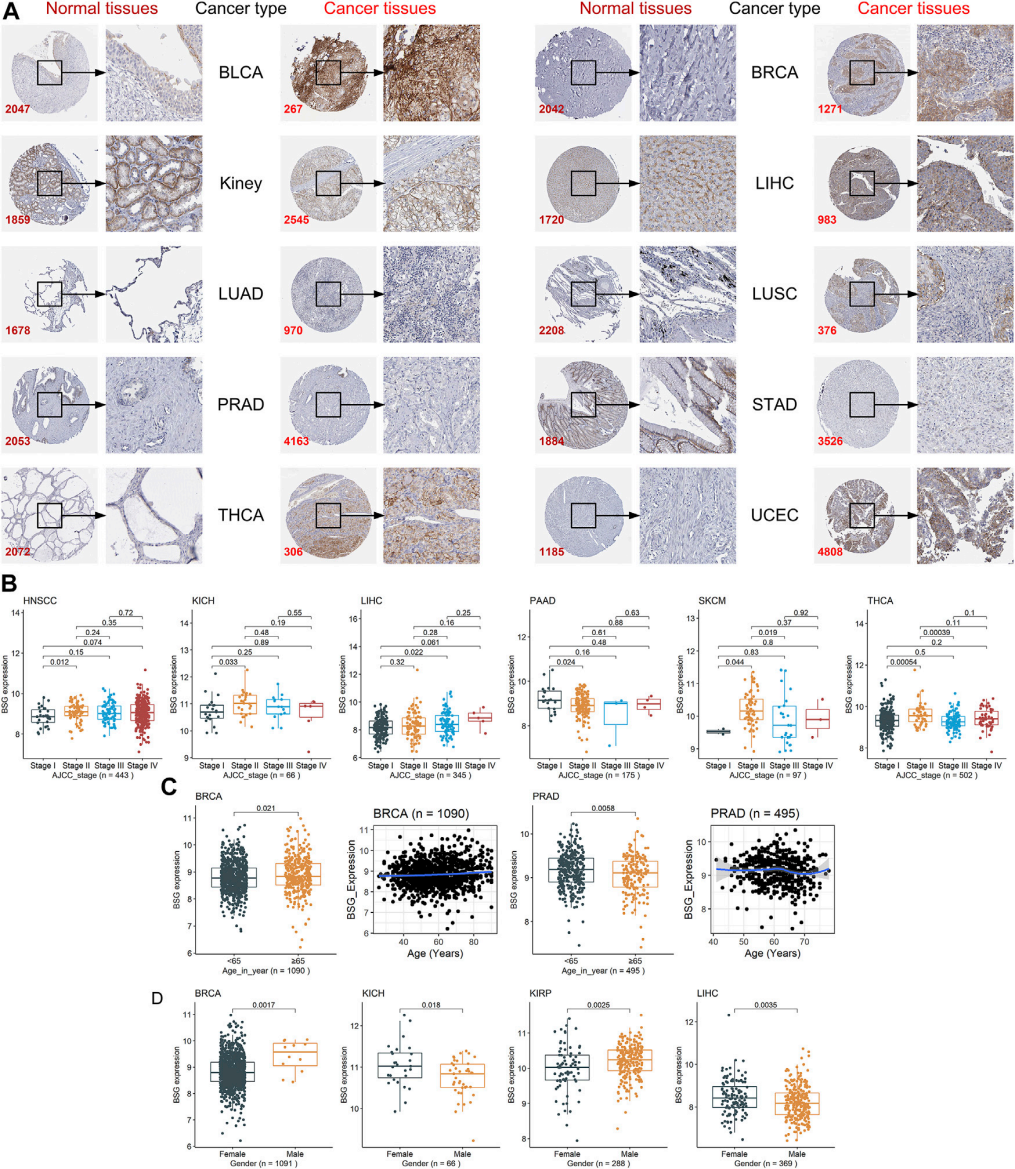
Staining intensity of the anti-BSG antibodies in the ten cancers and the correlations between BSG expression and clinical parameters. **(A)** Under the microscope, the staining intensity of the anti-BSG antibodies in the ten cancer tissues is observed to be stronger than that in normal tissues; images are available from v21.0.proteinatlas.org. In **(A)**, the value of the lower-left corner of the images (e.g., 2047 for BLCA) is the identity document of patients or normal individuals. **(B–D)** Close correlations between BSG expression with clinical parameters; all *p*-values in the three panels are based on the Wilcoxon rank-sum test. The two plots with a blue curve of panel **(C)** are drawn based on the locally weighted regression. The value in parentheses (e.g., “n = 443” for HNSCC) represents the number of samples. BLCA, bladder cancer; BRCA, invasive breast carcinoma; LIHC, liver hepatocellular carcinoma; LUAD, lung adenocarcinoma; LUSC, lung squamous cell carcinoma; PRAD, prostate adenocarcinoma; STAD, stomach adenocarcinoma; THCA, thyroid carcinoma; UCEC, uterine corpus endometrial carcinoma; HNSCC, head and neck squamous cell carcinoma; KICH, kidney chromophobe; PAAD, pancreatic adenocarcinoma; SKCM, skin cutaneous melanoma.

Among the 16 cancers listed earlier, BSG protein levels were investigated in ten types as there were at least two cancer samples and two normal samples for the ten. In these ten kinds of cancers, compared to corresponding normal tissues, upregulated BSG protein levels were detected in kidney cancer (for KICH and KIRP), LIHC, THCA, and UCEC, consistent with the results at mRNA level (*p* < 0.05; Figure 2D). Notably, the THCA and its control groups’ age distribution was statistically different (Supplementary Table S5). However, we failed to perform a hierarchical evaluation for this resulting from a lack of samples, which requires verification in the future. Although there is no statistical significance for other cancers, a trend can be seen that upregulation of BSG protein levels was identified in BLCA, BRCA, LUAD, LUSC, PRAD, and STAD (*p* > 0.05; Figure 2D). Under the microscope, the staining intensity of the anti-BSG antibodies in the ten cancer tissues was observed to be stronger than that in normal tissues (Figure 3A).

### Relevance Between *BSG* Expression and Clinical Parameters

Different clinical parameters may result in the different prognoses of cancer patients. For example, patients with cervical cancer and with advanced AJCC stages tended to have poor prognoses (Olawaiye et al., 2021). The other example was that elder age, and male gender were risk factors for patients with oral squamous cancers (Oh et al., 2021). Thus, the relationship between *BSG* expression and the three clinical parameters—AJCC stages, age, and gender—of patients was explored in neoplasms with the clinical parameter data. The immediate relevance of *BSG* expression with AJCC stages was found in HNSCC, KICH, LIHC, PAAD, SKCM, and THCA (*p* < 0.05; Figure 3B), while such a phenomenon was not in the other cancers (ACC, etc.) (Supplementary Figure S1). Higher *BSG* expression was detected in elderly (65 years old or older) BRCA patients (*p* < 0.05), and based on the LOESS (locally weighted regression) age trajectory, older patients tend to be found increasing the *BSG* expression levels (Figure 3C). Elevated *BSG* expression was generally be observed in young (<65 years old) PRAD patients (*p* < 0.05); however, the LOESS age trajectory suggested some fluctuations in the correlation between age and *BSG* expression (Figure 3C). There was no correlation between *BSG* expression and age in other 30 cancers (e.g., ACC; Supplementary Figure S2). In terms of gender, males with BRCA or KIRP had overexpression of *BSG*, while the contrast phenomenon was found in patients with KICH or LIHC (*p* < 0.05; Figure 3D); and for other cancers (ACC, etc.), there was no statistical difference in *BSG* expression between patients with different genders (Supplementary Figure S3).

Additionally, the adjustment tests were carried out for KICH, LIHC, and BRCA as *BSG* expression was detected related to no less than two clinical parameters in the three cancers. As a result, adjusted for gender, 1) there was no statistical difference in *BSG* expression between KICH patients with various AJCC stages for both females and males, although that was observed in LIHC (Supplementary Figure S4A); and 2) elevated *BSG* expression was detected in the old BRCA patients for female but not for male patients (Supplementary Figure S4B). The conclusion that male BRCA patients tend to have upregulated *BSG* expression was true for not the old but the young (Supplementary Figure S4C). Moreover, after adjusting AJCC stages, differential *BSG* expression was observed between female and male patients with KICH (for stage II) or LIHC (for stage I) (Supplementary Figure S4D).

### Prognosis Value of *BSG* Expression in Cancers

**FIGURE 4:**
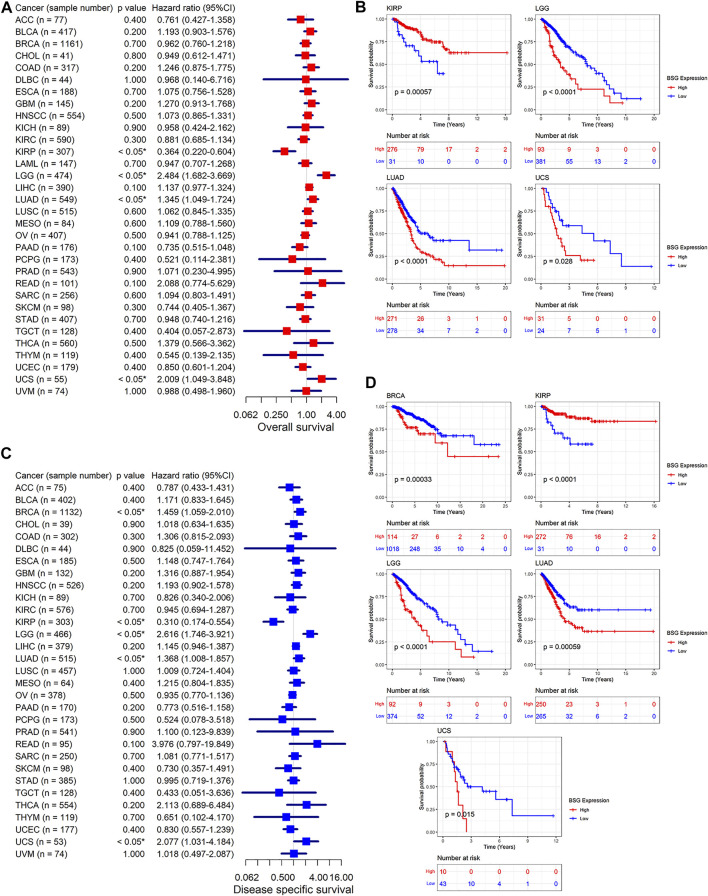
Relation of BSG expression with overall survival ( **(A–B)**) and disease-specific survival of cancer patients ( **(C–D)**). **(A,C)** Forest plots produced by univariate Cox regression analysis. **(B,D)** Kaplan–Meier curves with *p*-values based on log-rank tests. The optimal cut point for high and low expression levels of BSG in each Kaplan–Meier curve was identified using the maximally selected rank statistics from the “maxstat” and “survminer” R packages. In A and C, the value in parentheses (e.g., “n = 77” for ACC) represents the number of samples. ACC, adrenocortical carcinoma; BLCA, bladder cancer; BRCA, invasive breast carcinoma; CHOL, cholangiocarcinoma; COAD, colon adenocarcinoma; DLBC, lymphoid neoplasm diffuse large B-cell lymphoma; ESCA, esophageal carcinoma; GBM, glioblastoma multiforme; HNSCC, head and neck squamous cell carcinoma; KICH, kidney chromophobe; KIRC, kidney renal clear cell carcinoma; KIRP, kidney renal papillary cell carcinoma; LAML, acute myeloid leukemia; LGG, brain lower grade glioma; LIHC, liver hepatocellular carcinoma; LUAD, lung adenocarcinoma; LUSC, lung squamous cell carcinoma; MESO, mesothelioma; OV, ovarian serous cystadenocarcinoma; PAAD, pancreatic adenocarcinoma; PCPG, pheochromocytoma and paraganglioma; PRAD, prostate adenocarcinoma; READ, rectum adenocarcinoma; SARC, sarcoma; SKCM, skin cutaneous melanoma; STAD, stomach adenocarcinoma; TGCT, testicular germ cell tumors; THCA, thyroid carcinoma; THYM, thymoma; UCEC, uterine corpus endometrial carcinoma; UCS, uterine carcinosarcoma; UVM, uveal melanoma.

**FIGURE 5:**
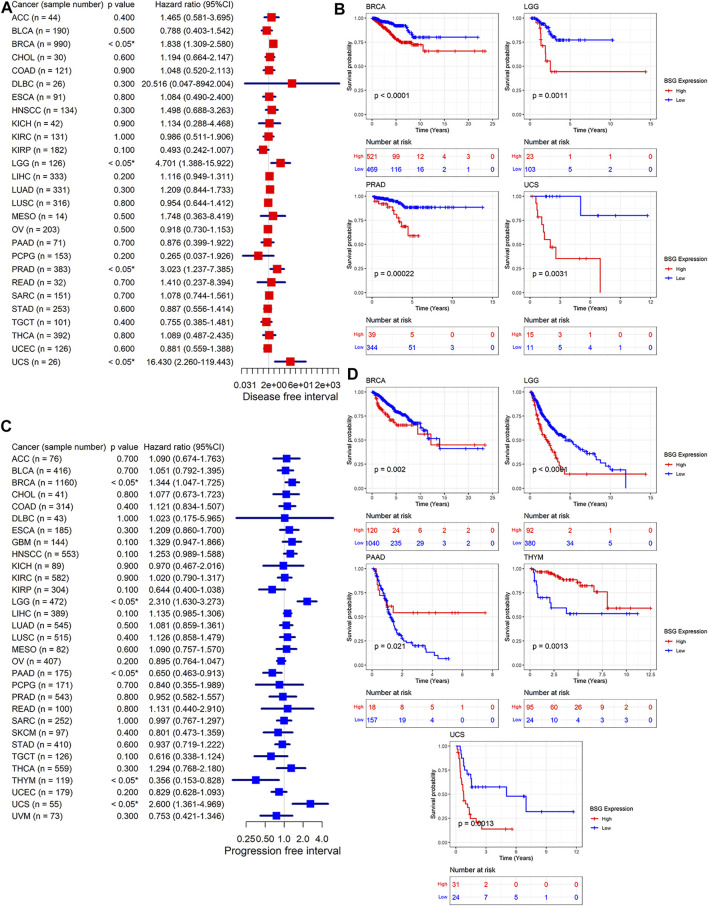
Relation of BSG expression with disease-free survival **(A–B)** and progression-free survival of cancer patients **(C–D)**. **(A,C)** Forest plots produced by univariate Cox regression analysis. **(B,D)** Kaplan–Meier curves with *p*-values based on log-rank tests. The optimal cut point for high and low expression levels of BSG in each Kaplan–Meier curve was identified using the maximally selected rank statistics from the “maxstat” and “survminer” R packages. In **(A,C)**, the value in parentheses (e.g., “n = 44” for ACC) represents the number of samples. ACC, adrenocortical carcinoma; BLCA, bladder cancer; BRCA, invasive breast carcinoma; CHOL, cholangiocarcinoma; COAD, colon adenocarcinoma; DLBC, lymphoid neoplasm diffuse large B-cell lymphoma; ESCA, esophageal carcinoma; GBM, glioblastoma multiforme; HNSCC, head and neck squamous cell carcinoma; KICH, kidney chromophobe; KIRC, kidney renal clear cell carcinoma; KIRP, kidney renal papillary cell carcinoma; LGG, brain lower grade glioma; LIHC, liver hepatocellular carcinoma; LUAD, lung adenocarcinoma; LUSC, lung squamous cell carcinoma; MESO, mesothelioma; OV, ovarian serous cystadenocarcinoma; PAAD, pancreatic adenocarcinoma; PCPG, pheochromocytoma and paraganglioma; PRAD, prostate adenocarcinoma; READ, rectum adenocarcinoma; SARC, sarcoma; SKCM, skin cutaneous melanoma; STAD, stomach adenocarcinoma; TGCT, testicular germ cell tumors; THCA, thyroid carcinoma; THYM, thymoma; UCEC, uterine corpus endometrial carcinoma; UCS, uterine carcinosarcoma; UVM, uveal melanoma.

We investigated the prognosis value of *BSG* expression in various cancers. Based on the results of univariate Cox analyses and Kaplan–Meier curves, in terms of both OS and DSS, the *BSG* expression was relevant to shorter survival time in LGG, LUAD, and UCS (hazard ratio [HR] > 1, *p* < 0.05) and to longer survival time in KIRP (HR = 0.364, *p* < 0.05) (Figures 4A–D). Moreover, high *BSG* expression was also a risk factor of DSS for BRCA patients (HR = 1.459, *p* < 0.05) (Figures 4C,D). In DFI, overexpression of *BSG* represented unfavorable prognosis for patients with BRCA, LGG, PRAD, and UCS (HR > 1, *p* < 0.05) (Figures 5A,B). In PFI, increased *BSG* expression was associated with poor PFI for BRCA, LGG, and UCS (HR > 1, *p* < 0.05), while it demonstrated a favorable prognosis for PAAD and THYM (Thymoma) patients (HR < 1, *p* < 0.05) (Figures 5C,D).

### Identification Value of *BSG* Expression in Cancers

**FIGURE 6:**
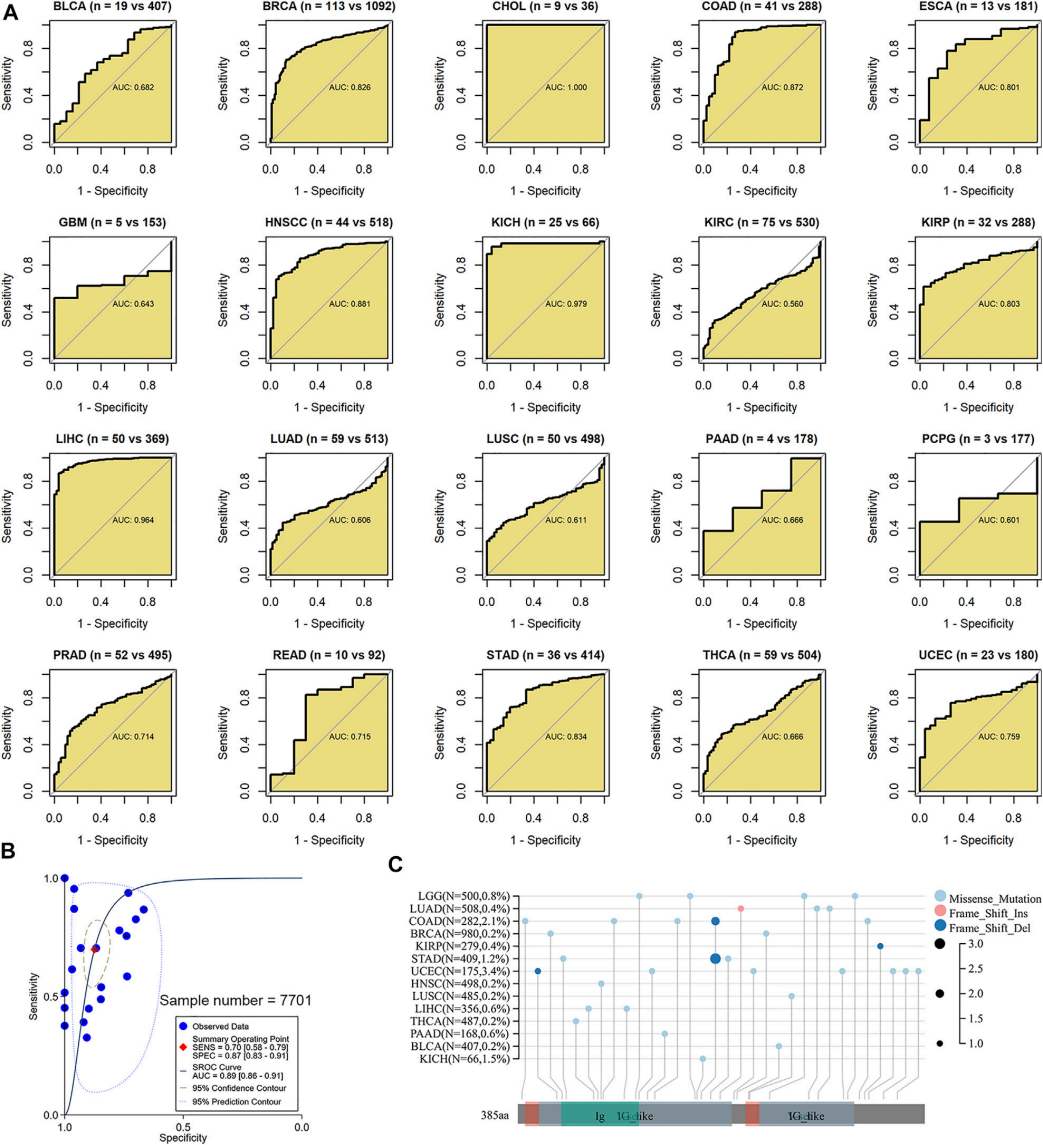
Ability of BSG expression to distinguish these cancer tissues from their normal tissues and a landscape of BSG mutations in pan-cancer. **(A)** In receiver operating characteristic curves, BSG demonstrated pronounced effects in identifying cancer tissues from their control counterparts based on the area under the curve (AUC). The two values (e.g., “n = 19 vs. 407” for BLCA) in parentheses represent the numbers of controls and cancers, respectively. **(B)** In the summary receiver operating characteristic curve, the BSG expression can accurately identify the 20 types of cancers samples from their controls. **(C)** Missense mutation is the most common alternation for BSG in pan-cancer; the panel plot was obtained from sangerBox 3.0; Del, deletion; Ins, insertion. BLCA, bladder cancer; BRCA, invasive breast carcinoma; CHOL, cholangiocarcinoma; COAD, colon adenocarcinoma; ESCA, esophageal carcinoma; GBM, glioblastoma multiforme; HNSCC, head and neck squamous cell carcinoma; KICH, kidney chromophobe; KIRC, kidney renal clear cell carcinoma; KIRP, kidney renal papillary cell carcinoma; LIHC, liver hepatocellular carcinoma; LUAD, lung adenocarcinoma; LUSC, lung squamous cell carcinoma; PAAD, pancreatic adenocarcinoma; PCPG, pheochromocytoma and paraganglioma; PRAD, prostate adenocarcinoma; READ, rectum adenocarcinoma; STAD, stomach adenocarcinoma; THCA, thyroid carcinoma; UCEC, uterine corpus endometrial carcinoma; LGG, brain lower grade glioma.

It is of great significance for the clinical management of cancers to be timely for determining the cancer status of patients. In 12 of 20 cancers in this study, *BSG* demonstrated pronounced effects in identifying cancer tissues from their control counterparts (AUC >0.7; Figure 6A). Especially in CHOL, KICH, and LIHC, the AUC values were more than 0.95 (Figure 6A), suggesting that *BSG* expression made it feasible to distinguish these cancers and implying the significant potential of *BSG* expression to predict these cancers. Moreover, in an overview of the 20 cancers, *BSG* expression also had the potential to distinguish the cancer patients from normal individuals (AUC = 0.89 [0.86–0.91]; Figure 6B). Thus, *BSG* may serve as a marker for predicting the disease status of certain cancers.

### The Landscape of *BSG* Mutations and the Expression Correlation of *BSG* With DNMTs, MMRs, and Immune Microenvironment

**FIGURE 7:**
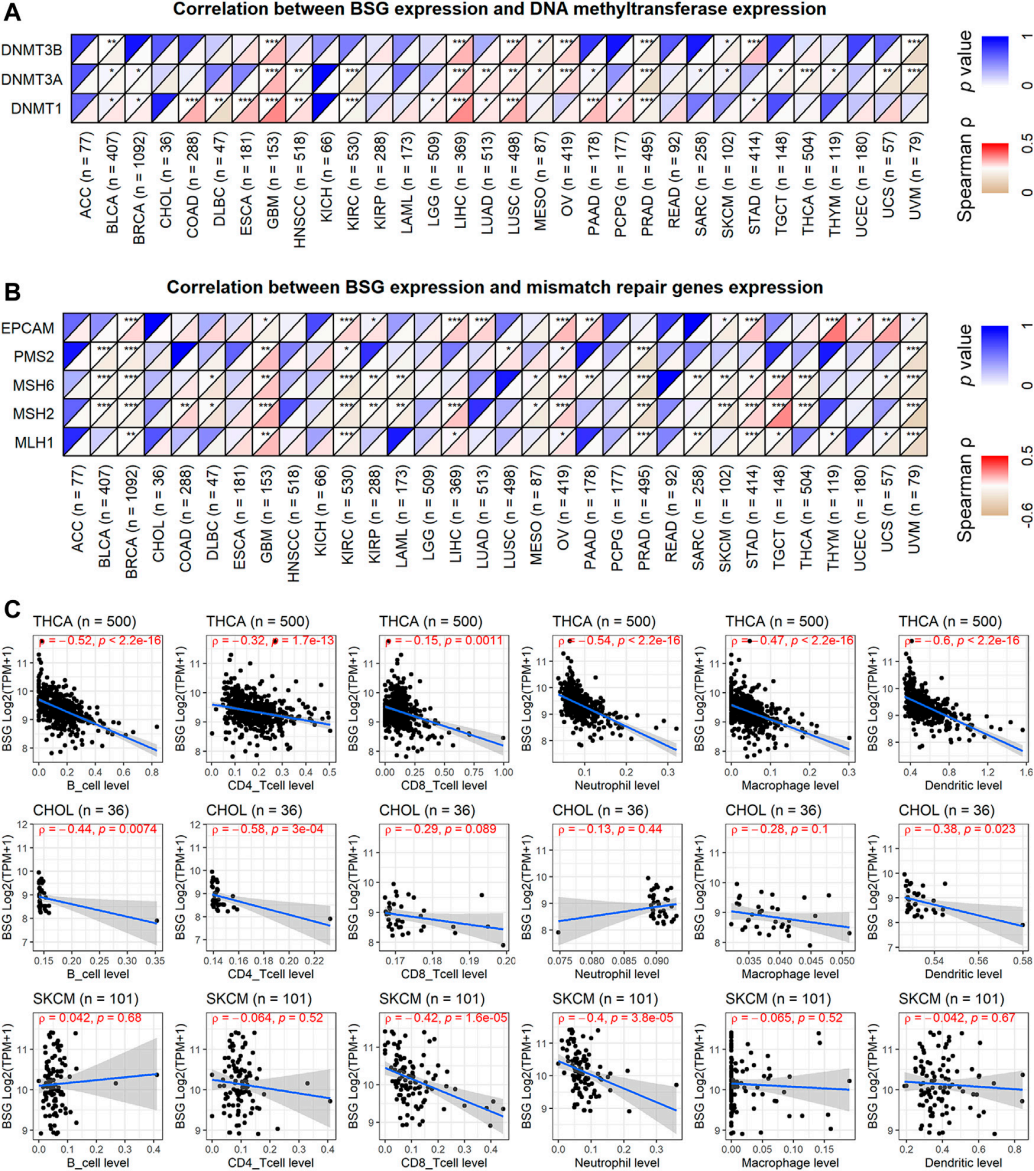
Correlations of BSG expression with DNA methylation, mismatch repair genes expression, and infiltration levels of all the six immune cells. **(A)** BSG expression was positively correlated with the three DNA methyltransferases. **(B)** BSG expression is significantly related to the five mismatch repair genes in multiple cancers. **(C)** BSG expression is negatively associated with nearly all six types of immune cells in the three cancers. The Spearman correlation coefficient follows the letter “ρ.” In **(A–C)**, the value in parentheses (e.g., “n = 77” for ACC) represents the number of samples. ACC, adrenocortical carcinoma; BLCA, bladder cancer; BRCA, invasive breast carcinoma; CHOL, cholangiocarcinoma; COAD, colon adenocarcinoma; DLBC, lymphoid neoplasm diffuse large B-cell lymphoma; ESCA, esophageal carcinoma; GBM, glioblastoma multiforme; HNSC, head and neck squamous cell carcinoma; KICH, kidney chromophobe; KIRC, kidney renal clear cell carcinoma; KIRP, kidney renal papillary cell carcinoma; LAML, acute myeloid leukemia; LGG, brain lower grade glioma; LIHC, liver hepatocellular carcinoma; LUAD, lung adenocarcinoma; LUSC, lung squamous cell carcinoma; MESO, mesothelioma; OV, ovarian serous cystadenocarcinoma; PAAD, pancreatic adenocarcinoma; PCPG, pheochromocytoma and paraganglioma; PRAD, prostate adenocarcinoma; READ, rectum adenocarcinoma; SARC, sarcoma; SKCM, skin cutaneous melanoma; STAD, stomach adenocarcinoma; TGCT, testicular germ cell tumors; THCA, thyroid carcinoma; THYM, thymoma; UCEC, uterine corpus endometrial carcinoma; UCS, uterine carcinosarcoma; UVM, uveal melanoma.

In the landscape of *BSG* mutation, missense mutation was the most common alternation, and UCEC possessed the most significant number (3.4%) of mutation (Figure 6C). The expression of three DNMTs (DNMT1, DNMT3A, and DNMT3B) was positively correlated with *BSG*, especially in GBM, LIHC, and LUSC (Figure 7A). The four proteins encoded by MLH1, MSH2, MSH6, and PMS2 have prominent roles in the MMR procession. The four genes and another MMR gene, EPCAM, are the characteristics of Lynch syndrome (a cancer-prone syndrome) (Baretti and Le 2018; Latham et al., 2019). Thus, the five MMRs (MLH1, MSH2, MSH6, PMS2, and EPCAM) were investigated in cancer-related studies (Deshpande et al., 2020; Liu et al., 2020), which were also selected in our research. As a result, *BSG* expression showed that it was significantly related to the five MMRs in multiple cancers, especially in UVM and GBM (Figure 7B), implying that *BSG* may affect the expression of MMRs in certain cancers, which needs experimental validation.

**FIGURE 8:**
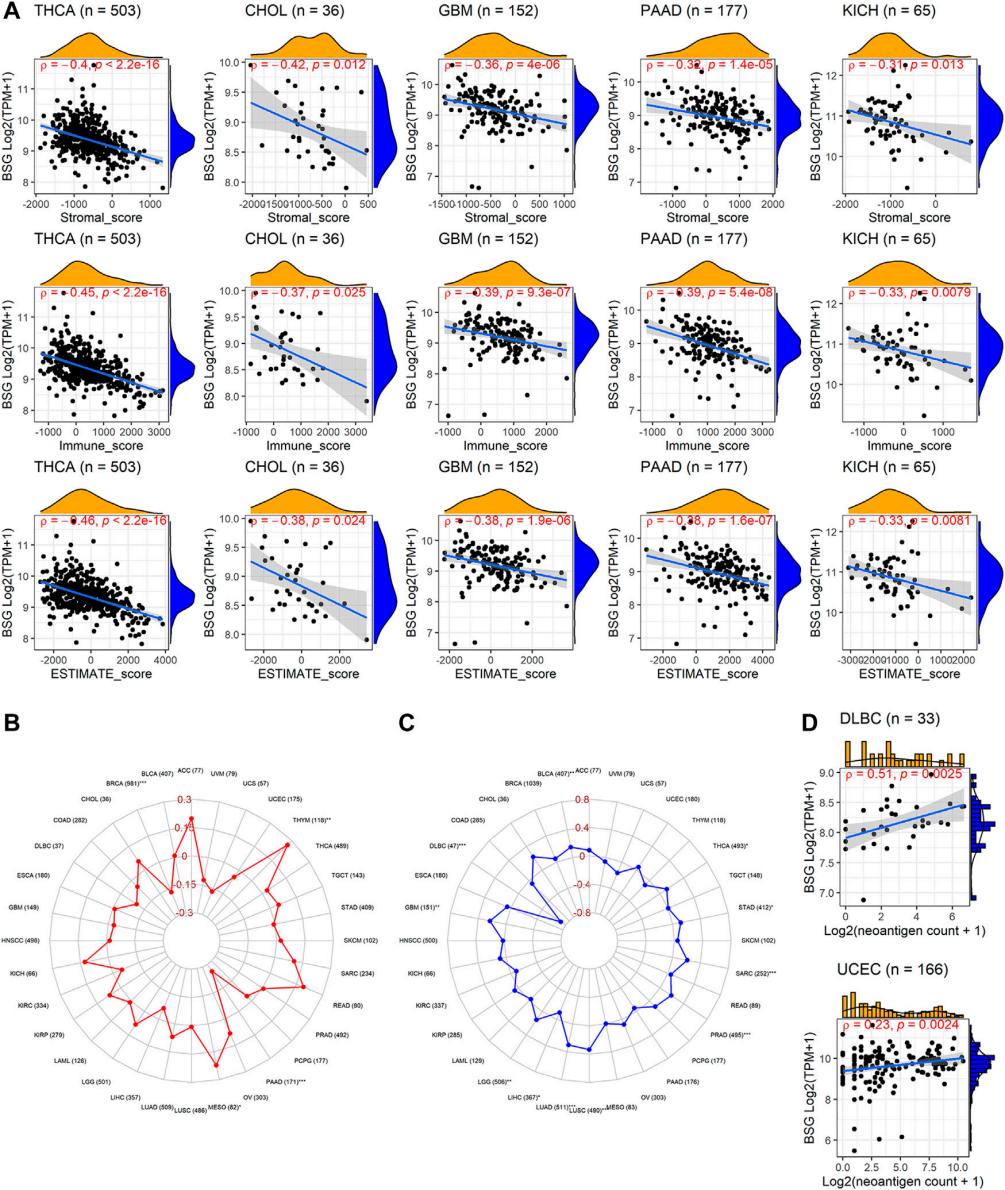
Relevance of BSG expression with immune microenvironment scores, tumor mutational burden, microsatellite instability, and neoantigen. **(A)** BSG expression was positively correlated with all five cancers’ immune, stromal, and estimate scores. **(B–C)** BSG expression demonstrates distinct (positive or negative) correlation with tumor mutational burden and microsatellite in various cancers. **(D)** BSG expression is negatively associated with the count of neoantigens in specific cancers. The Spearman correlation coefficient follows the letter “ρ.” In **(A,C)**, the value in parentheses (e.g., “n = 503” for THCA in A, and “77” for AA in **(B)**) represents the number of samples. ACC, adrenocortical carcinoma; BLCA, bladder cancer; BRCA, invasive breast carcinoma; CHOL, cholangiocarcinoma; COAD, colon adenocarcinoma; DLBC, lymphoid neoplasm diffuse large B-cell lymphoma; ESCA, esophageal carcinoma; GBM, glioblastoma multiforme; HNSCC, head and neck squamous cell carcinoma; KICH, kidney chromophobe; KIRC, kidney renal clear cell carcinoma; KIRP, kidney renal papillary cell carcinoma; LAML, acute myeloid leukemia; LGG, brain lower grade glioma; LIHC, liver hepatocellular carcinoma; LUAD, lung adenocarcinoma; LUSC, lung squamous cell carcinoma; MESO, mesothelioma; OV, ovarian serous cystadenocarcinoma; PAAD, pancreatic adenocarcinoma; PCPG, pheochromocytoma and paraganglioma; PRAD, prostate adenocarcinoma; READ, rectum adenocarcinoma; SARC, sarcoma; SKCM, skin cutaneous melanoma; STAD, stomach adenocarcinoma; TGCT, testicular germ cell tumors; THCA, thyroid carcinoma; THYM, thymoma; UCEC, uterine corpus endometrial carcinoma; UCS, uterine carcinosarcoma; UVM, uveal melanoma.

In the aspect of immune infiltration levels, *BSG* expression showed a negative correlation with nearly all six types of immune cells (B cells, CD4+ T cells, CD8+ T cells, neutrophil, macrophage, and dendritic cells) in THCA, CHOL, and SKCM (*p* < 0.05, Figure 7C) rather than other cancers (Supplementary Figure S5). For nine kinds of cancers (BLCA, BRCA, KICH, LIHC, LUAD, LUSC, PRAD, THCA, and UCEC), both low infiltration levels of at least one of six types of immune cells and upregulated *BSG* expression were detected in cancer tissues (Supplementary Table S6), suggesting that the less infiltration of specific immune cells may be due to high expression of *BSG*. Notably, decreasing levels of all six types of cells and highly expressed *BSG* can be observed in LUSC (Supplementary Table S6), highlighting that *BSG* was likely to affect the six immune cell levels in LUSC patients. As for the ESTIMATE scores, *BSG* expression appeared negatively related to all of the immune, stromal, and estimate scores in THCA, CHOL, GBM, PAAD, and KICH (*p* < 0.05, Figure 8A). However, that was not conspicuous for other cancers (Supplementary Figure S6). In short, the biological functions of *BSG* in specific cancers may attribute to its negative correlation with the immune microenvironment.

### Correlation of *BSG* Expression With TMB, MSI, Neoantigen Count, and Immune Checkpoint Genes

TMB was a measurement of mutation levels in tumors. It was positively related to *BSG* expression in MESO and THYM and negatively correlated in BRCA and PAAD by the Spearman rank correlation analysis (*p* < 0.05; Figure 8B). MSI was a pattern of hypermutation caused by several mechanisms such as MMRs, and it was positively associated with *BSG* expression in BLCA, GBM, LGG, LUAD, LUSC, PRAD, SARC, STAD, and THCA (*p* < 0.05) and negatively related with DLBC and LIHC (*p* < 0.05) (Figure 8C). Higher TMB and MSI levels may contribute to elevated neoantigens (particularly tumor antigens) and thus trigger immune responses of the body (Jardim et al., 2021) (Schumacher, Scheper, and Kvistborg 2019). In our research, neoantigens demonstrated mild to moderate correlation with the count of neoantigens in DLBC and UCEC (*p* < 0.05, Figure 8D).

Previous studies have shown that tumor cells can suppress body immune response by motivating the expression of the immune checkpoints (Toor et al., 2020). Based on the correlation analysis, *BSG* expression was negatively (*ρ* < -0.3, *p* < 0.05) correlated with at least ten checkpoints in seven cancers (CHOL, KICH, KIRC, PAAD, THCA, THYM, and UVM) (Supplementary Figure S7).

### GSEA and Drug Susceptibility of *BSG* in Cancers

**FIGURE 9:**
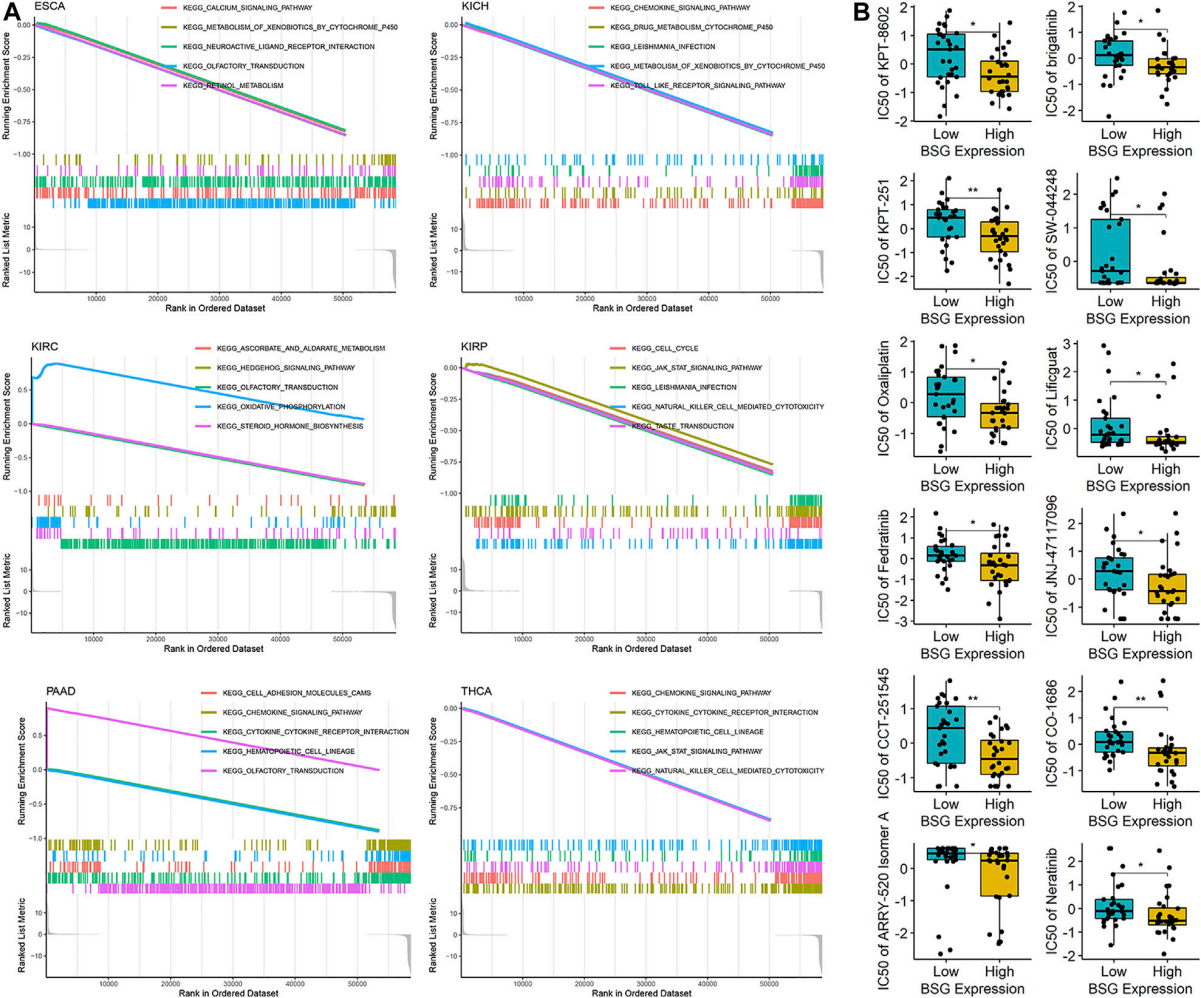
Potential mechanisms of BSG in pan-cancer and the drug sensitivity for the gene. **(A)** Gene set enrichment analysis suggests that BSG is identified to involve no fewer than five signaling pathways. **(B)** Drug sensitivity of BSG; the high-BSG expression group represented low-IC50 (half maximal inhibitory concentration) for the 12 drugs shown in the panel, implying cancer cells with a high-BSG expression may be sensitive to these drugs. ^*^ *p* < 0.05, ^**^ *p* < 0.01; *p* values are based on the one-sided Wilcoxon rank-sum test.

The study investigated potential mechanisms of *BSG* in 32 cancers based on KEGG signaling pathways by GSEA. *BSG* was likely an essential factor in the occurrence and development of ESCA, KICH, KIRC, KIRP, PAAD, and THCA by complex mechanisms as it was identified to involve no fewer than five signaling pathways (Figure 9A). Totally, 39 KEGG signaling pathways were found for *BSG* in 32 cancers in this study (Supplementary Table S7). In at least five cancers, *BSG* was shown closely related to three pathways—olfactory transduction, metabolism of xenobiotics by cytochrome P450, and hematopoietic cell lineage (Supplementary Table S7) indicating the gene may play its roles in multiple human cancers. Moreover, some pathways such as drug metabolism cytochrome P450 and JAK-STAT signaling pathways were also found in the GSEA of *BSG* (Supplementary Table S7). For the top seven essential signaling pathways (olfactory transduction, etc.) presenting statistical significance in at least four cancers, correlations of their leading-edge genes with *BSG* expression were explored. For instance, for the olfactory transduction pathway demonstrating statistical significance in 15 cancers (KIRC, etc.), 21 leading-edge genes were found in the pathway. The relationship between them with *BSG* expression was explored in the 15 cancers. As a result, the significant (positive or negative) relevance of *BSG* expression with the 21 leading-edge genes was identified in certain types of the 15 cancers based on the Spearman rank correlation analysis (*p* < 0.05; Supplementary Figure S8A). A similar analysis was carried out for the other six essential pathways—hematopoietic cell lineage, metabolism of xenobiotics by cytochrome p450, drug metabolism cytochrome p450, JAK-STAT signaling pathway, *Leishmania* infection, and pentose and glucuronate interconversions; as a result, *BSG* expression primarily showed negative correlations with leading-edge genes of the six signaling pathways (*p* < 0.05; Supplementary Figures S8B–G), indicating the roles of *BSG* played in specific cancers may be due to the disorder of these pathways.

Furthermore, by using CellMiner, this research detected the sensitivity of *BSG* to 57 drugs approved by the Food and Drug Administration of America or validated by clinical tests. Notably, high-*BSG* expression was sensitive to 12 (e.g., KPT-8602 and brigatinib) of the 57 drugs (Figure 9B), suggesting the potential of these drugs in treating cancer patients with elevated *BSG* expression.

## Discussion

Nowadays, COVID-19 has been a severe global public health problem since December 2019 (Li Q et al., 2020). Because of the susceptibility of cancer patients to the infection of SARS-CoV-2 and the occurrence of severe complications in the cancer patients, the prevalence of COVID-19 poses significant challenges to the management of cancer patients (Liang et al., 2020; Moujaess, Kourie, and Ghosn 2020). *BSG* is a vital entrance of SARS-CoV-2 into human cells and is upregulated in both COVID-19 patients and tumor patients (Monteiro et al., 2014; Xin et al., 2016; Bergsneider et al., 2021). Thus, there may be a linkage between COVID-19 and cancers (Varadarajan et al., 2020). However, although the other essential receptor for SARS-CoV-2 invading normal cells—angiotensin-converting enzyme 2—has received extensive attention in tumor-related research, effort on *BSG* is relatively limited, which needs to be further analyzed.

To promote the understanding of *BSG* in multiple human cancers, a systematic analysis of COVID-19-related *BSG* in pan-cancer was performed based on 19,020 samples in this study. Upregulated and downregulated *BSG* expression was observed in 14 cancers (BRCA, etc.) and two cancers (COAD and READ), respectively. The increasing BSG protein levels were also observed in kidney cancer (for KICH and KIRP), LIHC, THCA, and UCEC. *BSG* served as a risk role for the prognosis of patients with BRCA, LGG, LUAD, PRAD, and UCS in at least one of OS, DSS, DFI, and PFI, while it also represented a protective factor in the prognosis of individuals with KIRP, PAAD, and THYM in OS, DSS, or PFI. *BSG* expression showed a pronounced effect in identifying multiple cancers (e.g., CHOL) from their control samples. Moreover, the associations of *BSG* expression with TMB, MSI, neoantigen, and immune checkpoints suggested the potential of *BSG* as an exciting target for cancer treatment.

Generally, overexpression of *BSG* is common in many cancers. Our study revealed differences in the *BSG* expression levels in various human organs and cancer cells. In terms of tissues, previous reports have identified differently expressed *BSG* in a few cancers, such as melanoma (Kim et al., 2021; Lu et al., 2021) and LIHC (Wang SJ et al., 2020). In our study, compared with normal tissues, *BSG* mRNA was highly expressed in cancers containing BLCA, BRCA, CHOL, ESCA, HNSCC, KICH, KIRP, LIHC, LUAD, and LUSC, PRAD, STAD, THCA, and UCEC, while it was lowly expressed in COAD and READ. In most of these cancers, increasing (exceptionally, decreasing for COAD) *BSG* expression had been detected before (Han et al., 2010; Xue, Lu, and Sun 2011; Zhong et al., 2012; Liu et al., 2013; Xu et al., 2013; Huang et al., 2019; Yu et al., 2019; He et al., 2021). These reports mainly focused on protein levels of BSG, which were consistent with the mRNA results in our study. Indeed, we also validated elevated BSG protein levels in kidney cancer (for KICH and KIRP), LIHC, THCA, and UCEC. Collectively, differential (mainly increasing) *BSG* expression was observed in various cancers.

*BSG* is not only differently expressed in most tumors but represents different prognosis results and identifies cancer status in some cancers. Previously, Suzuki et al. (2021) revealed CD147 (alias *BSG*) as an indicator of the poor prognosis of patients with hypopharyngeal cancer. Miyazaki et al. (2020) reported that the upregulated EMMPRIN (another name for *BSG*) expression was also associated with a poor external auditory canal carcinoma prognosis. Our study discussed several types of survival information for further exploring the roles *BSG* played in the prognosis of multiple human cancers. In OS, DSS, DFI, and/or PFI, overexpressed *BSG* expression was associated with an unfavorable prognosis in patients with BRCA, LGG, LUAD, PRAD, and UCS. At the same time, it showed a favorable prognosis for patients with KIRP, PAAD, and THYM. Moreover, *BSG* made it feasible to differentiate 12 cancer (particularly CHOL, KICH, and LIHC) tissues from their normal tissues, implying its potential in screening patients with these cancers from normal individuals. This finding, to our knowledge, had not been revealed before, indicating the novelty of our study. Thus, the prognosis value and identification effect of *BSG* expression in cancers were conspicuous.

*BSG* may be an important potential target for treating various cancers. Constantly, the function of immunocytes facilitates the body’s antivirus and anti-tumor biological process. Thus, decreasing levels of immune cells will cause deterioration in COVID-19 patients and cancer patients. Indeed, previous research suggests lymphocyte reduction is an indicator of COVID-19 patients and various cancer patients (Li M et al., 2020; Tan et al., 2020). Furthermore, immunosuppression and weakened immune system were significant causes of severe disease course and high mortality in cancer patients with COVID-19 (Xia and Dubrovska 2020). Interestingly, based on this study and previous reports, *BSG* played multiple biological roles: 1) one of the significant known receptors of SARS-CoV-2 (Wang K et al., 2020; Fenizia et al., 2021; Geng et al., 2021; Behl et al., 2022); 2) a factor negatively related to the infiltration levels of innate and adaptive immune cells; 3) a factor conversely correlated with immune matrix levels, and 4) a prognostic indicator for multiple cancers. These results supported each other and suggested that *BSG* may be involved in the negative regulation of immune response. In addition, this study not only found that *BSG* expression was related to the DNMT expression, MMRs expression, TMB level of cancer (KICH, etc.) patients, and MSI level of cancer (BLCA, etc.) patients but also found that the expression level of *BSG* was related to neoantigens (for DLBC and UCEC) and the expression levels of multiple immune checkpoints. These results may promote the understanding of the potential role of *BSG* in tumor immunology.

This study also preliminarily explored the potential molecular mechanism of *BSG* in various human cancers and discussed the sensitivity of some drugs to *BSG*. In the GSEA, *BSG* was closely related to olfactory transduction, xenobiotics metabolism by cytochrome P450, hematopoietic cell lineage, drug cytochrome metabolism P450, JAK-STAT signaling pathways, *Leishmania* infection, and pentose and glucuronate interconversions. Indeed, previous single tumor studies had demonstrated these findings, such as Knutti et al. (2019), revealing that *BSG* affects the malignancy of BRCA cells by affecting Wnt and JAK/STAT signaling pathways. Another example was that *BSG* was also considered to be a vital molecule causing multiple cancer resistance (Landras and Mourah 2020); combined with the GSEA results of this study, this may be related to *BSG* participating in drug metabolism cytochrome P450 and other pathways. However, the mechanism of *BSG* in tumors was complex (e.g., 39 KEGG signaling pathways were found associated with *BSG*), which still needs further experimental studies. Based on drug sensitivity, our research also revealed that some drugs might be applied to cancer patients with high expression of *BSG*, but more effort is also needed to confirm these results further.

Some limitations to this study should be of concern. Above all, we failed to collect samples from patients diagnosed with both COVID-19 and cancer, and thus, we could not directly identify whether these patients with differently expressed *BSG* expression had poor prognosis in our study. Specimens utilized for exploring BSG protein levels are relatively limited. The potential mechanisms of *BSG* in multiple cancers require experimental validation.

This study revealed that COVID-19-related *BSG* was differently expressed in various human cancers. The gene is a prognostic marker for various cancers and has the potential to screen several cancers. In short, *BSG* may be a novel target for cancer treatment and identification.

## Data Availability

The datasets presented in this study can be found in online repositories. The names of the repository/repositories and accession number(s) can be found in the article/Supplementary Material.
