# Supplementary figures and images for: Pan-Cancer Analysis, Reveals COVID-19-Related BSG as a Novel Marker for Treatment and Identification of Multiple Human Cancers
Source: Front Cell Dev Biol. 2022 May 13;10:876180. doi: 10.3389/fcell.2022.876180 (PMC9136262; doi:10.3389/fcell.2022.876180)

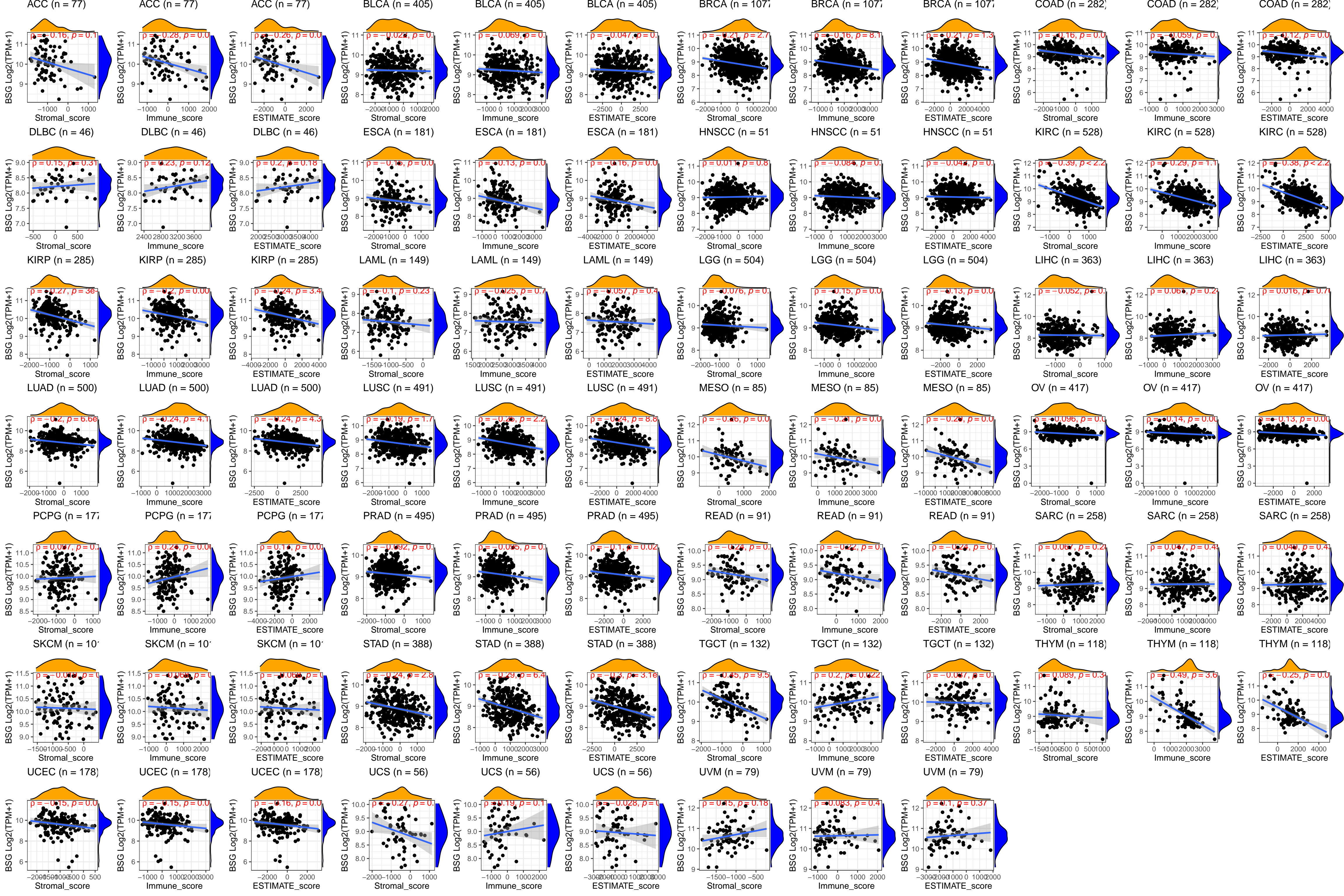

Supplement: Supplementary file 1 [file DataSheet2.PDF]

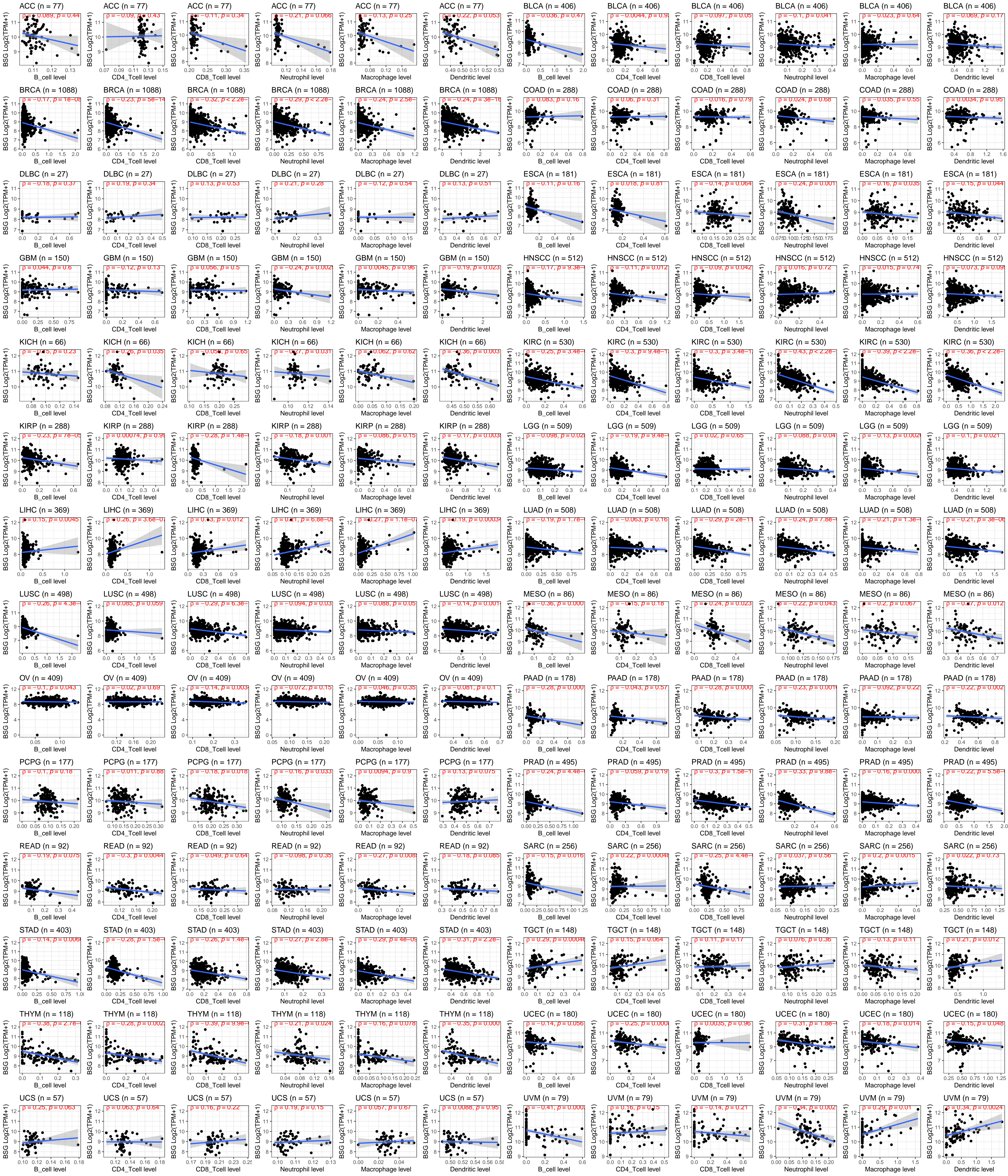

Supplement: Supplementary file 3 [file Image5.pdf]

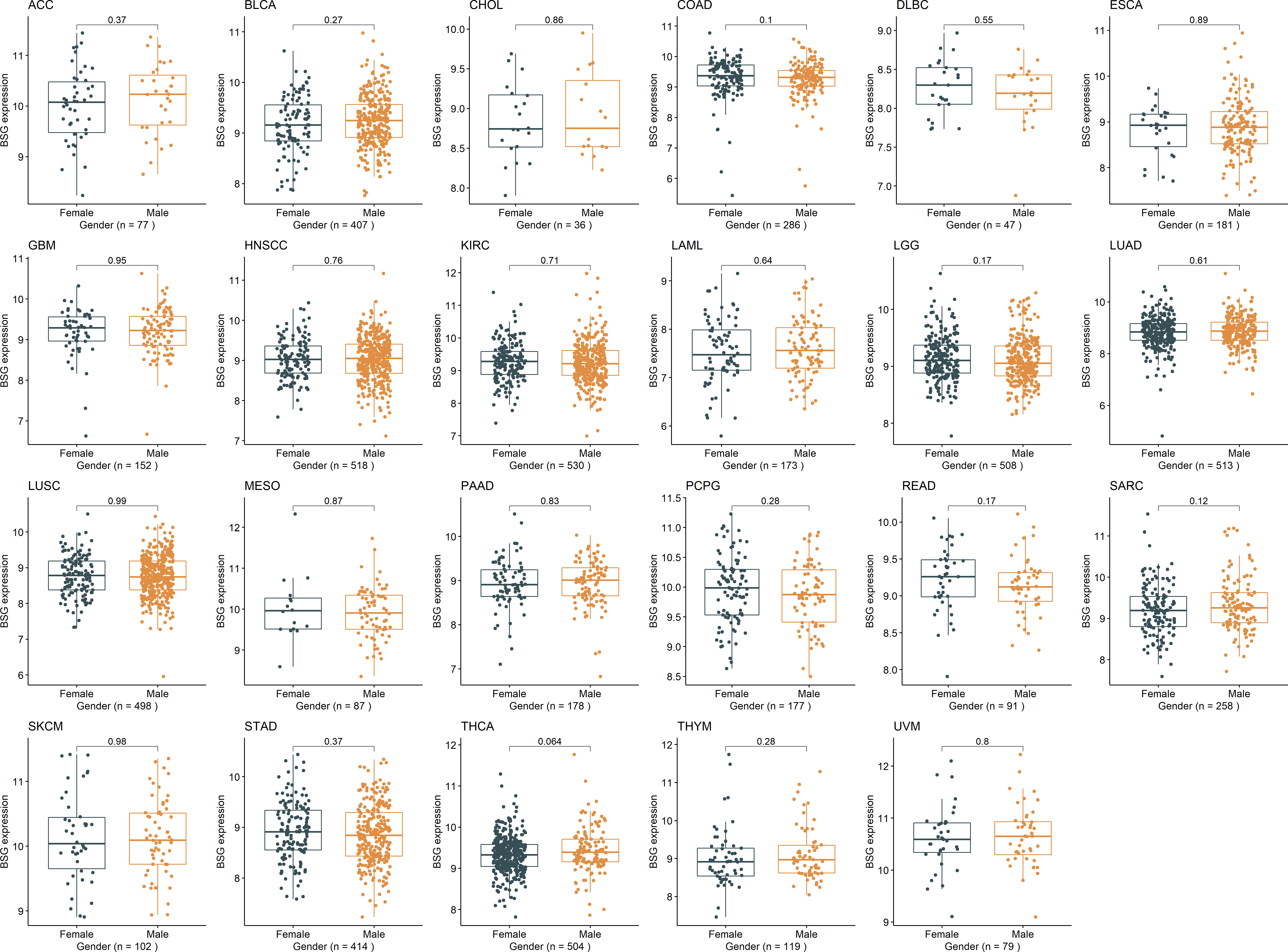

Supplement: Supplementary file 4 [file Image3.tif]

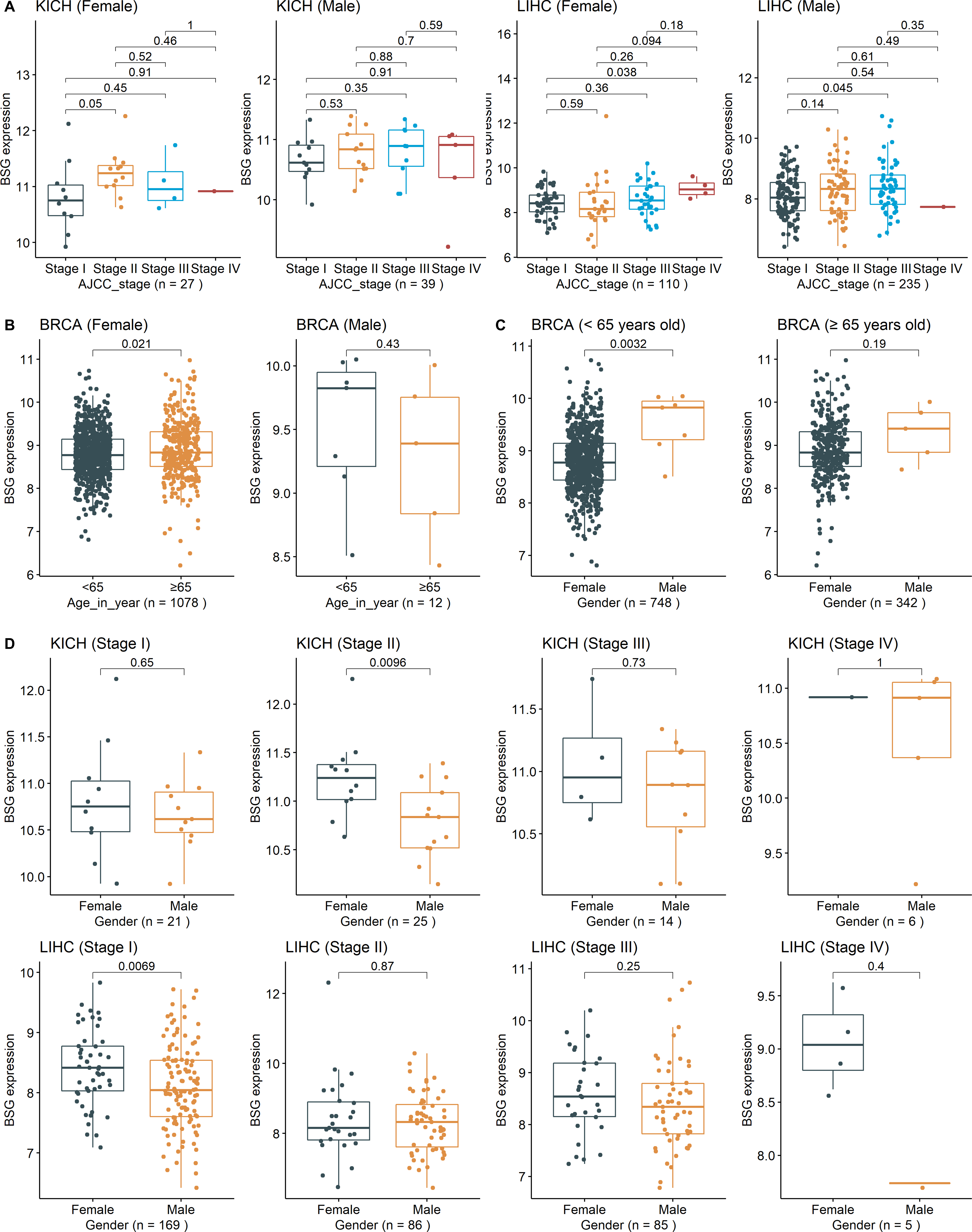

Supplement: Supplementary file 5 [file Image4.tif]

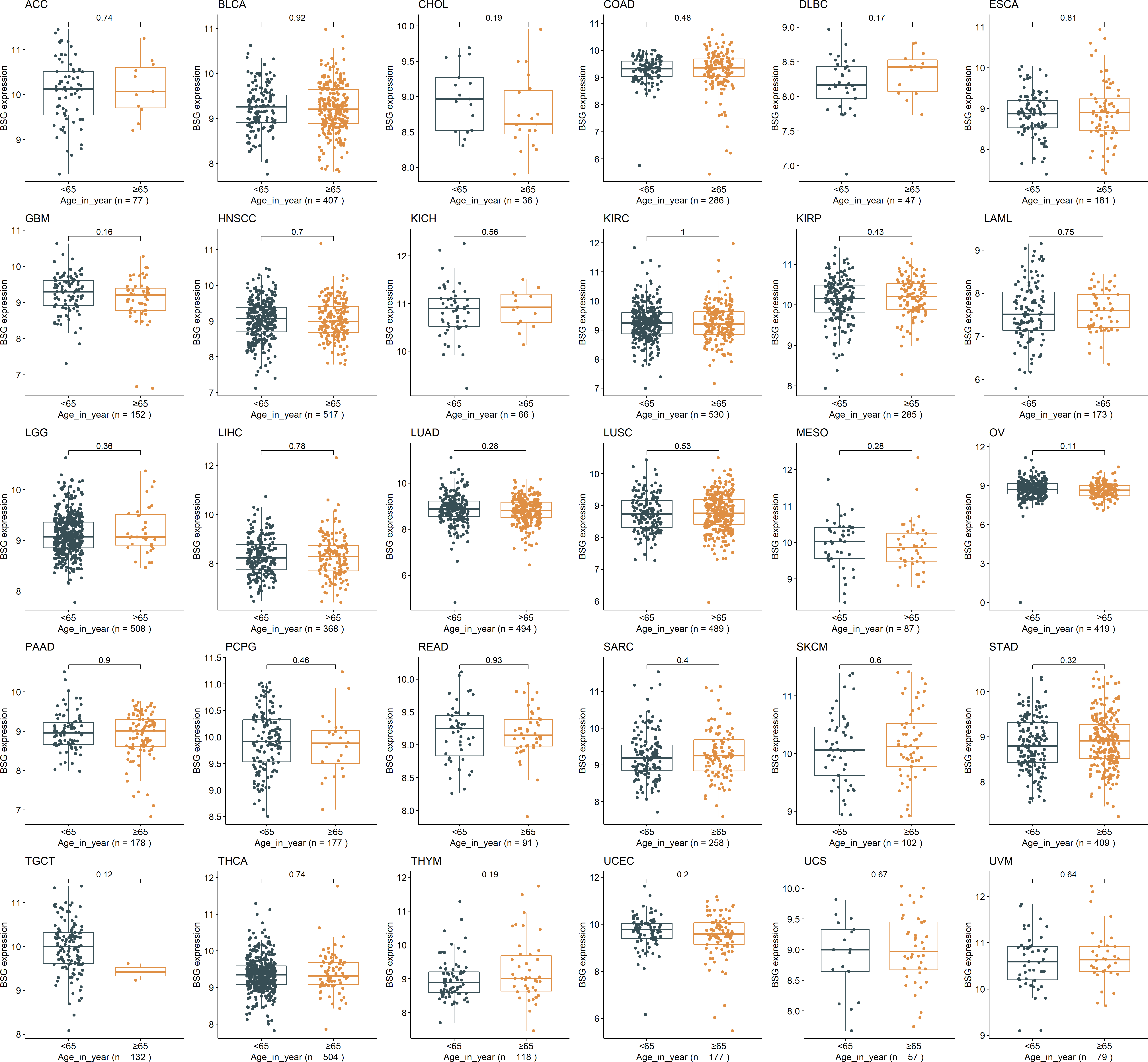

Supplement: Supplementary file 7 [file Image2.tif]

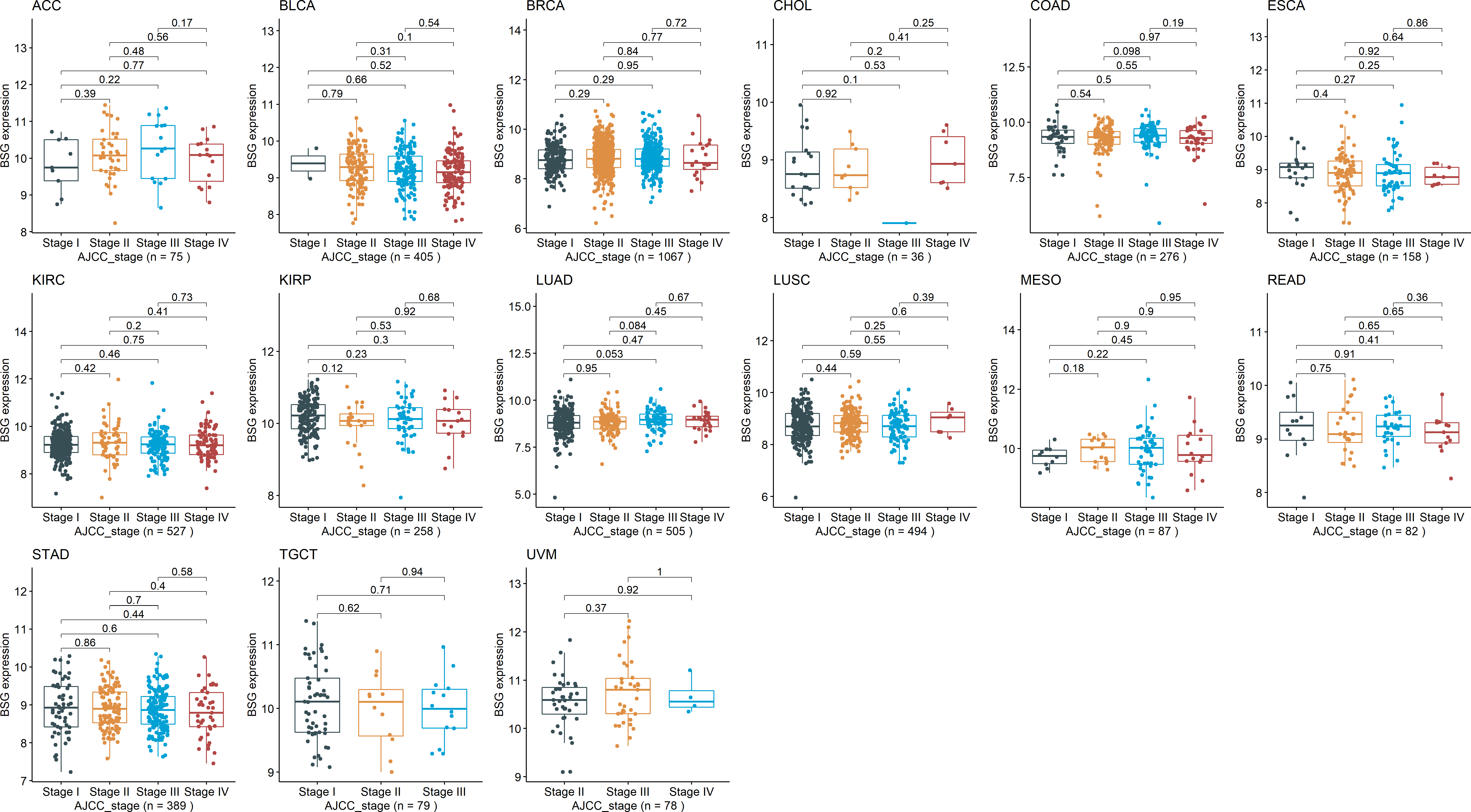

Supplement: Supplementary file 8 [file Image1.tif]

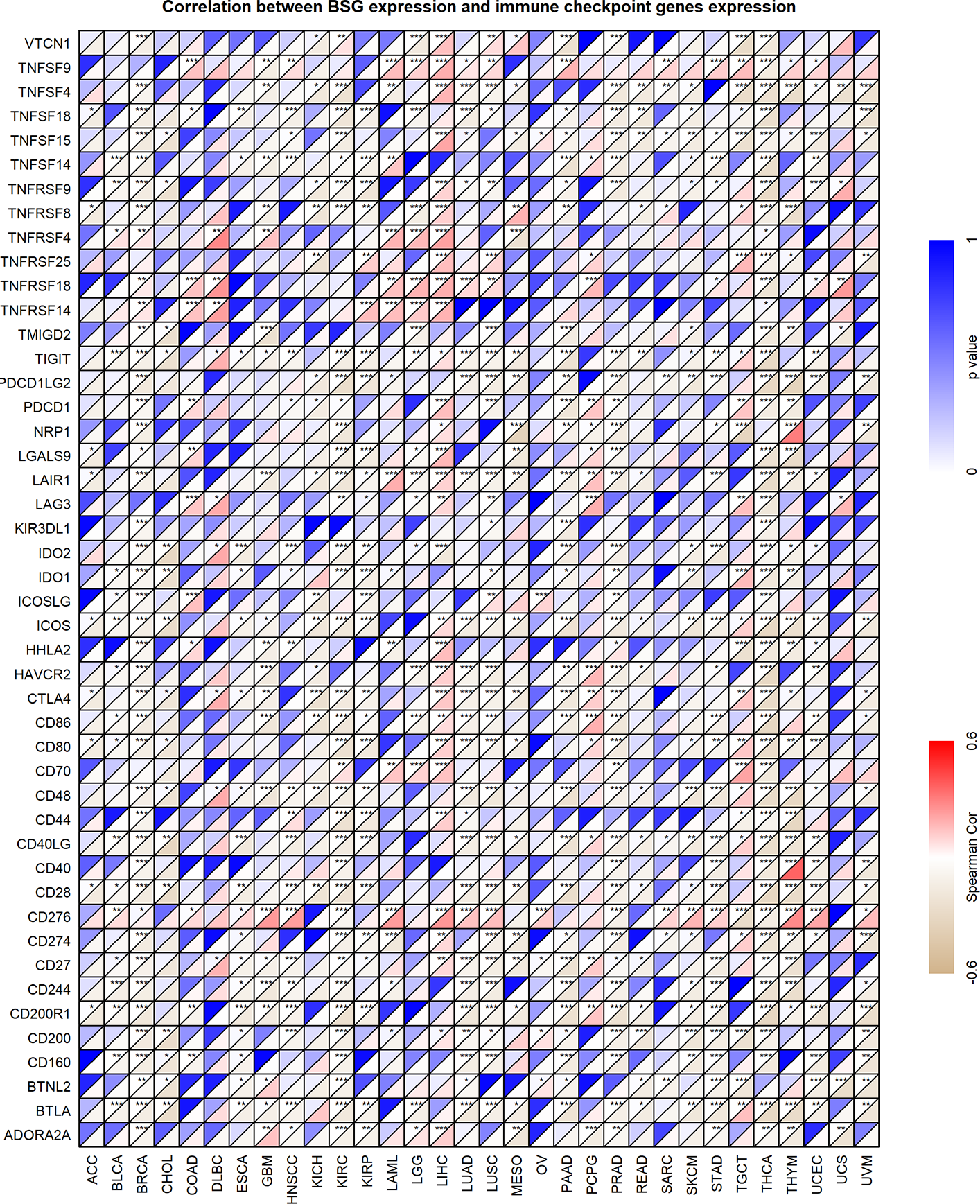

Supplement: Supplementary file 10 [file Image7.tif]

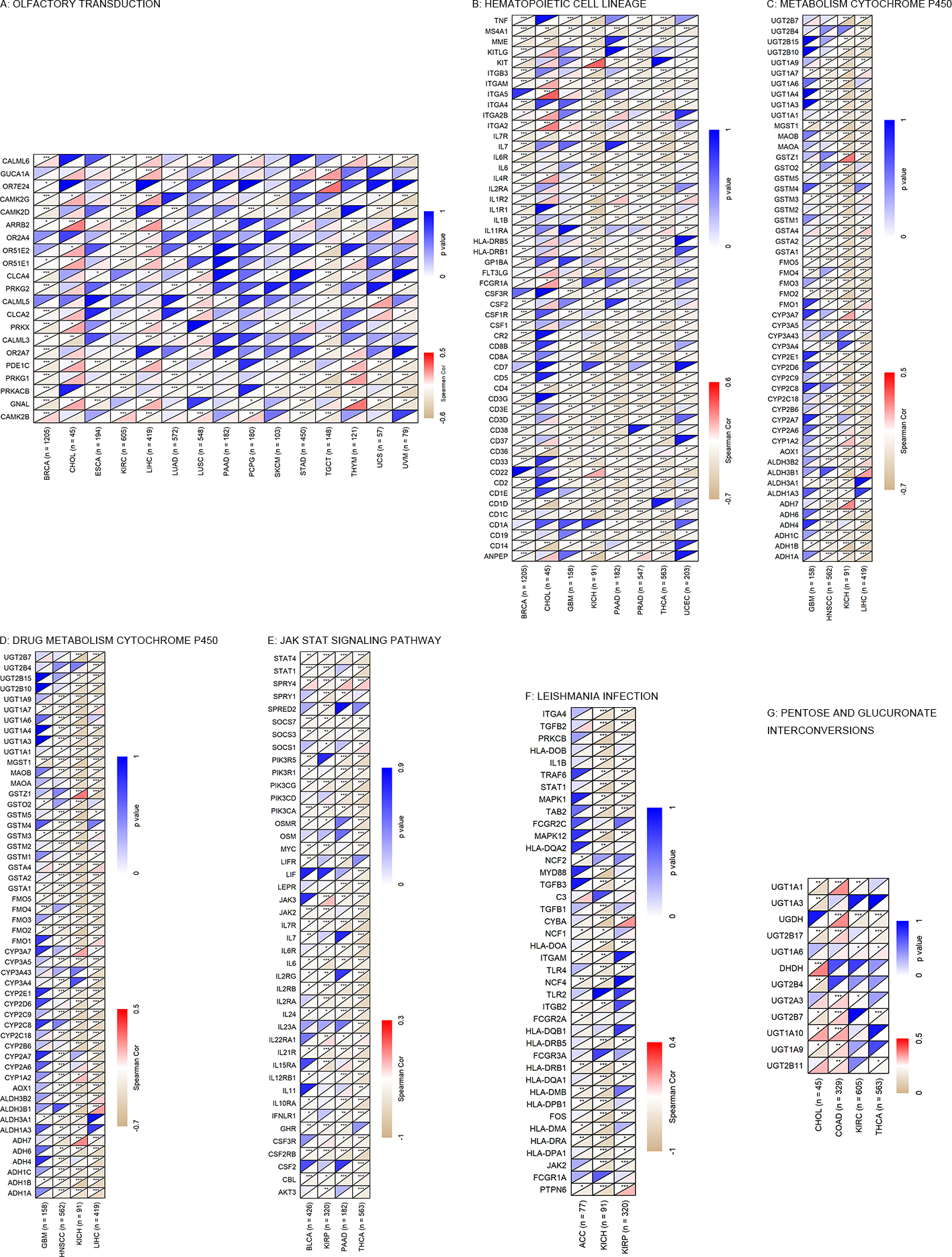

Supplement: Supplementary file 18 [file Image8.tif]
