# Supplementary material for: Pan-Cancer Analysis, Reveals COVID-19-Related BSG as a Novel Marker for Treatment and Identification of Multiple Human Cancers
Source: Front Cell Dev Biol. 2022 May 13;10:876180. doi: 10.3389/fcell.2022.876180 (PMC9136262; doi:10.3389/fcell.2022.876180)
Supplement: Supplementary file 12 [file Table2.docx]

**Supplementary Table S2.** Samples included for the validation of BSG expression at the protein level.

| Patient ID^a^ | For cancer | Age | Gender | Tissue | Group | Score | | |
| --- | --- | --- | --- | --- | --- | --- | --- | --- |
|  |  |  |  |  |  | Intensity | Quantity | Total IHC |
| 2047 | BLCA | 55 | Female | Bladder | Normal | 0 | 0 | 0 |
| 2053 | BLCA | 51 | Male | Bladder | Normal | 0 | 0 | 0 |
| 31 | BLCA | 78 | Female | Bladder | Tumor | 0 | 0 | 0 |
| 239 | BLCA | 71 | Male | Bladder | Tumor | 2 | 2 | 4 |
| 267 | BLCA | 84 | Female | Bladder | Tumor | 3 | 3 | 9 |
| 389 | BLCA | 89 | Female | Bladder | Tumor | 2 | 3 | 6 |
| 519 | BLCA | 56 | Male | Bladder | Tumor | 2 | 3 | 6 |
| 792 | BLCA | 86 | Male | Bladder | Tumor | 2 | 3 | 6 |
| 1096 | BLCA | 84 | Male | Bladder | Tumor | 2 | 3 | 6 |
| 1157 | BLCA | 54 | Male | Bladder | Tumor | 3 | 3 | 9 |
| 1204 | BLCA | 73 | Female | Bladder | Tumor | 0 | 0 | 0 |
| 2042 | BRCA | 75 | Female | Breast | Normal | 0 | 0 | 0 |
| 2259 | BRCA | 23 | Female | Breast | Normal | 0 | 0 | 0 |
| 159 | BRCA | 68 | Female | Breast | Tumor | 0 | 0 | 0 |
| 261 | BRCA | 68 | Female | Breast | Tumor | 0 | 0 | 0 |
| 278 | BRCA | 45 | Female | Breast | Tumor | 1 | 2 | 2 |
| 315 | BRCA | 50 | Female | Breast | Tumor | 2 | 3 | 6 |
| 609 | BRCA | 58 | Female | Breast | Tumor | 0 | 0 | 0 |
| 614 | BRCA | 85 | Female | Breast | Tumor | 1 | 2 | 2 |
| 659 | BRCA | 69 | Female | Breast | Tumor | 0 | 0 | 0 |
| 768 | BRCA | 64 | Female | Breast | Tumor | 0 | 0 | 0 |
| 830 | BRCA | 24 | Female | Breast | Tumor | 0 | 0 | 0 |
| 1044 | BRCA | 57 | Female | Breast | Tumor | 0 | 0 | 0 |
| 1155 | BRCA | 56 | Female | Breast | Tumor | 0 | 0 | 0 |
| 1271 | BRCA | 50 | Female | Breast | Tumor | 2 | 3 | 6 |
| 1859 | Kidney | 61 | Male | Kidney | Normal | 0 | 0 | 0 |
| 2530 | Kidney | 41 | Female | Kidney | Normal | 0 | 0 | 0 |
| 3229 | Kidney | 59 | Male | Kidney | Normal | 0 | 0 | 0 |
| 1831 | Kidney | 77 | Male | Kidney | Tumor | 1 | 3 | 3 |
| 1901 | Kidney | 69 | Female | Kidney | Tumor | 1 | 1 | 1 |
| 1969 | Kidney | 63 | Male | Kidney | Tumor | 1 | 2 | 2 |
| 2210 | Kidney | 64 | Female | Kidney | Tumor | 2 | 3 | 6 |
| 2261 | Kidney | 59 | Male | Kidney | Tumor | 2 | 3 | 6 |
| 2477 | Kidney | 80 | Male | Kidney | Tumor | 1 | 3 | 3 |
| 2545 | Kidney | 72 | Female | Kidney | Tumor | 2 | 3 | 6 |
| 2655 | Kidney | 56 | Male | Kidney | Tumor | 1 | 2 | 2 |
| 3039 | Kidney | 67 | Female | Kidney | Tumor | 1 | 1 | 1 |
| 3156 | Kidney | 63 | Male | Kidney | Tumor | 2 | 3 | 6 |
| 3541 | Kidney | 59 | Female | Kidney | Tumor | 2 | 2 | 4 |
| 1720 | LIHC | 67 | Male | Liver | Normal | 0 | 0 | 0 |
| 3222 | LIHC | 63 | Female | Liver | Normal | 0 | 0 | 0 |
| 3402 | LIHC | 54 | Female | Liver | Normal | 0 | 0 | 0 |
| 983 | LIHC | 53 | Female | Liver | Tumor | 2 | 2 | 4 |
| 2177 | LIHC | 58 | Female | Liver | Tumor | 1 | 1 | 1 |
| 2325 | LIHC | 76 | Male | Liver | Tumor | 2 | 2 | 4 |
| 2556 | LIHC | 72 | Male | Liver | Tumor | 1 | 1 | 1 |
| 2766 | LIHC | 73 | Female | Liver | Tumor | 2 | 2 | 4 |
| 3196 | LIHC | 65 | Male | Liver | Tumor | 0 | 0 | 0 |
| 3324 | LIHC | 70 | Male | Liver | Tumor | 1 | 1 | 1 |
| 1470 | LUAD & LUSC | 65 | Male | Lung | Normal | 0 | 0 | 0 |
| 1678 | LUAD & LUSC | 57 | Female | Lung | Normal | 0 | 0 | 0 |
| 2208 | LUAD & LUSC | 67 | Female | Lung | Normal | 0 | 0 | 0 |
| 426 | LUAD | 48 | Male | Lung | Tumor | 2 | 3 | 6 |
| 970 | LUAD | 76 | Male | Lung | Tumor | 0 | 0 | 0 |
| 1249 | LUAD | 44 | Female | Lung | Tumor | 0 | 0 | 0 |
| 1303 | LUAD | 68 | Male | Lung | Tumor | 0 | 0 | 0 |
| 1327 | LUAD | 64 | Male | Lung | Tumor | 0 | 0 | 0 |
| 447 | LUSC | 62 | Male | Lung | Tumor | 0 | 0 | 0 |
| 2663 | LUSC | 74 | Male | Lung | Tumor | 1 | 1 | 1 |
| 4128 | LUSC | 66 | Female | Lung | Tumor | 2 | 1 | 2 |
| 4890 | LUSC | 60 | Male | Lung | Tumor | 2 | 2 | 4 |
| 376 | LUSC | 64 | Male | Lung | Tumor | 2 | 3 | 6 |
| 4898 | LUSC | 76 | Male | Lung | Tumor | 2 | 2 | 4 |
| 2053 | PRAD | 51 | Male | Prostate | Normal | 0 | 0 | 0 |
| 2098 | PRAD | 60 | Male | Prostate | Normal | 0 | 0 | 0 |
| 3497 | PRAD | 37 | Male | Prostate | Normal | 0 | 0 | 0 |
| 3455 | PRAD | 63 | Male | Prostate | Tumor | 3 | 1 | 3 |
| 4157 | PRAD | 64 | Male | Prostate | Tumor | 0 | 0 | 0 |
| 4163 | PRAD | 71 | Male | Prostate | Tumor | 2 | 2 | 4 |
| 4338 | PRAD | 55 | Male | Prostate | Tumor | 1 | 1 | 1 |
| 4350 | PRAD | 68 | Male | Prostate | Tumor | 1 | 1 | 1 |
| 4354 | PRAD | 68 | Male | Prostate | Tumor | 0 | 0 | 0 |
| 4356 | PRAD | 69 | Male | Prostate | Tumor | 3 | 1 | 3 |
| 4363 | PRAD | 63 | Male | Prostate | Tumor | 3 | 2 | 6 |
| 4365 | PRAD | 71 | Male | Prostate | Tumor | 0 | 0 | 0 |
| 4439 | PRAD | 42 | Male | Prostate | Tumor | 0 | 0 | 0 |
| 4521 | PRAD | 62 | Male | Prostate | Tumor | 2 | 1 | 2 |
| 4529 | PRAD | 60 | Male | Prostate | Tumor | 1 | 1 | 1 |
| 1884 | STAD | 57 | Male | Stomach | Normal | 0 | 0 | 0 |
| 1650 | STAD | 55 | Male | Stomach | Normal | 0 | 0 | 0 |
| 2105 | STAD | 62 | Male | Stomach | Tumor | 1 | 1 | 1 |
| 2326 | STAD | 65 | Female | Stomach | Tumor | 0 | 0 | 0 |
| 2378 | STAD | 59 | Male | Stomach | Tumor | 1 | 1 | 1 |
| 2959 | STAD | 59 | Female | Stomach | Tumor | 0 | 0 | 0 |
| 3044 | STAD | 48 | Male | Stomach | Tumor | 2 | 1 | 2 |
| 3055 | STAD | 77 | Male | Stomach | Tumor | 1 | 1 | 1 |
| 3492 | STAD | 55 | Male | Stomach | Tumor | 0 | 0 | 0 |
| 3526 | STAD | 60 | Female | Stomach | Tumor | 1 | 2 | 2 |
| 2072 | THCA | 61 | Male | Thyroid gland | Normal | 0 | 0 | 0 |
| 2146 | THCA | 22 | Female | Thyroid gland | Normal | 0 | 0 | 0 |
| 2219 | THCA | 33 | Female | Thyroid gland | Normal | 0 | 0 | 0 |
| 306 | THCA | 76 | Male | Thyroid gland | Tumor | 2 | 2 | 4 |
| 348 | THCA | 87 | Male | Thyroid gland | Tumor | 3 | 2 | 6 |
| 923 | THCA | 71 | Female | Thyroid gland | Tumor | 2 | 3 | 6 |
| 985 | THCA | 71 | Female | Thyroid gland | Tumor | 1 | 1 | 1 |
| 974 | UCEC | 37 | Female | Endometrium | Normal | 0 | 0 | 0 |
| 1185 | UCEC | 61 | Female | Endometrium | Normal | 0 | 0 | 0 |
| 1317 | UCEC | 36 | Female | Endometrium | Normal | 0 | 0 | 0 |
| 1328 | UCEC | 60 | Female | Endometrium | Tumor | 3 | 1 | 3 |
| 1739 | UCEC | 55 | Female | Endometrium | Tumor | 1 | 1 | 1 |
| 2339 | UCEC | 79 | Female | Endometrium | Tumor | 2 | 1 | 2 |
| 2408 | UCEC | 57 | Female | Endometrium | Tumor | 2 | 1 | 2 |
| 2607 | UCEC | 81 | Female | Endometrium | Tumor | 2 | 1 | 2 |
| 2621 | UCEC | 58 | Female | Endometrium | Tumor | 2 | 2 | 4 |
| 3367 | UCEC | 70 | Female | Endometrium | Tumor | 1 | 1 | 1 |
| 3999 | UCEC | 70 | Female | Endometrium | Tumor | 2 | 1 | 2 |
| 4201 | UCEC | 62 | Female | Endometrium | Tumor | 3 | 1 | 3 |
| 4779 | UCEC | 87 | Female | Endometrium | Tumor | 0 | 0 | 0 |
| 4804 | UCEC | 82 | Female | Endometrium | Tumor | 2 | 2 | 4 |

Notes: ^a^: identity document. Clinical parameter data are available from v21.0.proteinatlas.org.
