# Supplementary material for: Pan-Cancer Analysis, Reveals COVID-19-Related BSG as a Novel Marker for Treatment and Identification of Multiple Human Cancers
Source: Front Cell Dev Biol. 2022 May 13;10:876180. doi: 10.3389/fcell.2022.876180 (PMC9136262; doi:10.3389/fcell.2022.876180)
Supplement: Supplementary file 14 [file Table5.docx]

**Supplementary Table S5.** Results of the covariates tests in comparing the difference in BSG protein levels.

| Cancer type | Clinical parameters | Sample number | | Fisher’s Exact test |
| --- | --- | --- | --- | --- |
|  |  | Normal | Tumor | *p-*value |
| Kidney | < 65 years old | 3 | 6 | 0.258 |
|  | ≥ 65 years old | 0 | 5 |  |
| LIHC | < 65 years old | 2 | 2 | 0.500 |
|  | ≥ 65 years old | 1 | 5 |  |
| THCA | < 65 years old | 3 | 0 | **0.029**^a^ |
|  | ≥ 65 years old | 0 | 4 |  |
| UCEC | < 65 years old | 3 | 5 | 0.209 |
|  | ≥ 65 years old | 0 | 6 |  |
| Kidney | Female | 1 | 5 | 1.000 |
|  | Male | 2 | 6 |  |
| LIHC | Female | 2 | 3 | 1.000 |
|  | Male | 1 | 4 |  |
| THCA | Female | 2 | 2 | 1.000 |
|  | Male | 1 | 2 |  |
| UCEC | Female | 3 | 11 | / |
|  | Male | 0 | 0 |  |

Notes: ^a^: The bold text indicates statistical significance.
