# Supplementary material for: Pan-Cancer Analysis, Reveals COVID-19-Related BSG as a Novel Marker for Treatment and Identification of Multiple Human Cancers
Source: Front Cell Dev Biol. 2022 May 13;10:876180. doi: 10.3389/fcell.2022.876180 (PMC9136262; doi:10.3389/fcell.2022.876180)
Supplement: Supplementary file 15 [file Table3.docx]

**Supplementary Table S3.** The criteria for staining intensity and the quantity scores.

| Scores | Immunohistochemical staining features for BSG protein | |
| --- | --- | --- |
|  | Staining intensity | Quantity |
| 0 | Negative | None |
| 1 | Weak | <25% |
| 2 | Moderate | 25%-75% |
| 3 | Strong | >75% |
