# Supplementary material for: Pan-Cancer Analysis, Reveals COVID-19-Related BSG as a Novel Marker for Treatment and Identification of Multiple Human Cancers
Source: Front Cell Dev Biol. 2022 May 13;10:876180. doi: 10.3389/fcell.2022.876180 (PMC9136262; doi:10.3389/fcell.2022.876180)
Supplement: Supplementary file 17 [file Table4.docx]

**Supplementary Table S4.** Results of the covariates tests in comparing the difference in BSG mRNA expression.

| Cancer type | Clinical parameters | Sample number | | | | Chi-square test^a^ | | | | Sample number^b^ | | | |  |
| --- | --- | --- | --- | --- | --- | --- | --- | --- | --- | --- | --- | --- | --- | --- |
|  |  | Normal | | Tumor | | Chi^2 | | *p-*value | | For comparing covariates | | For comparing BSG expression | |  |
| BLCA | < 65 years old | | 5 | | 150 | | 0.871 | | 0.351 | | 426 | | 426 | |
|  | ≥ 65 years old | | 14 | | 257 | |  | |  | |  | |  | |
| BRCA | < 65 years old | | 82 | | 748 | | 0.744 | | 0.388 | | 1203 | | 1205 | |
|  | ≥ 65 years old | | 31 | | 342 | |  | |  | |  | |  | |
| CHOL | < 65 years old | | 1 | | 17 | | 2.552 | | 0.110 | | 45 | | 45 | |
|  | ≥ 65 years old | | 8 | | 19 | |  | |  | |  | |  | |
| COAD | < 65 years old | | 11 | | 125 | | 4.205 | | **0.040**^c^ | | 327 | | 329 | |
|  | ≥ 65 years old | | 30 | | 161 | |  | |  | |  | |  | |
| ESCA | < 65 years old | | 5 | | 104 | | 1.090 | | 0.296 | | 194 | | 194 | |
|  | ≥ 65 years old | | 8 | | 77 | |  | |  | |  | |  | |
| GBM | < 65 years old | | 0 | | 96 | | /^d^ | | / | | 152 | | 158 | |
|  | ≥ 65 years old | | 0 | | 56 | |  | |  | |  | |  | |
| HNSCC | < 65 years old | | 24 | | 326 | | 1.252 | | 0.263 | | 561 | | 562 | |
|  | ≥ 65 years old | | 20 | | 191 | |  | |  | |  | |  | |
| KICH | < 65 years old | | 18 | | 51 | | 0.275 | | 0.600 | | 91 | | 91 | |
|  | ≥ 65 years old | | 7 | | 15 | |  | |  | |  | |  | |
| KIRC | < 65 years old | | 43 | | 330 | | 0.676 | | 0.411 | | 605 | | 605 | |
|  | ≥ 65 years old | | 32 | | 200 | |  | |  | |  | |  | |
| KIRP | < 65 years old | | 16 | | 172 | | 1.277 | | 0.258 | | 317 | | 320 | |
|  | ≥ 65 years old | | 16 | | 113 | |  | |  | |  | |  | |
| LIHC | < 65 years old | | 20 | | 220 | | 7.046 | | **0.008** | | 418 | | 419 | |
|  | ≥ 65 years old | | 30 | | 148 | |  | |  | |  | |  | |
| LUAD | < 65 years old | | 27 | | 220 | | 0.032 | | 0.858 | | 553 | | 572 | |
|  | ≥ 65 years old | | 32 | | 274 | |  | |  | |  | |  | |
| LUSC | < 65 years old | | 16 | | 169 | | 0.132 | | 0.716 | | 539 | | 548 | |
|  | ≥ 65 years old | | 34 | | 320 | |  | |  | |  | |  | |
| PAAD | < 65 years old | | 3 | | 82 | | 0.410 | | 0.522 | | 182 | | 182 | |
|  | ≥ 65 years old | | 1 | | 96 | |  | |  | |  | |  | |
| PCPG | < 65 years old | | 3 | | 153 | | 0.000 | | 1.000 | | 180 | | 180 | |
|  | ≥ 65 years old | | 0 | | 24 | |  | |  | |  | |  | |
| PRAD | < 65 years old | | 34 | | 329 | | 0.025 | | 0.875 | | 547 | | 547 | |
|  | ≥ 65 years old | | 18 | | 166 | |  | |  | |  | |  | |
| READ | < 65 years old | | 5 | | 47 | | 0.000 | | 1.000 | | 101 | | 102 | |
|  | ≥ 65 years old | | 5 | | 44 | |  | |  | |  | |  | |
| STAD | < 65 years old | | 13 | | 173 | | 0.521 | | 0.471 | | 445 | | 450 | |
|  | ≥ 65 years old | | 23 | | 236 | |  | |  | |  | |  | |
| THCA | < 65 years old | | 49 | | 428 | | 0.143 | | 0.706 | | 563 | | 563 | |
|  | ≥ 65 years old | | 10 | | 76 | |  | |  | |  | |  | |
| UCEC | < 65 years old | | 10 | | 84 | | 4.206 | | **0.040** | | 190 | | 203 | |
|  | ≥ 65 years old | | 3 | | 93 | |  | |  | |  | |  | |
| BLCA | Female | 9 | | 106 | | 4.188 | | **0.041** | | 426 | | 426 | |  |
|  | Male | 10 | | 301 | |  | |  | |  | |  | |  |
| BRCA | Female | 112 | | 1079 | | 0.000 | | 1.000 | | 1204 | | 1205 | |  |
|  | Male | 1 | | 12 | |  | |  | |  | |  | |  |
| CHOL | Female | 3 | | 20 | | 0.673 | | 0.412 | | 45 | | 45 | |  |
|  | Male | 6 | | 16 | |  | |  | |  | |  | |  |
| COAD | Female | 21 | | 130 | | 0.480 | | 0.489 | | 327 | | 329 | |  |
|  | Male | 20 | | 156 | |  | |  | |  | |  | |  |
| ESCA | Female | 5 | | 26 | | 3.604 | | 0.058 | | 194 | | 194 | |  |
|  | Male | 8 | | 155 | |  | |  | |  | |  | |  |
| GBM | Female | 0 | | 54 | | / | | / | | 152 | | 158 | |  |
|  | Male | 0 | | 98 | |  | |  | |  | |  | |  |
| HNSCC | Female | 14 | | 136 | | 0.642 | | 0.423 | | 562 | | 562 | |  |
|  | Male | 30 | | 382 | |  | |  | |  | |  | |  |
| KICH | Female | 12 | | 27 | | 0.372 | | 0.542 | | 91 | | 91 | |  |
|  | Male | 13 | | 39 | |  | |  | |  | |  | |  |
| KIRC | Female | 23 | | 186 | | 0.570 | | 0.450 | | 605 | | 605 | |  |
|  | Male | 52 | | 344 | |  | |  | |  | |  | |  |
| KIRP | Female | 10 | | 75 | | 0.401 | | 0.527 | | 320 | | 320 | |  |
|  | Male | 22 | | 213 | |  | |  | |  | |  | |  |
| LIHC | Female | 22 | | 120 | | 2.590 | | 0.108 | | 419 | | 419 | |  |
|  | Male | 28 | | 249 | |  | |  | |  | |  | |  |
| LUAD | Female | 34 | | 276 | | 0.312 | | 0.576 | | 572 | | 572 | |  |
|  | Male | 25 | | 237 | |  | |  | |  | |  | |  |
| LUSC | Female | 14 | | 129 | | 0.104 | | 0.748 | | 548 | | 548 | |  |
|  | Male | 36 | | 369 | |  | |  | |  | |  | |  |
| PAAD | Female | 2 | | 80 | | 0.000 | | 1.000 | | 182 | | 182 | |  |
|  | Male | 2 | | 98 | |  | |  | |  | |  | |  |
| PCPG | Female | 1 | | 100 | | 0.046 | | 0.830 | | 180 | | 180 | |  |
|  | Male | 2 | | 77 | |  | |  | |  | |  | |  |
| PRAD | Female | 0 | | 0 | | / | | / | | 547 | | 547 | |  |
|  | Male | 52 | | 495 | |  | |  | |  | |  | |  |
| READ | Female | 7 | | 42 | | 1.208 | | 0.272 | | 101 | | 102 | |  |
|  | Male | 3 | | 49 | |  | |  | |  | |  | |  |
| STAD | Female | 13 | | 146 | | 0.010 | | 0.919 | | 450 | | 450 | |  |
|  | Male | 23 | | 268 | |  | |  | |  | |  | |  |
| THCA | Female | 42 | | 368 | | 0.089 | | 0.765 | | 563 | | 563 | |  |
|  | Male | 17 | | 136 | |  | |  | |  | |  | |  |
| UCEC | Female | 13 | | 180 | | / | | / | | 193 | | 203 | |  |
|  | Male | 0 | | 0 | |  | |  | |  | |  | |  |

Notes: ^a^: One of the Chi-square test, Chi-squared test with Yates’ continuity correction or Fisher’s Exact test is utilized with the corresponding applicable conditions; ^b^: In some cancers, the sample number for comparing covariates is not equal to that for comparing BSG expression between cancers and controls, as no age or gender data of very few individuals had been recorded; ^c^: the bold text indicates statistical significance; ^d^: not applicable.
