# Supplementary material for: Pan-Cancer Analysis, Reveals COVID-19-Related BSG as a Novel Marker for Treatment and Identification of Multiple Human Cancers
Source: Front Cell Dev Biol. 2022 May 13;10:876180. doi: 10.3389/fcell.2022.876180 (PMC9136262; doi:10.3389/fcell.2022.876180)
Supplement: Supplementary file 2 [file DataSheet4.PDF]

Response to the Reviewer 2

March 20, 2022

Journal: Frontiers in Cell and Developmental Biology
Manuscript ID: 876180
Title: Pan-analysis Reveals COVID-19-related BSG as a Novel Marker for Treatment and Identification of Multiple Human Cancers

Dear Reviewer,

We thank you very much for the valuable suggestions for our manuscript. We have revised the manuscript according to your professional comments and suggestions.

The followings are responses to each point and properly addressed issues. A clean revised manuscript will be resubmitted simultaneously with the current response letter. Thanks a lot.

Best regard,

Independent Review Report, Reviewer 2

Summary of Review:

It is an interesting work to investigate the assocation of BSG with tumor outcome and drug response systematically by analyzing a large corhot of cancer patients. The analysis approaches are clear and appropriate, and the results are solid. Some comments are as follows.

Response: Thank you for your professional and kind comments.

1) The results about the association between BSG and different tumor types indicated the heterogeous both negative or positive associations. So the conclusion in this study should be revised to clearly introduce the difference and heterogeneity.

Response: We have revised the conclusion based on your comment, and an example can be seen below:

Abstract (Line 28–36 of page 1)

Results: Compared with normal tissues, *BSG* expression was highly expressed in 13 types of cancers (cholangiocarcinoma, etc.) and lowly expressed in colon adenocarcinoma and rectum adenocarcinoma. *BSG* expression was related to the prognosis of eight cancers (e.g., breast invasive carcinoma) ($p < .05$). The gene also demonstrated a pronounced effect in identifying 12 cancers (cholangiocarcinoma, etc.) from their control samples (AUC > 0.7). *BSG* expression was associated with DNA methyltransferases, mismatch repair genes, immune infiltration levels, tumor mutational burden, microsatellite instability, neoantigen, and immune checkpoints, suggesting the potential of *BSG* as an exciting target for cancer treatment. *BSG* may play its role in several cancers by affecting several signaling pathways such as drug cytochrome metabolism P450 and JAK-STAT.

2) There are NO figure legends to introduce/explain the figures.

Response: Sorry for such a mistake. We have uploaded the figure legends in the revised manuscript and a supplementary file called "Figure Legends.pdf" for review.

3) Figure 1 should be elaborated with text for each analysis step.

Response: In Figure 1, we desired to show an overview of our study. Indeed, in a single figure with limited space, we find it difficult to reveal all the analysis methods, as so many methods were used in our study. Instead, according to your professional suggestions, we have added an explanation to reveal each plot's details (at their bottom), which may help readers understand Figure 1 (please see below).

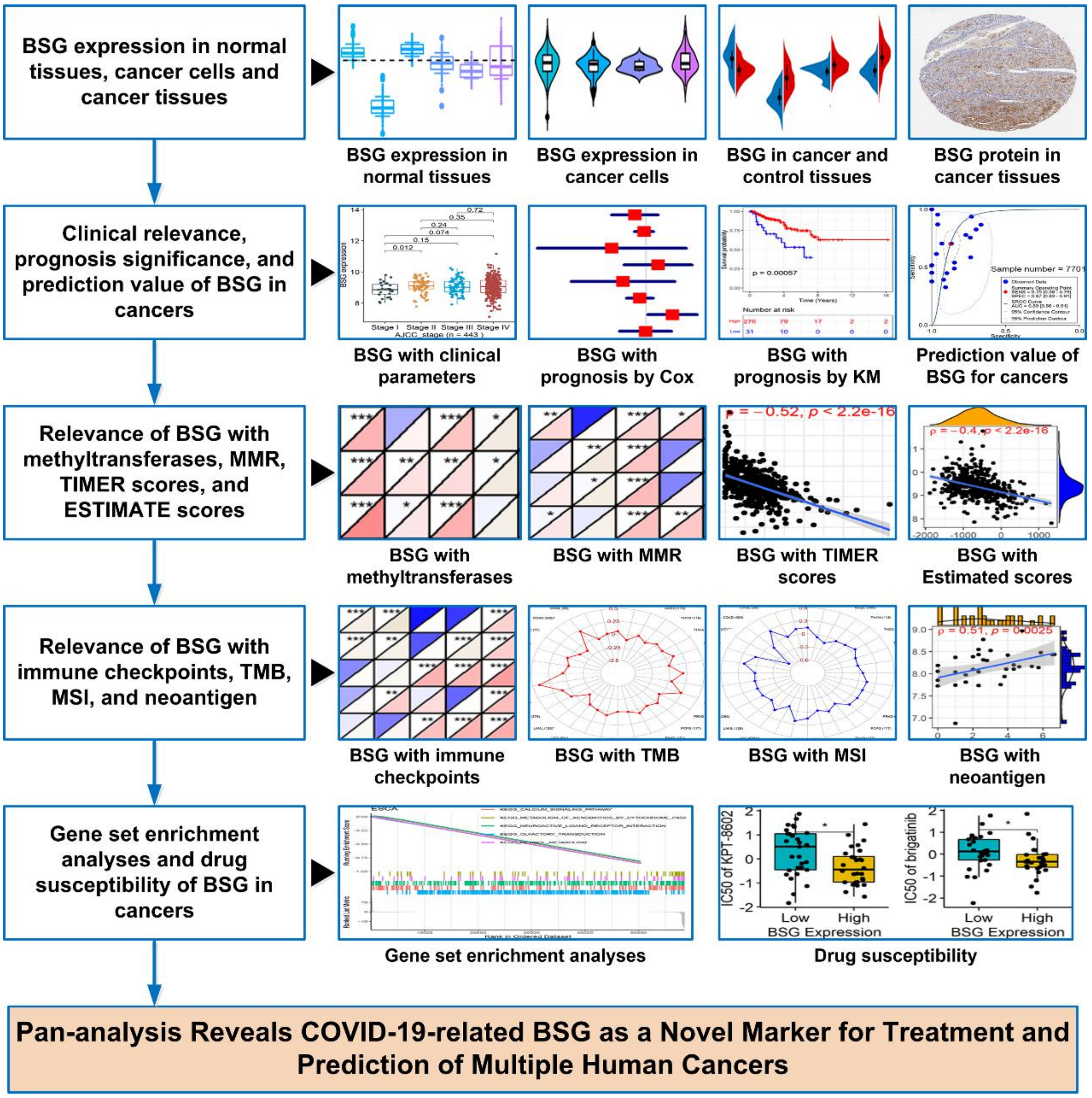

Figure 1. An overflow of this study. BSG, Basigin; "Cox" represents Cox regression analysis; "KM" represents Kaplan-Meier curves; MMR, mismatch repair genes; TMB, tumor mutational burden; MSI, microsatellite instability.

4) The conclusion section should be revised to introduce the discovery (rather than analysis methods)

Response: Thank you for your professional suggestions.

Abstract (Line 37 of page 2)

Conclusion:. *BSG* may be a novel biomarker for treating and identifying multiple human cancers.

5. Discussion (Line 421–423 of page 10)

This study revealed that COVID-19-related *BSG* was differently expressed in various human cancers. The gene is a prognostic marker for various cancers and has the potential to screen several cancers.

In a word, *BSG* may be a novel target for cancer treatment and identification.

5) Overall, it is a good study.
Response: Thank you for your professional suggestions, which greatly encouraged us.

Check List
a. Is the quality of the figures and tables satisfactory? -Yes
b. Does the reference list cover the relevant literature adequately and in an unbiased manner? -Yes
c. Are the statistical methods valid and correctly applied? (e.g. sample size, choice of test) -Yes
d. Is a statistician required to evaluate this study? -Yes
e. Are the methods sufficiently documented to allow replication studies? -Yes
Response: Thank you for your professional suggestions.

QUALITY ASSESSMENT:
Rigor: 3
Quality of the writing: 3
Overall quality of the content: 4
Interest to a general audience: 4
Response: Thanks for your comments. We have added more details about the statistical analysis in the revised manuscript. We have also carefully addressed language editing with the help of a native English speaker in our team.

Last, we have added one sentence in the Acknowledgements section for the valuable suggestions and careful work done by you, that is, "We are deeply grateful to anonymous reviewers for their detailed work and thoughtful suggestions, which have contributed greatly to the improvement of this article." (Line 547–548 of page 16).

We sincerely thank you for your comments and suggestions once again.
