# Supplementary material for: Pan-Cancer Analysis, Reveals COVID-19-Related BSG as a Novel Marker for Treatment and Identification of Multiple Human Cancers
Source: Front Cell Dev Biol. 2022 May 13;10:876180. doi: 10.3389/fcell.2022.876180 (PMC9136262; doi:10.3389/fcell.2022.876180)
Supplement: Supplementary file 9 [file DataSheet3.PDF]

Response to the Reviewer 1

March 20, 2022

Journal: Frontiers in Cell and Developmental Biology
Manuscript ID: 876180
Title: Pan-analysis Reveals COVID-19-related BSG as a Novel Marker for Treatment and Identification of Multiple Human Cancers

Dear Reviewer,

We thank you very much for the valuable suggestions for our manuscript. We have revised the manuscript according to your professional comments and suggestions.

The followings are responses to each point and properly addressed issues. A clean revised manuscript will be resubmitted simultaneously with the current response letter. Thanks a lot.

Best regard,

The Authors.

Independent Review Report, Reviewer 1

Summary of Review:

In this manuscript, Huang el.al report that Basigin (BSG), a factor essential for the infection and progression of COVID-19 as well as tumorigenesis of multiple tumors, could be a novel biomarker for the treatment against COVID-19 and multiple human cancers. Of note, previous reports, as the authors cite in their introduction, have described BSG as a therapeutic target for COVID-19 infection (Siska et al. 2021 PMID: 34779418, Ulrich et al. 2020 PMID: 32307653) and this molecule has been study for years as a target protein in different types of cancer (Hu et al. 2017 PMID: 28445958, Landras et al. PMID: 31744072). In this manuscript, the authors undertake a more detailed characterization of the BSG expression level and its potential clinical significance in pan-cancer and evaluated the BSG association with clinical relevance and immune filtration levels in cancers. The data presented here indicate that elevated BSG levels at the RNA and protein level, in certain types of cancer, is associated with tumor mutational burden, decreased immune cells infiltration into tumors, microsatellite instability, neoantigen, and increased immune checkpoints, suggesting the potential of BSG as an interesting target for cancer treatment. However, there are a substantial number of concerns that should be addressed before the manuscript is ready for publication; particularly regarding the methods, to confidently assess relevance of this study within current literature and to the clinic.

Response: We thank you very much for your professional suggestions and comments on our manuscript. The followings are responses to each point to issues. Thanks a lot.

Major points to be addressed:

1. Figure legends were not included in the latest manuscript uploaded for reviewers. The main purpose of a figure legend is to enable the reader to understand a figure without having to refer to the main text of the manuscript. The authors need to include this in the manuscript for a better interpretation of the data. In the figure legends, the authors need to include a brief title that applies to the entire figure, including all panels; a description of the techniques used; a statement of the results that can be gleaned from the particular figure; an explanation of features in the figure, a brief explanation of the statistical analysis performed and include number of samples use in each panel.

Response: Thank you for your professional suggestions. We have included figure legends for each Figure in our manuscript. We have also provided information in the figures or figure legends according to your advice, including but not limited to a brief title, a description of the techniques, statistical analysis, and the number of samples. An example can be seen below:

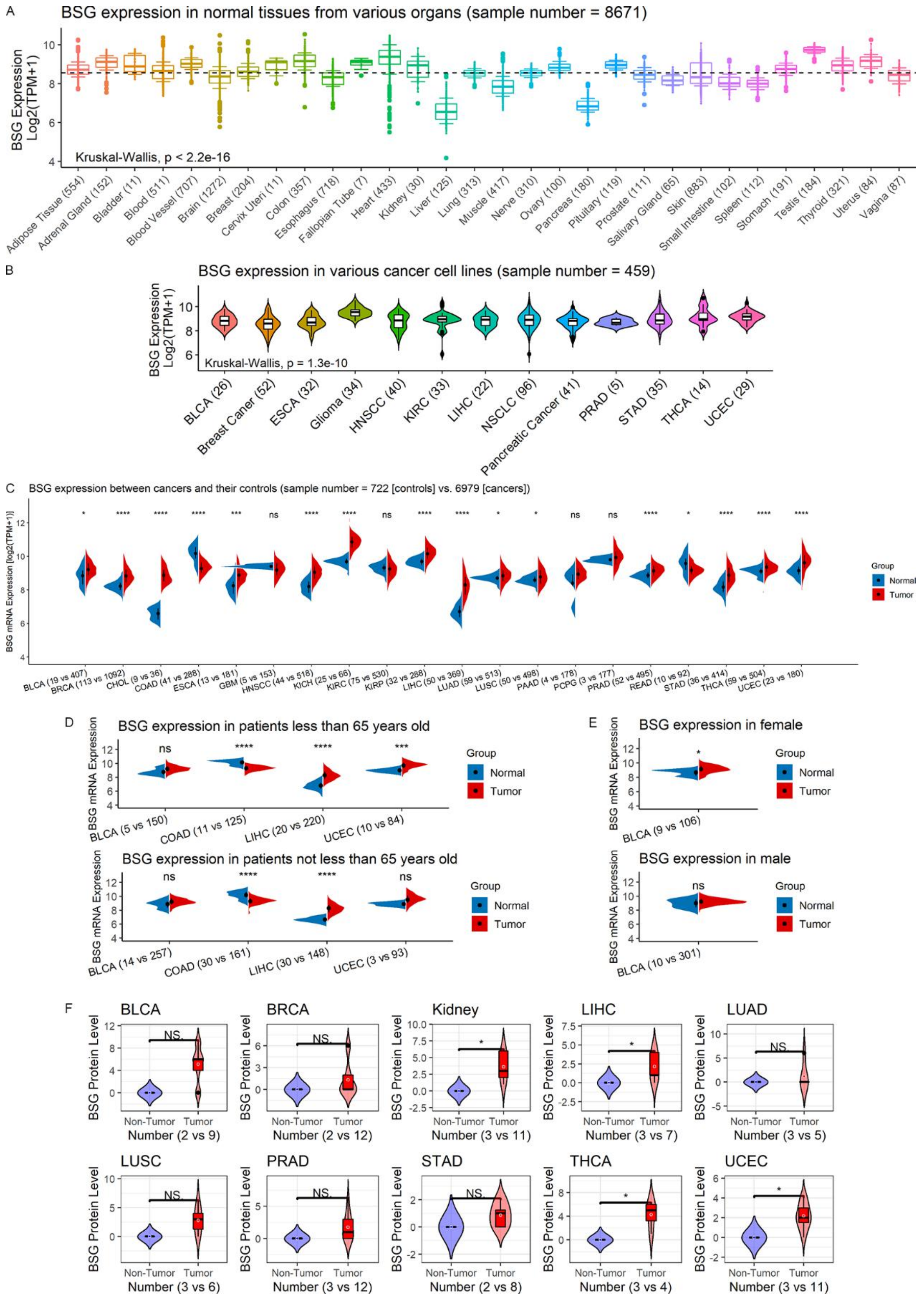

Figure 2. BSG expression in normal tissues and pan-cancer at mRNA and protein levels. Panel A: Distinct BSG expression in various normal tissues of humans; the dashed line represents the average value (equaling to 8.549) of BSG expression in all normal tissues shown in this panel. Panel B: Different BSG expressions in various cancer cell lines of humans. Panel C: Different BSG expressions between cancer tissues with their normal tissues. Panels D–E: The hierarchical analysis of BSG expression differences in some cancers and their control tissues. Panel F: Elevated BSG

levels in several cancers were validated at the protein level. For panels A and B: the value in parentheses represents the number of samples. For panels C–E: the two values (e.g., "n = 19 vs 407" for BLCA) in parentheses represent the numbers of controls and cancers, respectively. For panels C–F: each *p*-value in this figure is based on the Wilcoxon rank-sum test, and then *p*-values in panels C–E are adjusted by multiple comparations with the false discovery rate method; $^{*}p < 0.05$, $^{**}p < 0.01$, $^{***}p < 0.001$. BLCA, bladder cancer; ESCA, esophageal carcinoma; HNSCC, head and neck squamous cell carcinoma; KIRC, kidney renal clear cell carcinoma; LIHC, liver hepatocellular carcinoma; NSCLC, non-small-cell lung carcinoma; PRAD, prostate adenocarcinoma; STAD, stomach adenocarcinoma; THCA, thyroid carcinoma; UCEC, uterine corpus endometrial carcinoma; BRCA, breast invasive carcinoma; CHOL, cholangiocarcinoma; COAD, colon adenocarcinoma; KICH, kidney chromophobe; KIRP, kidney renal papillary cell carcinoma; LUAD, lung adenocarcinoma; LUSC, lung squamous cell carcinoma; READ, rectum adenocarcinoma.

2. The methods do not contain enough detail for readers to think critically on reported data and the authors conclusions. At the very least, the following subpoints need to be clarified regarding the methods.

2.1 Supplemental Table 1 raises a major potential flaw in the methods. first of all, this supplementary table has not been added in the main text to understand where all this data is used. Second, in the methods, line 97-106 it is not clear whether the normal tissues (non-Tumor) come from the same patients as the tumor tissues. Clarification is needed to understand how the comparative analysis should be performed, for example if this could be a pairwise.

Response: Thanks for your comments. Above all, in this revision, Supplementary Table 1 has been changed to Supplementary Table 2, and we have cited it in the main text. The normal tissues do not come from the same patients as the tumor samples. Thus it is not feasible to perform a pairwise (please see below).

3. Materials and Methods (Line 100–109 of page 3)

3.2 Collection of BSG protein level data

Immunohistochemical staining data for validating the BSG protein levels in cancer and normal tissues were obtained from The Human Protein Atlas (proteinatlas.org) (Uhlen et al. 2015) by using the "HPAanalyze" package (Tran et al. 2019) in R (v4.1.0). BSG protein levels of cancers with no fewer than two samples in both normal tissues and cancer tissues were chosen in this study. For one cancer with more than one kind of antibody, data on antibodies used to detect more samples of this cancer were included. Eventually, 109 samples were selected for further analysis, and these samples' identity documents, groups, and tissue types were stored in **Supplementary Table 2**. Normal tissues and tumor tissues were obtained from various individuals, and no person provided both normal and tumor samples.

2.2 In the statistical analysis section, since in this study they have a large sample size, with a big age

range; the authors need to clarify:
2.2.1 The statistical test(s) used AND whether they are one- or two-sided

Response: We have provided all details about the statistical tests and the condition on one- or two-sides:

4. Statistical analysis (Line 155–180 of pages 4–5)

The Kruskal–Wallis test was selected to assess the difference in *BSG* expression in various normal tissues and cancer cell lines. Wilcoxon rank-sum test was used to assess the differential expression of BSG (at both mRNA and protein levels) between cancer tissues and their controls. Multiple comparations by the false discovery rate method were applied to verify the distinct expression levels of *BSG* mRNA between cancers and controls. The homogeneity between cancer and control groups was evaluated using the chi-square test or Fisher's exact test.

Wilcoxon rank-sum test was utilized to explore the relationship between *BSG* expression and the three clinical features (AJCC stage, age, and gender). Additionally, if *BSG* expression were detected associated with not less than two clinical parameters (e.g., age and gender), the adjustment test would be performed: the correlation between *BSG* expression and age would be analyzed hierarchically based on various genders.

Using "survival" and "forestplot" packages in R (v4.1.0), univariate Cox regression analysis and Kaplan-Meier plots were applied to measure the relevance between *BSG* expression and OS, DSS, DFI, and PFI. The optimal cutpoint for high- and low- *BSG* expression levels in each Kaplan-Meier curve was determined by utilizing the maximally selected rank statistics from the "maxstat" and "survminer" R packages. For detecting the accuracy of *BSG* expression in identifying cancers from controls, the specificity, sensitivity, and the area under the curve (AUC) of the receiver operating characteristics (ROC) were calculated using the "pROC" package (Robin et al. 2011) for R (v4.1.0), and the specificity, sensitivity, and the AUC of a summary ROC was determined in Stata (v15.0).

Spearman's rank correlation analyses were applied to evaluate the correlations of *BSG* expression with DNMT, immune environment, TMB, MSI, neoantigen count, immune checkpoints, and leading-edge genes. Wilcoxon rank-sum test was utilized to determine the difference in abundance of six kinds of immune cells and the difference in IC50 of certain drugs between high- and low- *BSG* expression groups.

In this study, p-value < 0.05 demonstrated statistical significance, and except for drug-sensitive analysis, all p-values were two-sided. An overflow of the study can be viewed in **Figure 1**.

2.2.2 A description of all covariates tested

Response: To be honest, we previously did not perform covariates tests in our study. According to your advice, we realized that it is necessary to consider covariates tests to compare the difference in BSG expression between cancer and normal tissues and explore the association of BSG expression

with clinical parameters. All the changes can be seen below.

4. Statistical analysis (Line 155–165 of page 4)

The Kruskal–Wallis test was selected to assess the difference in *BSG* expression in various normal tissues and cancer cell lines. Wilcoxon rank-sum test was used to assess the differential expression of BSG (at both mRNA and protein levels) between cancer tissues and their controls. Multiple comparations by the false discovery rate method were applied to verify the distinct expression levels of *BSG* mRNA between cancers and controls. The homogeneity between cancer and control groups was evaluated using the chi-square test or Fisher's exact test.

Wilcoxon rank-sum test was utilized to explore the relationship between *BSG* expression and the three clinical features (AJCC stage, age, and gender). Additionally, if *BSG* expression were detected associated with not less than two clinical parameters (e.g., age and gender), the adjustment test would be performed: the correlation between *BSG* expression and age would be analyzed hierarchically based on various genders.

2.2.3 A description of any assumptions or corrections, such as tests of normality and adjustment for multiple comparisons, for example is the data adjusted for age and sex to look at the differences in BSG expression by AJCC stages in figure 2D? or to compare the expression of BSG in the tumor versus non-tumor tissue in the different types of cancer in Figure 3A?

Response: Indeed, no tests of normality of our study have been used with the fact that: (1) the statistical methods we use are suitable for both normal and non-normal distribution data, such as Wilcoxon rank-sum test for Figures 2–3 and Spearman's rank correlation analyses for Figures 7–10; and (2) logarithmic transformation can transform original data into new data with approximate normal distribution, and the expression data used for calculating in our study has been processed using $\log_2 (x + 1)$ (please view below).

3. Materials and Methods (Line 90–91 of page 3)

3.1 Collection of BSG mRNA expression, clinical characteristics, and prognosis data

All gene expression levels in data obtained from GTEx, CCLE, and TCGA were processed in R (v4.1.0) using $\log_2 (x + 1)$.

According to your professional suggestions, we think it necessary to perform adjustments for multiple comparisons in comparing the BSG expression in various clinical parameters in the previous Figure 2D (it has been changed to Figures 3B–D), and the changes are as follows:

3.6 Statistical analysis (Line 161–165 of page 4)

Wilcoxon rank-sum test was utilized to explore the relationship between *BSG* expression and the three clinical features (AJCC stage, age, and gender). Additionally, if *BSG* expression were detected associated with not less than two clinical parameters (e.g., age and gender), the adjustment test would be performed: the correlation between *BSG* expression and age would be analyzed hierarchically based on various genders.

4. Results (Line 218–235 of page 6)

4.2 Relevance between BSG expression and clinical parameters

Different clinical parameters may result in the different prognoses of cancer patients. For example, patients with cervical cancer and with advanced AJCC stages tended to have poor prognoses (Olawaiye et al. 2021). The other example was that elder age, and male gender were risk factors for patients with oral squamous cancers (Oh et al. 2021). Thus, the relationship between *BSG* expression and the three clinical parameters—AJCC stages, age, and gender—of patients was explored in neoplasms with the clinical parameters data. The immediate relevance of *BSG* expression with AJCC stages was found in HNSCC, KICH, LIHC, PAAD, SKCM, and THCA ($p < .05$; **Figure 3A**), while such a phenomenon was not in the other cancers (ACC, etc.) (**Supplementary Figure 1**). Higher *BSG* expression was detected in elderly (65 years old or older) BRCA patients ($p < .05$), and based on the LOESS (locally weighted regression) age trajectory, older patients tend to be found increasing *BSG* expression levels (**Figure 3B**). Elevated *BSG* expression was generally be observed in young (< 65 years old) PRAD patients ($p < .05$); however, the LOESS age trajectory suggested some fluctuations in the correlation between age and *BSG* expression (**Figure 3C**). There was no correlation between *BSG* expression and ages in other 30 cancers (e.g., ACC; **Supplementary Figure 2**). In terms of gender, males with BRCA or KIRP had overexpression of *BSG*, while the contrast phenomenon was found in patients with KICH or LIHA ($p < .05$; **Figure 3D**); and for other cancers (ACC, etc.), there was no statistical difference in *BSG* expression between patients with different genders (**Supplementary Figure 3**).

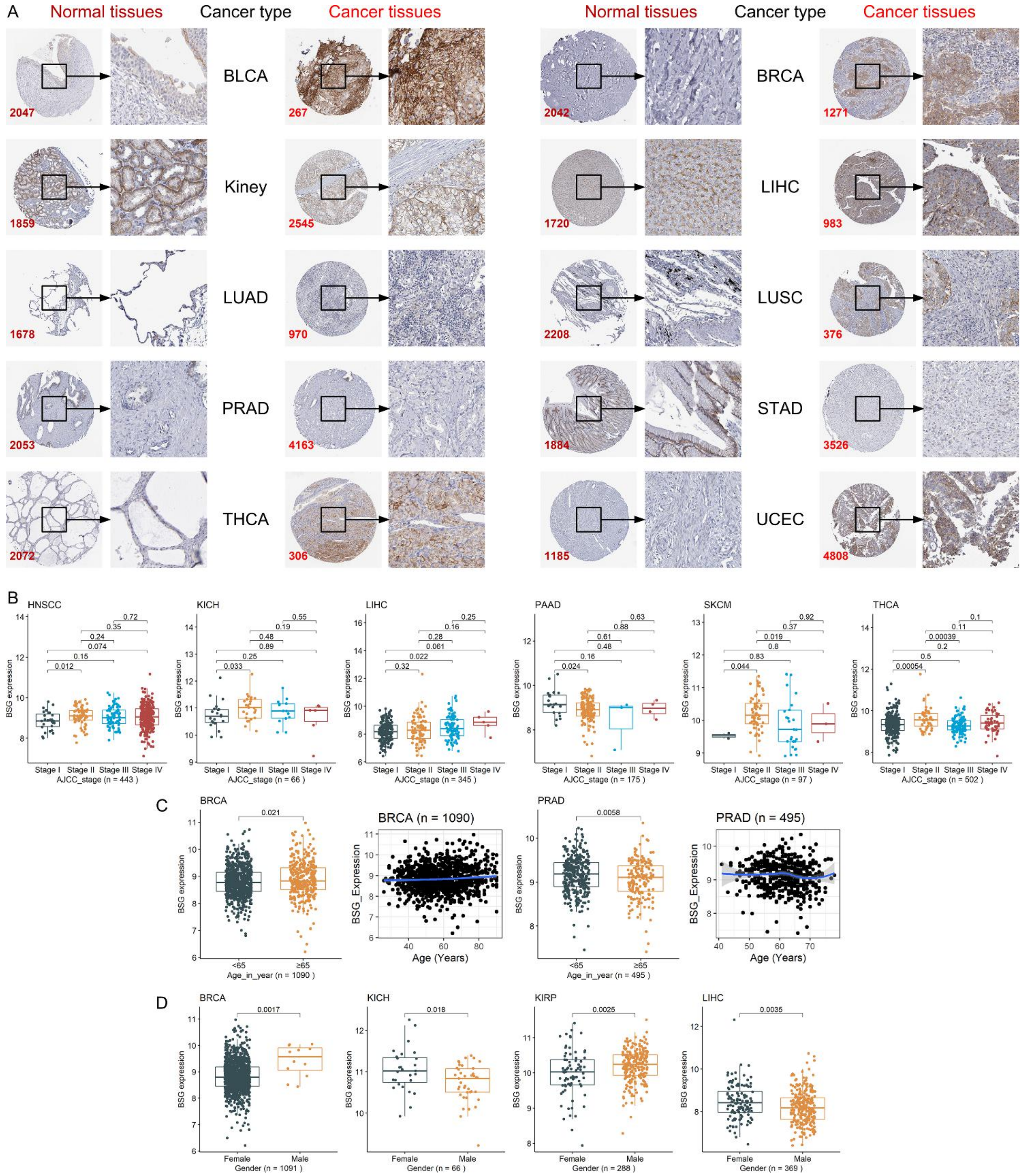

Figure 3. The staining intensity of the anti-BSG antibodies in the ten cancers and the correlations between BSG expression and clinical parameters. Panel A: Under the microscope, the staining intensity of the anti-BSG antibodies in the ten cancer tissues is stronger than that in normal tissues; images are available from v21.0.proteinatlas.org. In panel A, the value of the lower-left corner of the images (e.g., 2047 for BLCA) is the identity document of patients or normal individuals. Panels B–D: The close correlations between BSG expression with clinical parameters; all *p*-values in the three panels were based on the Wilcoxon rank-sum test. The two plots with a blue curve of panel C are drawn based on the locally weighted regression. The value in parentheses (e.g., "n = 443" for

HNSCC) represents the number of samples. BLCA, bladder cancer; BRCA, breast invasive carcinoma; LIHC, liver hepatocellular carcinoma; LUAD, lung adenocarcinoma; LUSC, lung squamous cell carcinoma; PRAD, prostate adenocarcinoma; STAD, stomach adenocarcinoma; THCA, thyroid carcinoma; UCEC, uterine corpus endometrial carcinoma; HNSCC, head and neck squamous cell carcinoma; KICH, kidney chromophobe; PAAD, pancreatic adenocarcinoma; SKCM, skin cutaneous melanoma.

For the previous Figure 3A (it has been changed to Figure 2F): (1) In Figure 2F, each cancer data comes from a separate dataset; (2) Each *p*-value corresponds to a unique conclusion (for example, the difference in BSG protein levels between BLCA and normal tissues does not affect the presence of BSG overexpression in LIHC tissues); and (3) In Figure 2F, the current positive results ($p < 0.05$) are relatively few (4/10, while that in Figure 2C is up to 16/20), and multiple comparisons may mask the positive results. Thus, may we do not perform multiple comparisons for Figure 2F?
Instead, based on your professional comments, we realize that the adjustments for multiple comparisons should be carried out to compare BSG expression in the tumor versus non-tumor tissue in the current Figure 2C, which can be seen in Figures 2D and E. Details about the revision can be seen below:

4. Results (Line 188–205 of page 5)

4.1 Differently expressed BSG in human cancers

In detail, upregulated *BSG* expression was observed in 14 cancers—bladder cancer (BLCA), breast invasive carcinoma (BRCA), cholangiocarcinoma (CHOL), esophageal carcinoma (ESCA), head and neck squamous cell carcinoma (HNSCC), kidney chromophobe (KICH), kidney renal papillary cell carcinoma (KIRP), LIHC, LUAD, LUSC, prostate adenocarcinoma (PRAD), stomach adenocarcinoma (STAD), thyroid carcinoma (THCA), and uterine corpus endometrial carcinoma (UCEC); downregulated *BSG* expression was detected in two cancers—colon adenocarcinoma (COAD) and rectum adenocarcinoma (READ) ($p < .05$; **Figure 2C**). Notably, In COAD, LIHC, and UCEC, the age distribution between cancer and control groups was statistically different. Similarly, in BLCA, there was a difference in gender distribution between the cancer group and the control group (**Supplementary Table 4)**. In order to exclude the potential effects of gender and age covariates in the difference of *BSG* expression between cancer and control groups, a hierarchical evaluation was performed for these four cancers. As a result, no statistical difference in *BSG* expression between BLCA tissue and its control tissue for both the old and the young (**Figure 2D**). Increasing levels of *BSG* were observed in female BLCA patients instead of male BLCA patients (**Figure 2E**). Thus, age distribution rather than gender distribution may contribute to the difference in BSG expression in BLCA. For UCEC, upregulated *BSG* expression was found in cancer tissues for the young, while this cannot be concluded for the old (**Figure 2D**). Therefore, the distinct expression of *BSG* was observed in 15 instead of 16 cancers (not for BLCA).

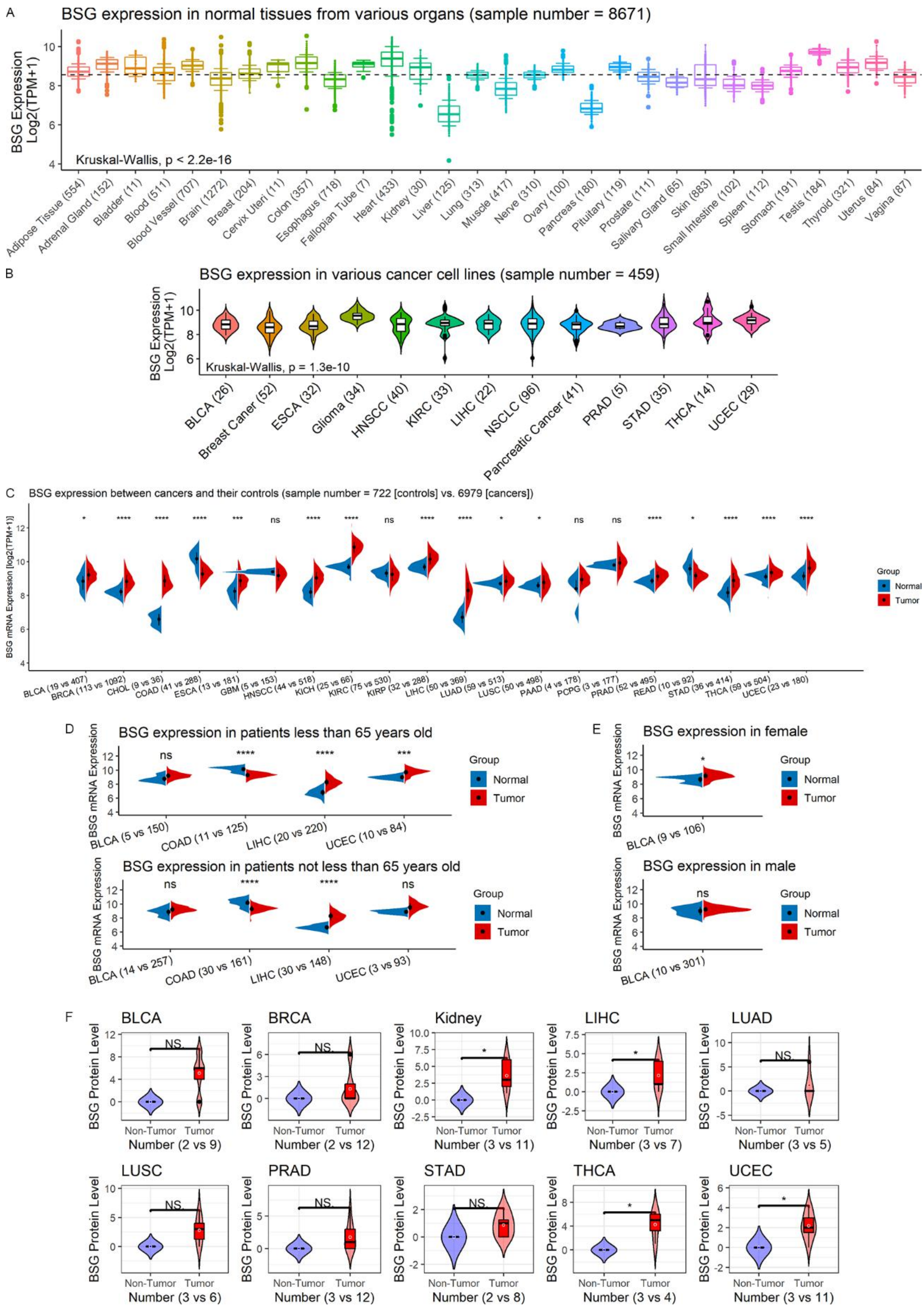

Figure 2. BSG expression in normal tissues and pan-cancer at mRNA and protein levels. Panel A: Distinct BSG expression in various normal tissues of humans; the dashed line represents the average value (equaling to 8.549) of BSG expression in all normal tissues shown in this panel. Panel B: Different BSG expressions in various cancer cell lines of humans. Panel C: Different BSG expressions between cancer tissues with their normal tissues. Panels D–E: The hierarchical analysis

of BSG expression differences in some cancers and their control tissues. Panel F: Elevated BSG levels in several cancers were validated at the protein level. For panels A and B: the value in parentheses represents the number of samples. For panels C–E: the two values (e.g., "n = 19 vs 407" for BLCA) in parentheses represent the numbers of controls and cancers, respectively. For panels C–F: each *p*-value in this figure is based on the Wilcoxon rank-sum test, and then *p*-values in panels C–E are adjusted by multiple comparations with the false discovery rate method; $^{*}p < 0.05$, $^{**}p < 0.01$, $^{***}p < 0.001$. BLCA, bladder cancer; ESCA, esophageal carcinoma; HNSCC, head and neck squamous cell carcinoma; KIRC, kidney renal clear cell carcinoma; LIHC, liver hepatocellular carcinoma; NSCLC, non-small-cell lung carcinoma; PRAD, prostate adenocarcinoma; STAD, stomach adenocarcinoma; THCA, thyroid carcinoma; UCEC, uterine corpus endometrial carcinoma; BRCA, breast invasive carcinoma; CHOL, cholangiocarcinoma; COAD, colon adenocarcinoma; KICH, kidney chromophobe; KIRP, kidney renal papillary cell carcinoma; LUAD, lung adenocarcinoma; LUSC, lung squamous cell carcinoma; READ, rectum adenocarcinoma.

2.2.4 The authors do not provide enough detail for readers to clearly interpret the statistical analysis used in each figure, all this information should be included in this methods section and in the figure legends.

Response: Thanks for your advice. In this revision, we have provided as many details as possible in the statistical analysis section and figure legends regarding the statistical methods used in this study. The statistical analysis section and an example of figure legends can be seen below:

3.6 Statistical analysis (Line 155–180 of pages 4–5)

The Kruskal–Wallis test was selected to assess the difference in *BSG* expression in various normal tissues and cancer cell lines. Wilcoxon rank-sum test was used to assess the differential expression of BSG (at both mRNA and protein levels) between cancer tissues and their controls. Multiple comparations by the false discovery rate method were applied to verify the distinct expression levels of *BSG* mRNA between cancers and controls. The homogeneity between cancer and control groups was evaluated using the chi-square test or Fisher's exact test.

Wilcoxon rank-sum test was utilized to explore the relationship between *BSG* expression and the three clinical features (AJCC stage, age, and gender). Additionally, if *BSG* expression were detected associated with not less than two clinical parameters (e.g., age and gender), the adjustment test would be performed: the correlation between *BSG* expression and age would be analyzed hierarchically based on various genders.

Using "survival" and "forestplot" packages in R (v4.1.0), univariate Cox regression analysis and Kaplan-Meier plots were applied to measure the relevance between *BSG* expression and OS, DSS, DFI, and PFI. The optimal cutpoint for high- and low- *BSG* expression levels in each Kaplan-Meier curve was determined by utilizing the maximally selected rank statistics from the "maxstat" and "survminer" R packages. For detecting the accuracy of *BSG* expression in identifying cancers from controls, the specificity, sensitivity, and the area under the curve (AUC) of the receiver operating characteristics (ROC) were calculated using the "pROC" package (Robin et al. 2011) for R (v4.1.0),

and the specificity, sensitivity, and the AUC of a summary ROC was determined in Stata (v15.0).

Spearman's rank correlation analyses were applied to evaluate the correlations of *BSG* expression with DNMT, immune environment, TMB, MSI, neoantigen count, immune checkpoints, and leading-edge genes. Wilcoxon rank-sum test was utilized to determine the difference in abundance of six kinds of immune cells and the difference in IC50 of certain drugs between high- and low- *BSG* expression groups.

In this study, p-value < 0.05 demonstrated statistical significance, and except for drug-sensitive analysis, all p-values were two-sided. An overflow of the study can be viewed in **Figure 1**.

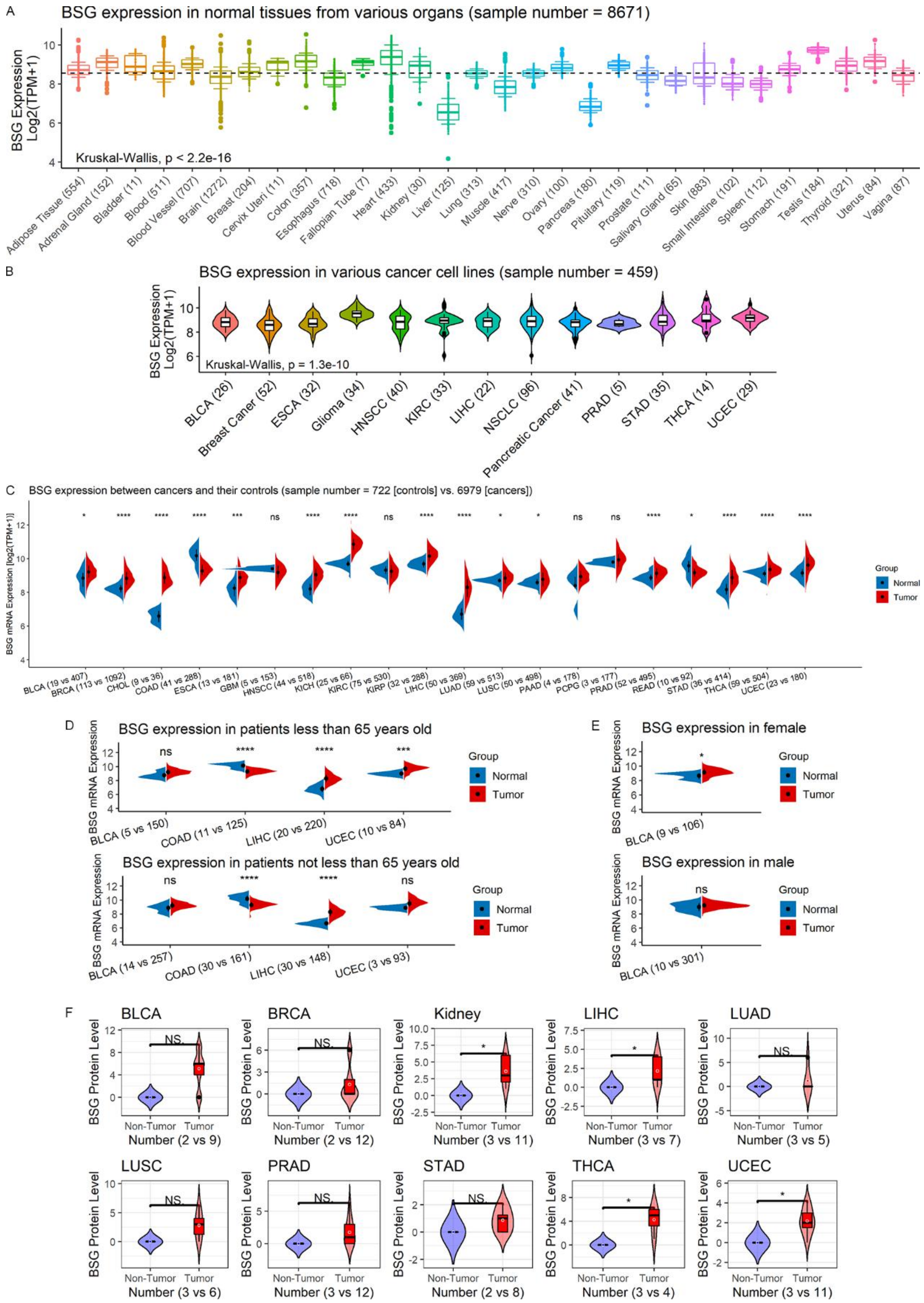

Figure 2. BSG expression in normal tissues and pan-cancer at mRNA and protein levels. Panel A: Distinct BSG expression in various normal tissues of humans; the dashed line represents the average value (equaling to 8.549) of BSG expression in all normal tissues shown in this panel. Panel B: Different BSG expressions in various cancer cell lines of humans. Panel C: Different BSG expressions between cancer tissues with their normal tissues. Panels D–E: The hierarchical analysis of BSG expression differences in some cancers and their control tissues. Panel F: Elevated BSG

levels in several cancers were validated at the protein level. For panels A and B: the value in parentheses represents the number of samples. For panels C–E: the two values (e.g., "n = 19 vs 407" for BLCA) in parentheses represent the numbers of controls and cancers, respectively. For panels C–F: each p-value in this figure is based on the Wilcoxon rank-sum test, and then p-values in panels C–E are adjusted by multiple comparations with the false discovery rate method; $^{*}p < 0.05$, $^{**}p < 0.01$, $^{***}p < 0.001$. BLCA, bladder cancer; ESCA, esophageal carcinoma; HNSCC, head and neck squamous cell carcinoma; KIRC, kidney renal clear cell carcinoma; LIHC, liver hepatocellular carcinoma; NSCLC, non-small-cell lung carcinoma; PRAD, prostate adenocarcinoma; STAD, stomach adenocarcinoma; THCA, thyroid carcinoma; UCEC, uterine corpus endometrial carcinoma; BRCA, breast invasive carcinoma; CHOL, cholangiocarcinoma; COAD, colon adenocarcinoma; KICH, kidney chromophobe; KIRP, kidney renal papillary cell carcinoma; LUAD, lung adenocarcinoma; LUSC, lung squamous cell carcinoma; READ, rectum adenocarcinoma.

2.3 For figure 7A, in the statistical analysis section, more details should be provided about what statistical modeling was used. For example, the authors should explain: "we measure the specificity and sensitivity of BSG expression, calculating the area under the curve (AUC) of the receiver operating characteristics (ROC) using the "AUC" and "pROC" packages for R."

Response: Thanks for your comments. We have provided details about the statistical methods used in our study (please see the response to comment 2.2.4).

3. Throughout the manuscript, the description of sample and replicate numbers is unclear, the authors are not mentioning this in the methods or the main text. The authors need to provide the exact sample size (n) for each experimental group/condition, given as a discrete number and unit of measurement. This is especially important in establishing confidence in the results as several experiments appear to have low Ns, for example in figure 3A. Please add this information for figure 2, 5, 6, 7 and 8.

Response: According to your suggestions, we have added the sample size for Figures 2, 3, 4, 5, 6, 7, and 8. An example can be seen above (Figure 2 shown in response to comment 2.2.4):

4. In figure 2D line 156-163, the authors show how the expression of BSG is highly variable in different types of cancer and each AJCC stages. They also show how the expression of BSG is affected by age in some types of cancer. For example, being elevated in elderly in BRCA patients but decreased in PRAD patients compared with the young group, as described in Figure 2D. As presented here, these results are showing that not all the types of cancer will cause a high risk of COVID-19 based on BSG levels. To test this hypothesis, ideally it would be great to have the comparison of the COVID-19 pathology severity in patients that were diagnosed of COVID-19 that also have type of cancers with high and low BSG expression and identify if those patients with higher expression of BSG will have a worse prognosis of COVID-19.

Response: Thanks for your so professional suggestions. We realize that it will undoubtedly be better

to perform the validation work suggested by you. Unfortunately, to be honest, we find it difficult to reach the task with the fact that:

(1) It is hard to collect insufficient in-house samples from coronavirus disease 2019 (COVID-19) patients in our institutions (hospitals). Worse still, due to the management and control of the in our cities, it is almost impossible to collect enough clinical COVID-19 samples now and for a long time.

(2) To our best knowledge, no data of patients with both COVID-19 and cancers can be found in public databases (e.g., Gene Expression Omnibus) can be utilized up to date. Even though a dataset about hematologic cancer with COVID-19 can be found (please click: https://www.ncbi.nlm.nih.gov/geo/query/acc.cgi?acc=GSE153610), it is not suitable for our research, as no hematologic cancer was investigated in our study).

(3) Thanks to your comment, we find it a good direction to be considered in the future. We realize that the lack of such validation is a limitation in our study, which we have identified in the discussion section (please see below).

5. Discussion (Line 416–420 of page 10)

Some limitations to this study should be concerned. Above all, we failed to collect samples from patients diagnosed with both COVID-19 and cancers, and thus we can not directly identify whether these patients had poor prognoses in our study. Specimens utilized for exploring BSG protein levels are relatively limited. The potential mechanisms of *BSG* in multiple cancers requires experimental validation.

5. For Figure 3A, line 150-152, the authors are comparing the tumor tissue with the corresponding normal tissues (non-Tumor).

5.1 The analysis of the comparison of the BSG protein expression should be performed using a paired test, this is not what the authors are showing if figure 3A.

Response: For Figure 2F (the previous Figure 3A), the normal tissues do not come from the same patients as the tumor samples, and thus it is not feasible to perform a paired. We are sorry that we did not state it before, and we have revised the content (please see below)

3. Materials and Methods (Line 100–109 of page 3)

3.2 Collection of BSG protein level data

Immunohistochemical staining data for validating the BSG protein levels in cancer and normal tissues were obtained from The Human Protein Atlas (proteinatlas.org) (Uhlen et al. 2015) by using the "HPAanalyze" package (Tran et al. 2019) in R (v4.1.0). BSG protein levels of cancers with no fewer than two samples in both normal tissues and cancer tissues were chosen in this study. For one cancer with more than one kind of antibody, data on antibodies used to detect more samples of this cancer were included. Eventually, 109 samples were selected for further analysis, and these samples'

identity documents, groups, and tissue types were stored in **Supplementary Table 2**. Normal tissues and tumor tissues were obtained from various individuals, and no person provided both normal and tumor samples.

5.2 Also, clarification is needed about the group number showed in the figure; is this the number of ROI from one tissue or this is the number of participants used in this analysis? Furthermore, authors need to show the individuals values of the BSG expression in the violin plot with the connecting line for the values of the same participant in the tumor and non-tumor category to make this easier to interpret by the readers. In addition, for figure 3A, line 152-155, the authors highlighted that there is no statistical significance, in the BSG protein levels in in BLCA, BRCA, LUAD, LUSC, PRAD, and STAD, but in Figure 3B and Figure 4 they show examples with stronger levels of BSG in those cancer types. As I suggested before, using a paired test will help to visualize these differences.

Response: For Figure 2F, the value in brackets represents the number of samples utilized in this analysis, and we have stated it in the figure legend in this revision.

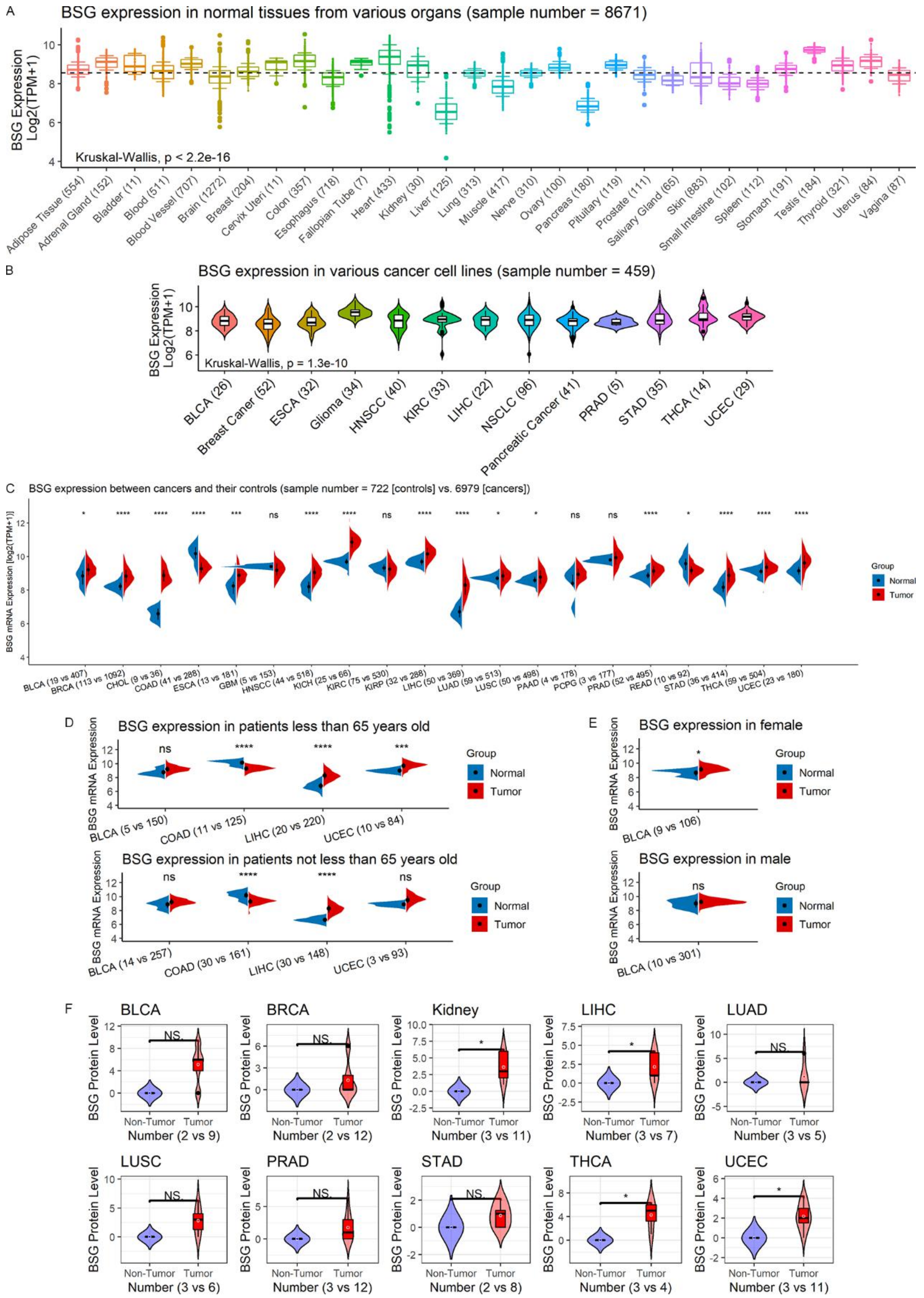

Figure 2. BSG expression in normal tissues and pan-cancer at mRNA and protein levels. Panel A: Distinct BSG expression in various normal tissues of humans; the dashed line represents the average value (equaling to 8.549) of BSG expression in all normal tissues shown in this panel. Panel B: Different BSG expressions in various cancer cell lines of humans. Panel C: Different BSG expressions between cancer tissues with their normal tissues. Panels D–E: The hierarchical analysis of BSG expression differences in some cancers and their control tissues. Panel F: Elevated BSG

levels in several cancers were validated at the protein level. For panels A and B: the value in parentheses represents the number of samples. For panels C–E: the two values (e.g., "n = 19 vs 407" for BLCA) in parentheses represent the numbers of controls and cancers, respectively. For panels C–F: each *p*-value in this figure is based on the Wilcoxon rank-sum test, and then *p*-values in panels C–E are adjusted by multiple comparations with the false discovery rate method; $^{*}p < 0.05$, $^{**}p < 0.01$, $^{***}p < 0.001$. BLCA, bladder cancer; ESCA, esophageal carcinoma; HNSCC, head and neck squamous cell carcinoma; KIRC, kidney renal clear cell carcinoma; LIHC, liver hepatocellular carcinoma; NSCLC, non-small-cell lung carcinoma; PRAD, prostate adenocarcinoma; STAD, stomach adenocarcinoma; THCA, thyroid carcinoma; UCEC, uterine corpus endometrial carcinoma; BRCA, breast invasive carcinoma; CHOL, cholangiocarcinoma; COAD, colon adenocarcinoma; KICH, kidney chromophobe; KIRP, kidney renal papillary cell carcinoma; LUAD, lung adenocarcinoma; LUSC, lung squamous cell carcinoma; READ, rectum adenocarcinoma.

The protein levels of BSG were evaluated by staining scores of the same antibody. The criteria for these scores have been revealed in the text and Supplementary Table 3.

3. Materials and Methods (Line 113–117 of page 3)

3.2 Collection of BSG protein level data

BSG protein levels were evaluated with the criteria (listed in **Supplementary Table 3**) for staining intensity and quantity scores**.** BSG protein levels were evaluated by the total immunohistochemical score, the product of the staining intensity, and quantity scores. All protein level scores for the 109 samples collected from THPA are shown in **Supplementary Table 2**.

Supplementary material 3. The criteria for staining intensity and the quantity scores.

Scores	Immunohistochemical staining features for BSG protein	
	Staining intensity	Quantity
0	Negative	None
1	Weak	<25%
2	Moderate	25%-75%
3	Strong	>75%

For Figure 2F (the previous Figure 3A), the normal tissues do not come from the same patients as the tumor samples, and thus it is not feasible to perform a paired (please see below the response to comment 5.1).

6. In the results section 4.5, line 182-196 the authors are evaluating the correlation between the BSG expression with DNA methyltransferases, Mismatch repair genes and immune cells. Particularly for

Figure 8C, clarification is needed:

6.1 What the level of immune cells means in the correlations with BSG expression, is this a frequency among CD45+ cells (leukocytes)? Are all these immune cells infiltrating the different types of tumors? It will be interesting if the authors could show the frequency of CD45+ cells in each tumor, and highlight that some tumor will have less infiltration of CD45+ cell, and this could be due to high expression of BSG. Those tumor with higher infiltration of CD45+ cells should have less expression of BSG.

Response:

(1) In the correlations with BSG expression, the levels of immune cells mean the infiltration levels of six types of immune cells (B cells, CD4+ T cells, CD8+ T cells, neutrophil, macrophage, and dendritic cells) rather than $CD45^{+}$ leukocytes. CD45 is known as a common antigen for leukocytes such as lymphocytes. In our study, we focus on only six types of immune cells instead of all leukocytes; for example, we did not analyze eosinophils.

(2) To be honest, we cannot directly answer the question "Are all these immune cells infiltrating the different types of tumors" based on the current results. Instead, in this revision, we attempted to analyze and find the difference in infiltration levels of the six types of immune cells between cancers and their controls (please see below).

4. Results (Line 179–288 of page 7)

4.5 The landscape of BSG mutations and the expression correlation of BSG with DNMT, MMRs, and immune microenvironment

In the aspect of immune filtration levels, *BSG* expression showed a negative correlation with nearly all six types of immune cells (B cells, $CD4^{+}$ T cells, $CD8^{+}$ T cells, neutrophil, macrophage, and dendritic cells) in THCA, CHOL, and SKCM (p < .05, **Figure 7C***)* rather than other cancers (**Supplementary Figure 5**). For nine kinds of cancers (BLCA, BRCA, KICH, LIHC, LUAD, LUSC, PRAD, THCA, and UCEC), both low infiltration levels of at least one of six types of immune cells and upregulated *BSG* expression were detected in cancer tissues (**Supplementary Table 6**), suggesting that the less infiltration of specific immune cells may be due to high expression of *BSG*. Notably, decreasing levels of all six types of cells and highly expressed *BSG* can be observed in LUSC (**Supplementary Table 6**), highlighting that *BSG* was likely to affect the six immune cell levels in LUSC patients.

(3) According to your professional suggestions, we have attempted to evaluate the abundance of immune cells in cancers and their control tissues. As a result, in some cancers, less infiltration of immune cells can be detected in some cancers with upregulated BSG expression (please see above). However, no decreasing infiltration of immune cells has been observed in cancers with downregulated BSG expression.

6.2 The authors should include some information in the methods for how these immune cells were

identified, what technique was used (for example, flow cytometry), and some information of the markers employed to identify each immune cell.

Response: In this revision, we have provided details about the immune cell-related data in the method section (please see below).

3. Materials and Methods (Line 125–132 of page 3)

3.4 Alternations, DNA methyltransferases, and the immune microenvironment of BSG in cancers

Based on the deconvolution method, the TIMER (Li et al. 2017, Li, Fu, et al. 2020) algorithm makes it feasible to calculate the immune abundance of six kinds of immune cells (B cells, $CD4^+$ T cells, $CD8^+$ T cells, neutrophil, macrophage, and dendritic cells) by whole-gene expression data. The other algorithm, ESTIMATE (Yoshihara et al. 2013), enables researchers to evaluate the immune abundance of immune cells and immune stromal based on gene expression data matrix. With the ESTIMATE algorithm, the abundance of immune stromal, immune cells, and tumor purity are reflected by the stromal (for stromal cells), immune (for immune cells), and ESTIMATE scores (for tumor purity).

6.3 Ideally, authors should adjust the scale for some of the correlation plots, since some correlations are out of scale, I suggest applying to this data an outlier's removal, to identify is some of the samples could be outliers and driving some of the results from these correlations.

Response: Thank you for your valuable advice. Some points we desired to state for our study:

(1) For parameter tests such as the Pearson test, the calculation results are greatly affected by abnormal values. Thus, we perform all the correlation analyses in this study using the Spearman correlation coefficient rather than the Pearson correlation coefficient. Indeed, the Spearman coefficient is acceptable for calculating data with a few outliers (Schober et al., *Correlation Coefficients: Appropriate Use and Interpretation*. Anesth Analg. 2018 May;126(5):1763-1768. PMID: 29481436).

(2) Taking Figure 7C as an example, the results before and after the removal of abnormal values based on percentiles (<25% or >75%) have little difference in the Spearman correlation coefficient (please see below).

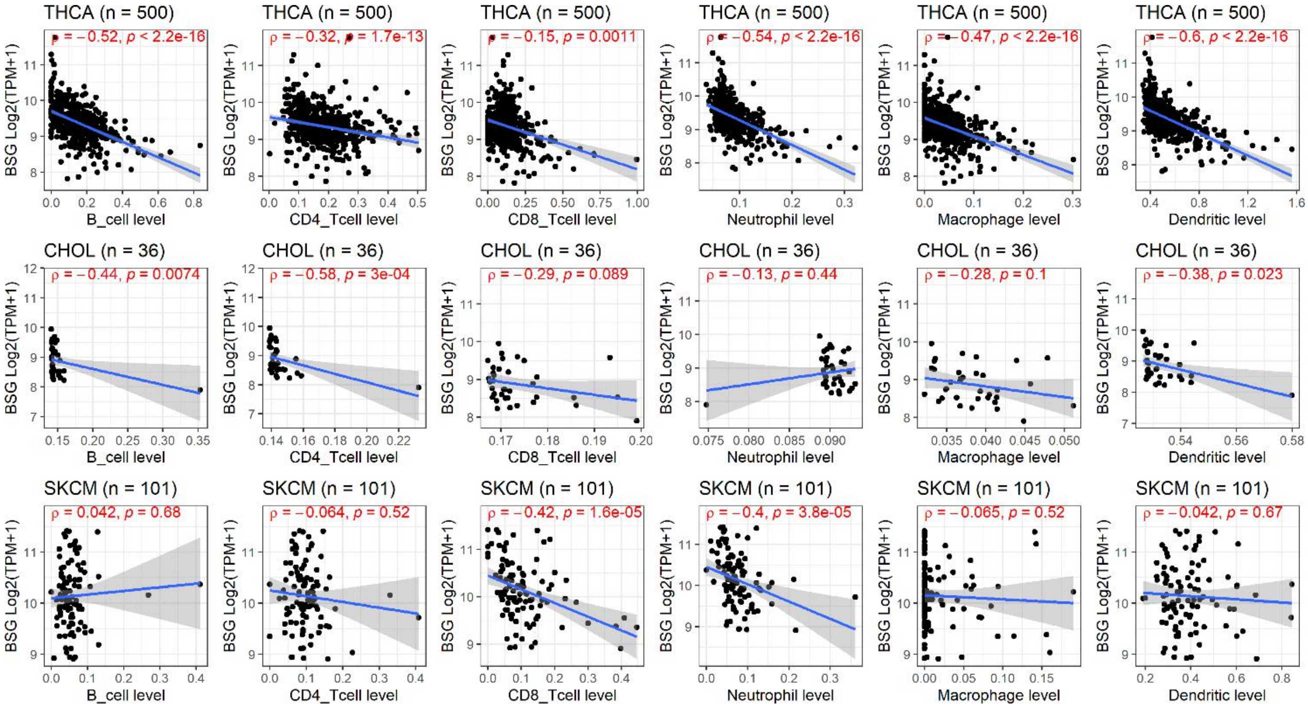

Figure 7C. Without removing the abnormal values, BSG expression is negatively associated with nearly all six types of immune cells in the three cancers. (Notes: the figure shown here is just for review)

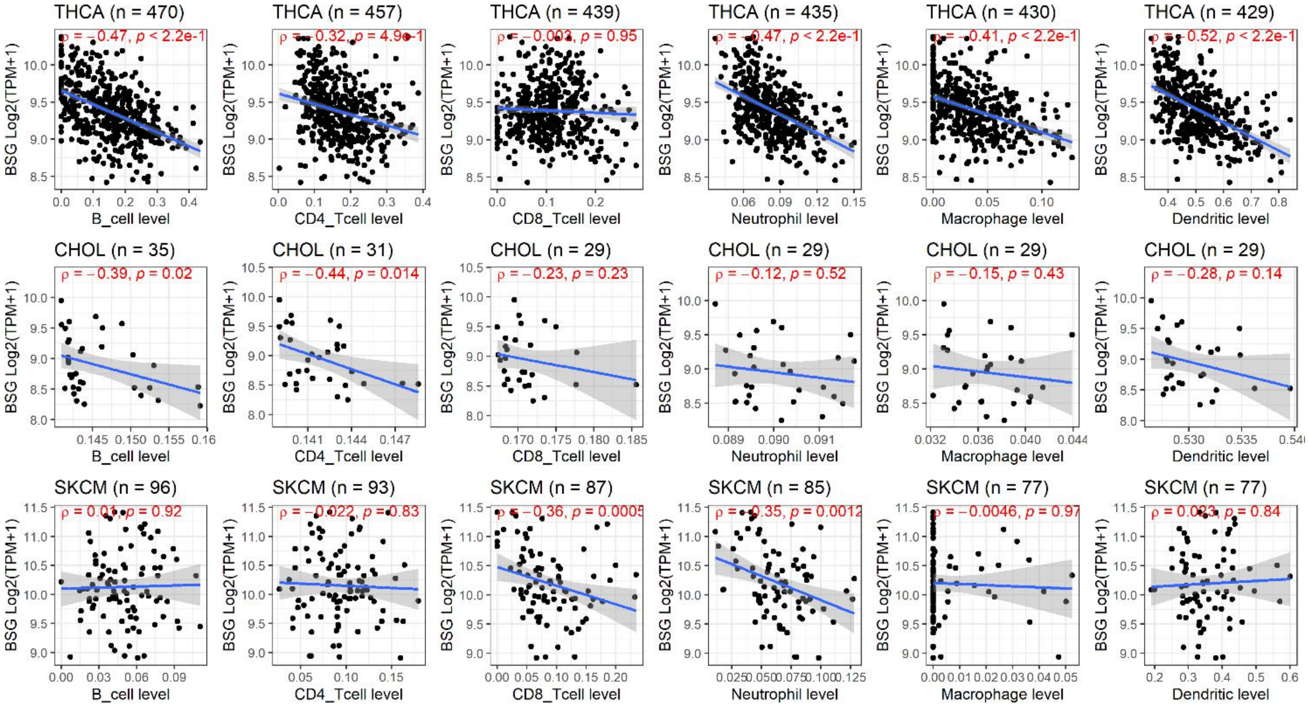

Figure A. After removing the abnormal values based on percentiles (<25% or >75%), BSG expression is also negatively associated with nearly all six types of immune cells in the three cancers. (Notes: Figure A is just for review)

(3) The abnormal value in the correlation analysis has also been not removed in previous research, such as studies of Peng et al. (*A Pan-Cancer Analysis of SMARCA4 Alterations in Human Cancers*.

Front Immunol. 2021 Oct 4;12:762598. PMID: 34675941) and Liu et al. (*A Comprehensive Prognostic and Immune Analysis of SLC41A3 in Pan-Cancer*. Front Oncol. 2021 Jan 14;10:586414. PMID: 33520701).

Taken together, may we keep the current results without removing abnormal values? If so, we have adjusted the scale for related plots by adjusting the coordinate axis (please see below).

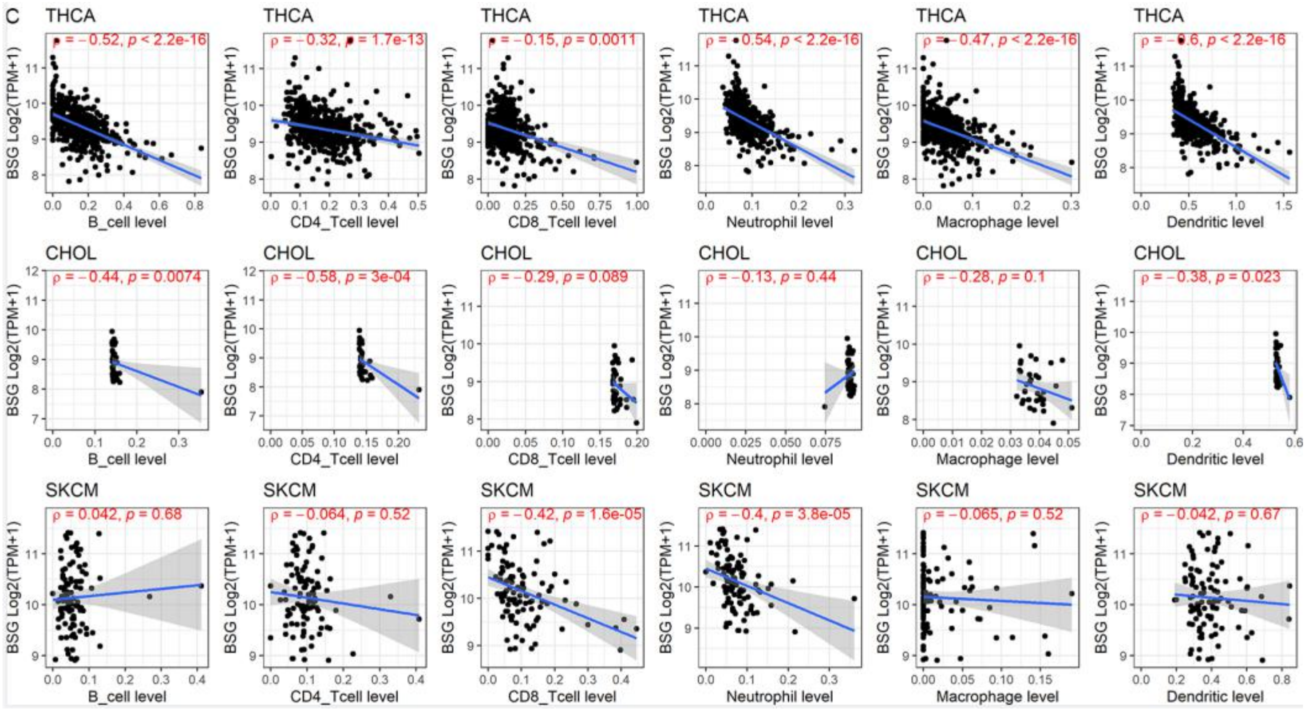

Figure B. Without adjusting the coordinate axis, some correlation plots are out of scale (e.g., plots for CHOL). (Notes: Figure B is just for review)

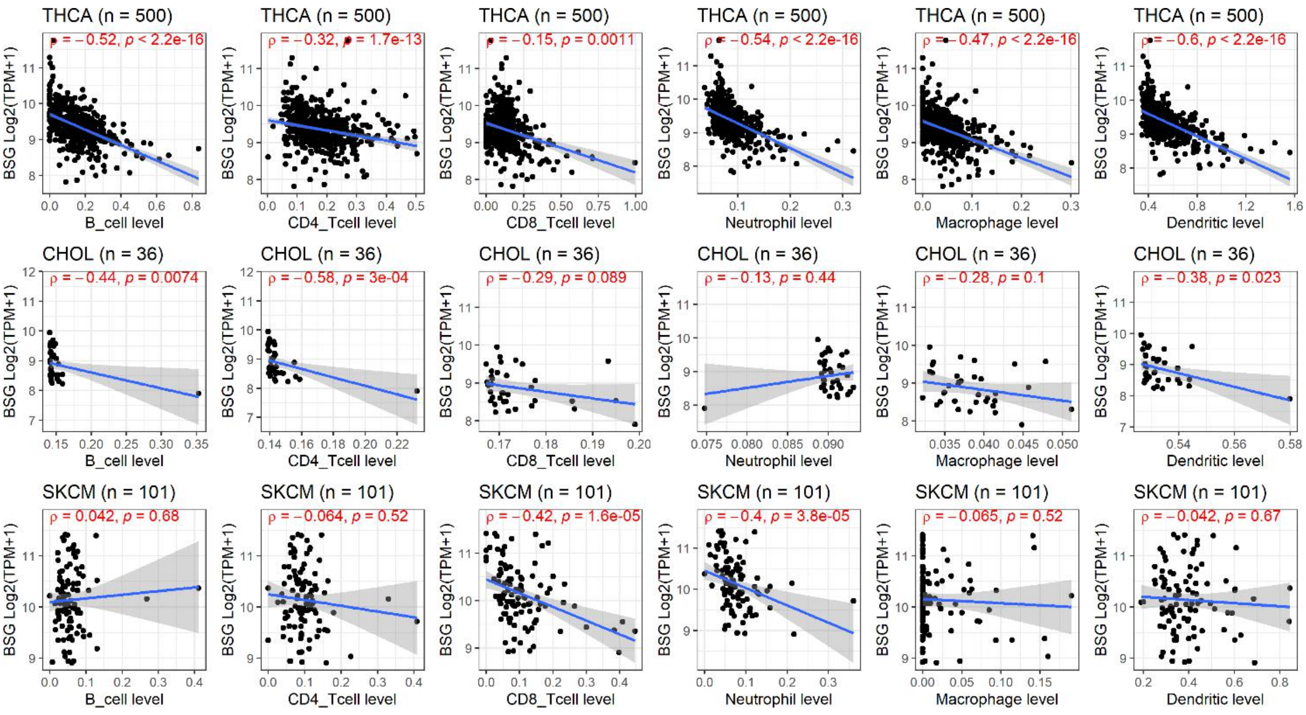

Figure 7C. After adjusting the coordinate axis, no correlation plots are out of scale (e.g., plots for

CHOL). (Notes: Figure B is just for review). (Notes: the figure shown here is just for review)

7. For figure 10A, line 209-219 for the GSEA analysis authors should show examples of correlations between the expression of BSG and top leading-edge genes in some of the pathways highlighted in the main text, for example genes that are in the cytochrome P450 and JAK-STAT signaling pathway to understand if the genes in each pathway have the same correlation as the hallmark pathway.

Response: Thank you for your professional advice. We have added the content in this revision. Please allow us to show it below:

4. Results (Line 309–332 of page 8)

4.7 GSEA and drug susceptibility of BSG in cancers

The study investigated potential mechanisms of *BSG* in 32 cancers based on KEGG signaling pathways by gene set enrichment analysis (GSEA). *BSG* was likely an essential factor in the occurrence and development of ESCA, KICH, KIRC, KIRP, PAAD, and THCA by complex mechanisms, as it was identified to involve no fewer than five signaling pathways (**Figure 9A**). Totally, up to 39 of KEGG signaling pathways were found for *BSG* in 32 cancers in this study (**Supplementary Table 7**). In at least five cancers, *BSG* was shown closely related to three pathways—olfactory transduction, metabolism of xenobiotics by cytochrome P450, and hematopoietic cell lineage (**Supplementary Table 7**), indicating the gene may play its roles in multiple human cancers. Moreover, some pathways such as drug metabolism cytochrome P450 and JAK-STAT signaling pathway were also found in the GSEA of *BSG* (**Supplementary Table 7**). For the top seven essential signaling pathways (olfactory transduction, etc.) presenting statistical significance in at least four cancers, correlations of their leading-edge genes with *BSG* expression were explored. For instance, for the olfactory transduction pathway demonstrating statistical significance in 15 cancers (KIRC, etc.), 21 leading-edge genes were found in the pathway. The relationship between them with *BSG* expression was explored in the 15 cancers. As a result, the significant (positive or negative) relevance of *BSG* expression with the 21 leading-edge genes was identified in certain types of the 15 cancers based on the Spearman rank correlation analysis ($p < .05$; **Supplementary Figure 8A**). A similar analysis was carried out for the other six essential pathways—hematopoietic cell lineage, metabolism of xenobiotics by cytochrome p450, drug metabolism cytochrome p450, JAK-STAT signaling pathway, leishmania infection, and pentose and glucuronate interconversions; as a result, *BSG* expression primarily showed negative correlations with leading-edge genes of the six signaling pathways ($p < .05$; **Supplementary Figures 8B–G**), indicating the roles of *BSG* played in specific cancers may be due to due to the disorder of these pathways.

Minor points:

1. The current title "A systematic analysis of COVID-19-related BSG in pan-cancer" does not reflect conclusions backed by data in this study. Authors should modify the title and highlight the fact that BSG is a key element for the infection and progression of COVID-19 and may serve as a novel target

for the effective treatment against COVID-19 and human cancer.

Response: According to your advice, we have changed the title to “Pan-analysis Reveals COVID-19-related BSG as a Novel Marker for Treatment and Identification of Multiple Human Cancers”.

2. Line 25-26. In the abstract in the methods section, it is not clear how many samples were included in the study, since they mention “a total of 1,8952 samples from multiple centers was included in our research for the comprehensive investigation of the differences in BSG expression between human organs, cancer cells, cancers tissues, and normal tissues”. Is it 18,952 samples? Clarification is needed to understand the number of participants used in this study.

Response: Thank you for your advice. There were 18,952 samples included in our study before this revision, and the number “1,8952” is a mistake. Indeed, some samples have been excluded in this revision, as they were included by mistakes previously. The sample number of our study is 18,911, and details of them can be viewed in Supplementary Table 1. The revised text can be seen below:

Abstract (Line 21–27 of page 8)

Methods: A total of 18,911 samples from multiple centers were included in our research for the comprehensive investigation of the differences in BSG expression between human organs, cancer cells, cancers tissues, and normal tissues.

3. Line 135-137, in Figure 2A, what does the dashed line indicate? Clarification is needed, authors need to add this in the figure legends.

Response: The dashed line in Figure 2A represents the average value (equaling to 8.549) of BSG expression in all the organs detected in our study. Clarification about this has been added to figure legend (please see below).

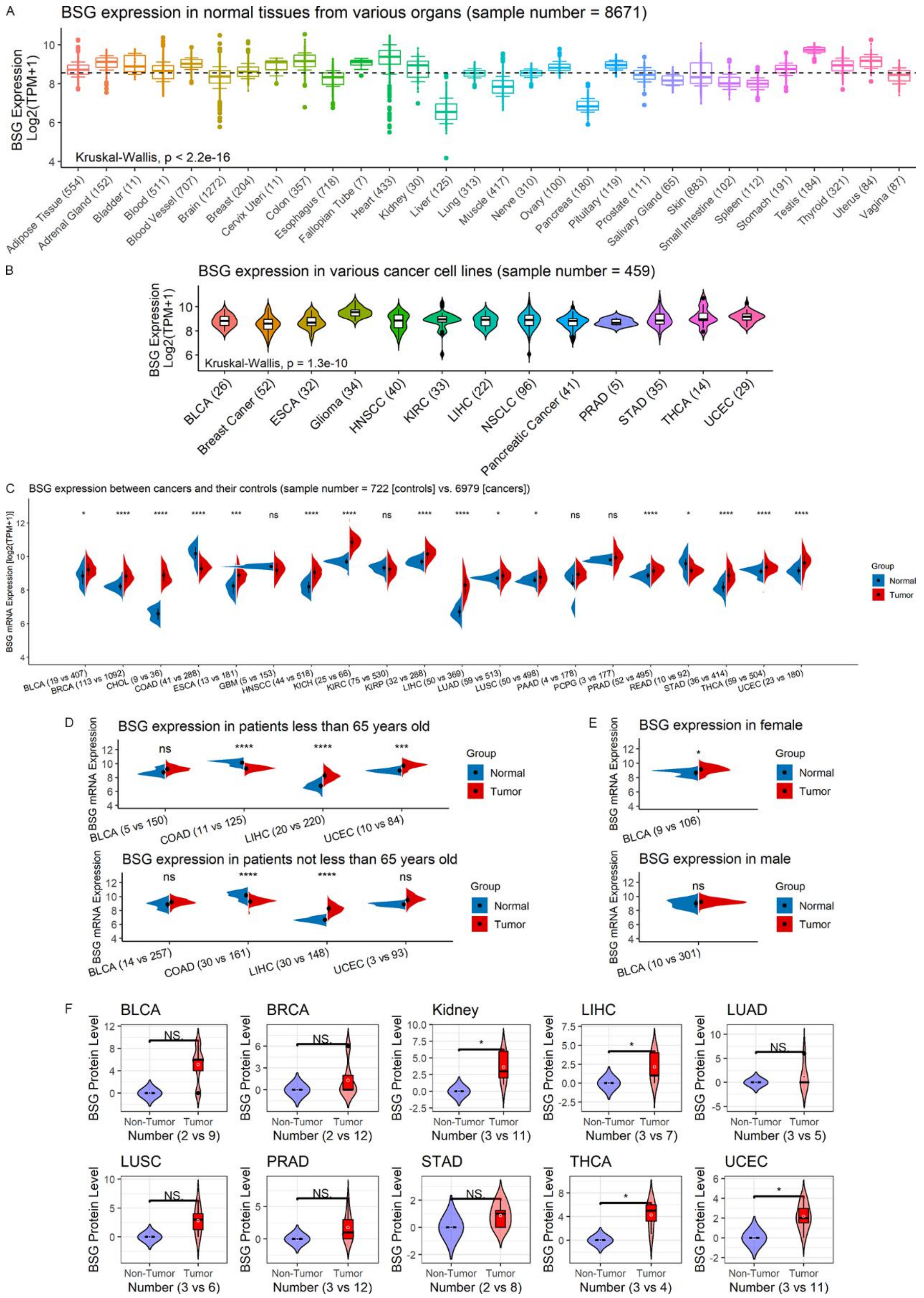

Figure 2. BSG expression in normal tissues and pan-cancer at mRNA and protein levels. Panel A: Distinct BSG expression in various normal tissues of humans; the dashed line represents the average value (equaling to 8.549) of BSG expression in all normal tissues shown in this panel. Panel B: Different BSG expressions in various cancer cell lines of humans. Panel C: Different BSG expressions between cancer tissues with their normal tissues. Panels D–E: The hierarchical analysis of BSG expression differences in some cancers and their control tissues. Panel F: Elevated BSG

levels in several cancers were validated at the protein level. For panels A and B: the value in parentheses represents the number of samples. For panels C–E: the two values (e.g., "n = 19 vs 407" for BLCA) in parentheses represent the numbers of controls and cancers, respectively. For panels C–F: each p-value in this figure is based on the Wilcoxon rank-sum test, and then p-values in panels C–E are adjusted by multiple comparations with the false discovery rate method; $^{*}p < 0.05$, $^{**}p < 0.01$, $^{***}p < 0.001$. BLCA, bladder cancer; ESCA, esophageal carcinoma; HNSCC, head and neck squamous cell carcinoma; KIRC, kidney renal clear cell carcinoma; LIHC, liver hepatocellular carcinoma; NSCLC, non-small-cell lung carcinoma; PRAD, prostate adenocarcinoma; STAD, stomach adenocarcinoma; THCA, thyroid carcinoma; UCEC, uterine corpus endometrial carcinoma; BRCA, breast invasive carcinoma; CHOL, cholangiocarcinoma; COAD, colon adenocarcinoma; KICH, kidney chromophobe; KIRP, kidney renal papillary cell carcinoma; LUAD, lung adenocarcinoma; LUSC, lung squamous cell carcinoma; READ, rectum adenocarcinoma.

4. Line 149. The authors mentioned that "at protein levels, ten types (with sufficient samples) of 16 cancers listed above were investigated in this study". The authors should clarify what is a sufficient number of samples and why they did not include the other 6 types of cancer.

Response: In this revision, we have stated the number of samples and explained why the other six kinds of cancers were excluded (please see below).

4. Results (Line 206–207 of page 5)

4.1 Differently expressed BSG in human cancers

Among the 16 cancers listed above, BSG protein levels were investigated in ten types, as there were at least two cancer samples and two normal samples for the ten.

5. Figure 3A should be part of Figure 2 to keep the reader connected to the storyline of the manuscript and Figure 2B should be different figure, since the authors are focusing in the relevance between BSG expression and clinical parameters.

Response: Thank you for your comments, and we have changed Figure 3A to the new Figure 2F. In terms of that "Figure 2B should be different figure, since the authors are focusing in the relevance between BSG expression and clinical parameters", may you identify the previous Figure 2D instead of Figure 2B? If so, the previous Figure 2D has been changed to Figure 3B (Please see below).

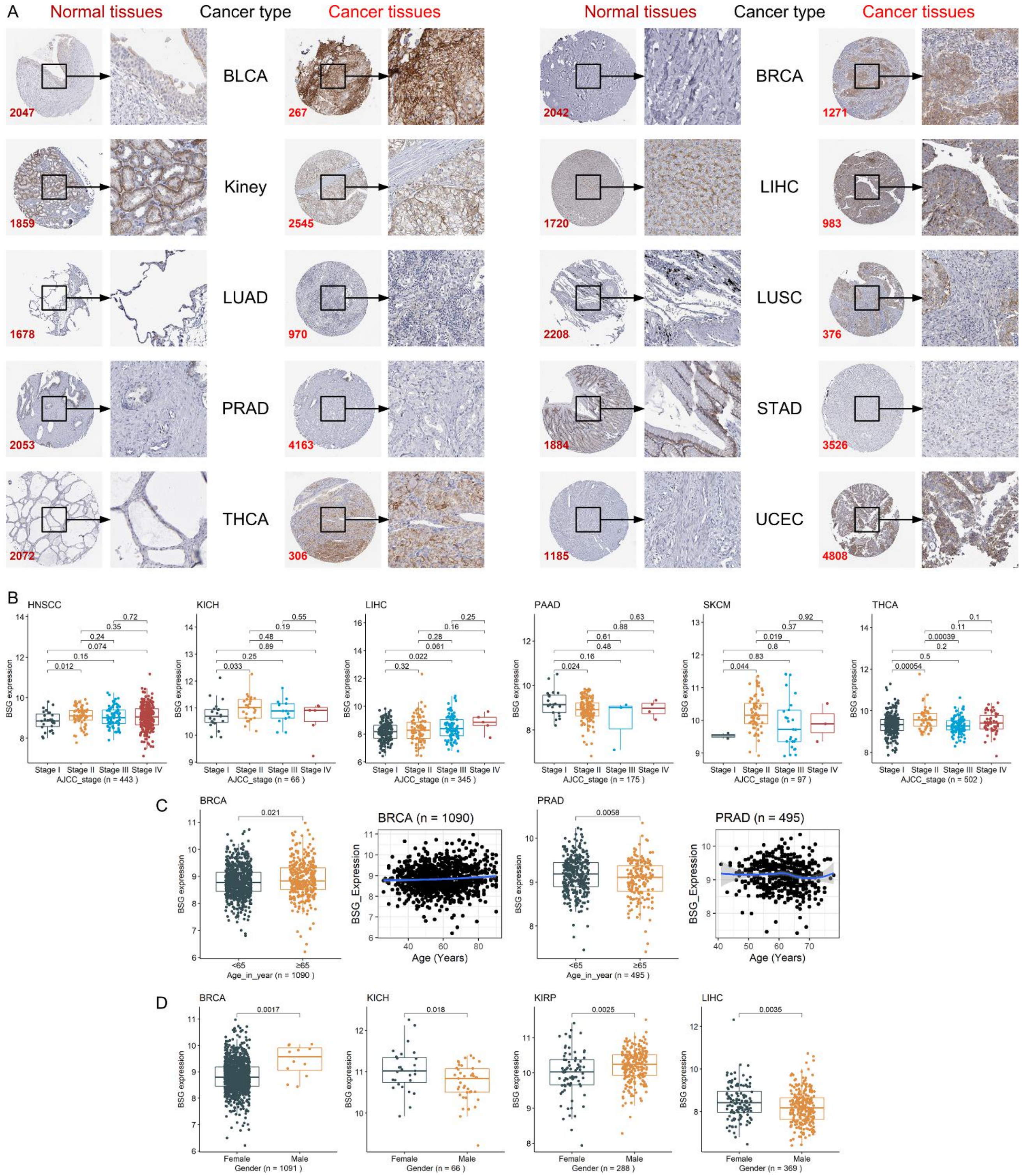

Figure 3. The staining intensity of the anti-BSG antibodies in the ten cancers and the correlations between BSG expression and clinical parameters. Panel A: Under the microscope, the staining intensity of the anti-BSG antibodies in the ten cancer tissues is stronger than that in normal tissues; images are available from v21.0.proteinatlas.org. In panel A, the value of the lower-left corner of the images (e.g., 2047 for BLCA) is the identity document of patients or normal individuals. Panels B–D: The close correlations between BSG expression with clinical parameters; all p-values in the three panels were based on the Wilcoxon rank-sum test. The two plots with a blue curve of panel C are drawn based on the locally weighted regression. The value in parentheses (e.g., “n = 443” for

HNSCC) represents the number of samples. BLCA, bladder cancer; BRCA, breast invasive carcinoma; LIHC, liver hepatocellular carcinoma; LUAD, lung adenocarcinoma; LUSC, lung squamous cell carcinoma; PRAD, prostate adenocarcinoma; STAD, stomach adenocarcinoma; THCA, thyroid carcinoma; UCEC, uterine corpus endometrial carcinoma; HNSCC, head and neck squamous cell carcinoma; KICH, kidney chromophobe; PAAD, pancreatic adenocarcinoma; SKCM, skin cutaneous melanoma.

6. Line 155, Figure 4 continue showing examples of the staining intensity of the anti-BSG antibodies in the different cancer tissues as it was shown in Figure 3. The authors should consider showing this data in a supplemental figure, this should be considered as a supplemental figure 1.

Response: We agree with you, and considering the new Figure 3 showing the same item and having sufficient space, may we put the panels of the previous Figure 4 to the revised Figure 3 (please see above)?

7. Line 157, the authors mention that "Different clinical parameters may result in the different prognosis of cancer patients". Clarification is needed here for readers to think critically on how these clinical parameters were used to define the stages of cancer.

Response: We have provided some examples (please see below), and we hope this will help readers to understand it more easily.

4. Results (Line 218–223 of page 6)

4.2 Relevance between BSG expression and clinical parameters

Different clinical parameters may result in the different prognoses of cancer patients. For example, patients with cervical cancer and with advanced AJCC stages tended to have poor prognoses (Olawaiye et al. 2021). The other example was that elder age, and male gender were risk factors for patients with oral squamous cancers (Oh et al. 2021). Thus, the relationship between *BSG* expression and the three clinical parameters—AJCC stages, age, and gender—of patients was explored in neoplasms with the clinical parameters data

8. Line 159, authors need to define AJCC, American Joint Committee on Cancer.

Response: Thanks for your kind reminder, and we have defined AJCC with its complete spelling in the materials and methods section:

3. Materials and Methods (Line 92–93 of page 3)

3.1 Collection of BSG mRNA expression, clinical characteristics, and prognosis data

Three kinds of clinical characteristics—American Joint Committee on Cancer (AJCC), age, and gender—were obtained from the Xena database.

9. For the results in Figure 2D a LOESS age trajectory could help to visualize the changes in BSG levels with age.

Response: Thank you for your professional advice. We have drawn such LOESS (locally weighted regression) age trajectory plots, which can be seen below:

4. Results (Line 223–230 of page 6)

4.2 Relevance between BSG expression and clinical parameters

The immediate relevance of *BSG* expression with AJCC stages was found in HNSCC, KICH, LIHC, PAAD, SKCM, and THCA ($p < .05$; **Figure 3A**), while such a phenomenon was not in the other cancers (ACC, etc.) (**Supplementary Figure 1**). Higher *BSG* expression was detected in elderly (65 years old or older) BRCA patients ($p < .05$), and based on the LOESS (locally weighted regression) age trajectory, older patients tend to be found increasing *BSG* expression levels (**Figure 3B**). Elevated *BSG* expression was generally be observed in young (< 65 years old) PRAD patients ($p < .05$); however, the LOESS age trajectory suggested some fluctuations in the correlation between age and *BSG* expression (**Figure 3C**).

10. On the main text, authors need to add the name of the different types of cancer and add the acronym next to it, but a definition is needed for readers to clearly understands. This need to added in the figure legends.

Response: We have added full names for various cancers, and the same contents have also been shown in the figure legends. Some examples are as follows:

4. Results (Line 183–195 of page 5)

4.1 Differently expressed BSG in human cancers

Based on GTEx data, *BSG* expression was various in different organs detected: its high expression was observed in normal tissues of the adrenal gland, heart, uterus, and so on, while significantly low expression of *BSG* was found in the liver and pancreas ($p < .05$; **Figure 2A**). An analysis based on CCLE data demonstrated the different distribution of *BSG* in various cancer cell lines ($p < .05$;

Figure 2B). In the observed 20 cancer types, the *BSG* expression levels of 16 cancers differed from that of the corresponding control samples. In detail, upregulated *BSG* expression was observed in 14 cancers—bladder cancer (BLCA), breast invasive carcinoma (BRCA), cholangiocarcinoma (CHOL), esophageal carcinoma (ESCA), head and neck squamous cell carcinoma (HNSCC), kidney chromophobe (KICH), kidney renal papillary cell carcinoma (KIRP), LIHC, LUAD, LUSC, prostate adenocarcinoma (PRAD), stomach adenocarcinoma (STAD), thyroid carcinoma (THCA), and uterine corpus endometrial carcinoma (UCEC); downregulated *BSG* expression was detected in two cancers—colon adenocarcinoma (COAD) and rectum adenocarcinoma (READ) ($p < .05$; **Figure 2C**).

11. Line 167, authors need to define "OS and DSS" to keep the reader attention and to improve the interpretation of the text. They should add this in the text "Overall survival (OS) and Disease-specific survival (DSS)".

Response: Thanks for your kind reminder. We have added this content to the text (please see below).

4. Results (Line 247–256 of pages 6–7)

4.3 Prognosis value of BSG expression in cancers

We investigated the prognosis value of *BSG* expression in various cancers. Based on the results of univariate Cox analyses and Kaplan-Meier curves, in terms of both overall survival (OS) and disease-specific survival (DSS), *BSG* expression was relevant to shorted survival time in LGG, LUAD, and UCS (hazard ratio [HR] > 1, $p < .05$) and to longer survival time in KIRP (HR = 0.364, $p < .05$) (**Figure 4A–D**). Moreover, high *BSG* expression was also a risk factor of DSS for BRCA patients (HR = 1.459, $p < .05$) (**Figures 4C–D**). In disease-free interval (DFI), overexpression of *BSG* represented unfavorable prognosis for patients with BRCA, LGG, PRAD, and UCS (HR > 1, $p < .05$) (**Figures 5A–B**). In progression-free interval (PFI), increased *BSG* expression was associated with poor PFI for BRCA, LGG, and UCS (HR > 1, $p < .05$), while it demonstrated a favorable prognosis for PAAD and THYM patients (HR < 1, $p < .05$) (**Figures 5C–D**).

12. Line 170, authors need to define "DFI", as disease free interval. Line 171, authors need to define "PFI", as progression free interval.

Response: We have revised them according to your kind reminder (please see above).

13. Line 182-196, in the results section 4.5, the authors are evaluating the correlation between the BSG expression with DNA methyltransferases, Mismatch repair genes and immune cells. In the main text the authors mentions that they are using only 5 MMRs (MLH1, MSH2, MSH6, PMS2, and EPCAM) clarification is needed for the readers to understand why the authors are using only these

examples, they should explain why this is important and what is the biological meaning of the correlation results with BSG, they also need to cite other reports using these MMRs.

Response: Thank you for your professional suggestions. We have explained why the five MMR genes are important and the biological meaning of the correlation results with BSG. We also cited some related studies (please see below).

4. Results (Line 268–278 of page 7)

4.5 The landscape of BSG mutations and the expression correlation of BSG with DNMT, MMRs, and immune microenvironment

In the landscape of *BSG* mutation, missense mutation was the most common alternation, and UCEC possessed the most significant number (3.4%) of mutation (**Figure 6C**). The expression of three DNMT (DNMT1, DNMT3A, and DNMT3B) was positively correlated with *BSG*, especially in GBM, LIHC, and PRAD (**Figure 7A**). The four proteins encoded by MLH1, MSH2, MSH6, and PMS2 are the prominent roles in the MMR procession. The four genes and another MMR gene, EPCAM, are the characteristics of Lynch syndrome (a cancer-prone syndrome) (Latham et al. 2019, Baretti and Le 2018). Thus, the five MMR genes (MLH1, MSH2, MSH6, PMS2, and EPCAM) were investigated in cancer-related studies (Liu et al. 2020, Deshpande et al. 2020), which were also selected in our research. As a result, *BSG* expression showed significantly related with the five MMR genes in multiple cancers, especially in OV and GBM (**Figure 7B**), implying that *BSG* may affect the expression of MMRs in certain cancers, which needs experimental validation.

15. Line 208, authors need to correct “Supplementary Figure 3” for “Supplementary Figure 1”, they do not have 3 supplementary figures.

Response: Thanks for your kind reminder. In this revision, we have added some supplementary figures, and thus, the previous “Supplementary Figure 3” has been changed to “Supplementary Figure 7”.

16. Line 273, 274, and 277, in the discussion the authors need to correct “COIVD-19 patients” for COVID-19 patients.

Response: We are sorry for these mistakes and have corrected them (please see below).

5. Discussion (Line 386–390 of pages 9–10)

BSG may be an important potential target for treating various cancers. Constantly, the function of immunocytes facilitates the body’s antivirus and anti-tumor biological process. Thus, decreasing levels of immune cells will cause deterioration of COVID-19 patients and cancer patients. Indeed,

previous research suggests lymphocyte reduction is an indicator of COVID-19 patients and various cancer patients (Li, Guo, et al. 2020, Tan et al. 2020).

17. Line 295, in the discussion the authors need to correct "GEA" for GSEA.

Response: We have revised the mistake (please see below).

5. Discussion (Line 409–412 of page 10)

Another example was that *BSG* was also considered to be a vital molecule causing multiple cancer resistance (Landras and Mourah 2020); combined with the GSEA results of this study, this may be related to *BSG* participating in drug metabolism cytochrome P450 and other pathways.

Check List

a. Is the quality of the figures and tables satisfactory? No

Response: We have improved the quality of all figures based on the comments of the reviewers and editors.

b. Does the reference list cover the relevant literature adequately and in an unbiased manner? Yes

Response: Thanks for your professional and kind suggestions.

c. Are the statistical methods valid and correctly applied? (e.g. sample size, choice of test) No

Response: In terms of statistical methods, we have performed some changes, such as using adjusted p value of FDR rather than raw p value in Figure 2C. All sample information of sample size, choice of test, and the corresponding details have been indentified in the Materials and Methods section and figure legends. If some other changes are needed in terms of statistical methods, we will be glad to consider that.

d. Is a statistician required to evaluate this study? Yes

Response: Thanks for your comment.

e. Are the methods sufficiently documented to allow replication studies? No

Response: According to your comment, we have provided as much detail as possible in the Materials and Methods section, figures, the supplementary tables, and supplementary figures.

QUALITY ASSESSMENT:

Rigor: 4

Response: Thanks.

Quality of the writing: 3

Response: Thanks for your comment. We have carefully addressed language editing with the help of a native English speaker in our team.

Overall quality of the content: 3

Response: Thank you for your professional suggestions. We believed that the manuscript has been improve greatly in this revision with help of you. Thus, we have added one sentence in the Acknowledgements section for the valuable suggestions and careful work done by you, that is, "We are deeply grateful to anonymous reviewers for their detailed work and thoughtful suggestions, which have contributed greatly to the improvement of this article." (Line 547–548 of page 16).

Interest to a general audience: 5

Response: Thanks a lot for your kind suggestion, and this greatly encouraged us.
